# Genome-wide identification, interaction of the MADS-box proteins in *Zanthoxylum armatum* and functional characterization of *ZaMADS80* in floral development

**DOI:** 10.3389/fpls.2022.1038828

**Published:** 2022-11-25

**Authors:** Ning Tang, Zhengyan Cao, Peiyin Wu, Xian Zhang, Juan Lou, Yanni Liu, Qiyao Wang, Yang Hu, Shuo Si, Xiaofan Sun, Zexiong Chen

**Affiliations:** ^1^ Chongqing Key Laboratory of Economic Plant Biotechnology, Chongqing University of Arts and Sciences, Chongqing, China; ^2^ College of Horticulture and Gardening, Yangtze University, Jingzhou, China; ^3^ College of Biology and Food Engineering, Chongqing Three Georges University, Chongqing, China

**Keywords:** *Zanthoxylum armatum*, MADS-box, protein interactions, sex determination, apomixis

## Abstract

As a typical dioecious species, *Zanthoxylum armatum* establishes apomictic reproduction, hence only female trees are cultivated. However, male and hermaphrodite flowers have recently appeared in female plants, resulting in a dramatic yield reduction. To date, the genetic basis underlying sex determination and apomixis in *Z. armatum* has been largely unknown. Here, we observed abortion of the stamen or carpel prior to primordium initiation, thus corroborating the potential regulation of MADS-box in sex determination. In *Z. armatum*, a total of 105 MADS-box genes were identified, harboring 86 MIKC-type MADSs with lack of FLC orthologues. Transcriptome analysis revealed candidate *MADSs* involved in floral organ identity, including ten male-biased *MADSs*, represented by *ZaMADS92/81/75*(AP3/PI-like), and twenty-six female-specified, represented by *ZaMADS80/49* (STK/AGL11-like) and *ZaMADS42* (AG-like). Overexpressing *ZaMADS92* resulted in earlier flowering, while *ZaMADS80* overexpression triggered precocious fruit set and parthenocarpy as well as dramatic modifications in floral organs. To characterize their regulatory mechanisms, a comprehensive protein-protein interaction network of the represented MADSs was constructed based on yeast two-hybrid and bimolecular fluorescence complementation assays. Compared with model plants, the protein interaction patterns in *Z. armatum* exhibited both conservation and divergence. ZaMADS70 (SEP3-like) interacted with ZaMADS42 and ZaMADS48 (AP3-like) but not ZaMADS40 (AP1-like), facilitating the loss of petals in *Z. armatum*. The ZaMADS92/ZaMADS40 heterodimer could be responsible for accelerating flowering in *ZaMADS92*-OX lines. Moreover, the interactions between ZaMADS80 and ZaMADS67(AGL32-like) might contribute to apomixis. This work provides new insight into the molecular mechanisms of MADS-boxes in sex organ identity in *Z. armatum*.

## Introduction

*Zanthoxylum armatum* DC, an evergreen dioecious tree belonging to the genus Zanthoxylum of family Rutaceae, is native to China and mainly distributed throughout Asia. This species, so-called Green Huajiao, has long been consumed as a spice and a traditional herbal medicine, attracting increasing attention very recently (Liu et al., 2022). Among them, *Z*. *armatum* cv ‘Jiuyeqing’ is the most famous variety for its short juvenility, high yield and rich aroma, widely cultivated in Chongqing, Sichuan, Yunnan and Guizhou provinces in China. Generally, because of its reproduction pattern is apomixis, cultivated *Z. armatum* plants are female (Zhang et al., 2020). However, very recently, male flowers have appeared in the female plants of *Z. armatum* cv Jiuyeqing, (**Figure S1**) thus resulting in a dramatic decline in fruit yield. Therefore, it is of great significance to elucidate the molecular mechanism underlying sex determination and apomixis in *Z. armatum*.

In the majority of dioecious angiosperm species, including *Silene latifolia*, *Asparagus officinalis* and *Diospyros lotus*, flowers are initiated with hermaphroditic primordia, followed by abortion or arrest of the sex organs during their development. In this case, sex determination might undergo the regulation of the genes downstream of floral organ identity. Some dioecious plants such as spinach, exhibit unisexuality with early abortion of carpels or stamens, possibly controlled by homeotic-like mechanism in the early flower primordium stage (Di Stilio et al., 2005; Akagi et al., 2014; Feng et al., 2020). However, to date, the anatomical features throughout male and female flower development in *Z. armatum* have not been clearly illustrated, and yet the master regulators and molecular pathways controlling sex determination remain unexplored. Based on genetic data mainly from *Arabidopsis* and petunia, the identity of four-whorled floral organs is specified by tetrameric complexes composed of MADS-box proteins, termed as ABCDE model (Theißen et al., 2016). In brief, the A function is mainly mediated *via* APETALA1 (AP1), which is also involved in floral meristem specification, while the B function is exerted *via* APETALA3 (AP3) and PISTILLATA (PI) and the C function by AGAMOUS (AG). Additionally, class D specifies the ovule identity represented by SEEDSTICK (STK/AGL11), SHATTERPROOF1 (SHP1/AGL1), and SHATTERPROOF2 (SHP2/AGL5). Class E genes SEPALLATA1 (SEP1/AGL2), SEP2/AGL4, SEP3/AGL9, and SEP4/AGL3 are required for the formation of all whorls of floral organs. For floral organ determination, class A+E genes specify sepals, A+B+E specify petals, B+C+E specify stamens, C+E specify carpels and C+D+E specify ovules, which function by protein interactions with each other and formation of tetrameric complexes (Yan et al., 2016). Over the last decade, despite the rapid advances in uncovering the involvement of MADS in sex determination in some species with unisexual flowers, relevant studies on *Z. armatum* are severely limited due to the lack of available genome information and the absence of male plants (usually difficult to see) (Zhang et al., 2020; Wang et al., 2021).

Under natural environment, the female trees of *Z. armatum* can successfully trigger fruit set, bypassing fertilization *via* apomixis. Similar to citrus, *Z. armatum* is a typical sporophytic apomictic plant in which adventitious embryos are developed from nucellar cells, leading to ovule development. Apomixis may occur when abortion of sexual reproduction is caused by the mutation of corresponding genes and thus regulated by a molecular framework similar to sexual reproduction. So far, several genes have been identified associated with different types of apomixis (Xu et al., 2022), of which, two MADS-box genes were noticed, including *DEFICIENSH* (*Deficiens* homologue, a B-class MADS box genes) in *Hieracium* (Guerin et al., 2000) and *AGL11* in *Z. bungeanum* (Fei et al., 2021). NOZZLE/SPOROCYTELESS, a key TF involved in apomixis, can be controlled by AP3/PI (Wuest et al., 2012) and AG (Ito et al., 2004). In addition, considering the commonalities between apomixis and parthenocarpy as well as the important roles of MADS-box in parthenocarpy (Subbaraya et al., 2020), MADS might play crucial roles in apomixis. However, the molecular basis underlying apomixis is largely unknown in *Z. armatum* (Wang et al., 2021).

Herein, to understand the morphological and genetic features, the anatomical structures of male- and female-flowers at different developmental stages were observed to clear the types of unisexual flowers. RNA-Seq analysis was employed to identify the total MADS-box transcription factors and the candidate MADS members involved in sex organ (stamen and pistil) identity. Moreover, functional characterizations and protein interactions were performed to reveal the possible involvements and molecular mechanisms of the candidates in floral organ initiation and development. Our findings lay the foundation for elucidating the molecular basis underlying sex determination and apomixis in *Z. armatum* and also provide new insights into inhibiting the occurrence of male flowers and elevating fruit yield for sustainable production.

## Materials and methods

### Plant materials

*Zanthoxylum armatum* DC cv. ‘Jiuyeqing’ used in this study was cultivated in the germplasm nursery of Chongqing University of Arts and Sciences, Yongchuan District, Chongqing, China. Flowers and fruits at different developmental stages were harvested from 5-year-old male and female plants of *Z. armatum*, respectively. In brief, three vital periods of floral differentiation and development in male plants were collected, including stamen primordia differentiation (MFS1), stamen initiation (MFS2) and floral development (MFS3). Eight important periods of pistil and fruit development in female plants were sampled, which includes fruit set and fruit development period. There were four stages in fruit set period, such as pistil primordia differentiation (FFS1), ovule initiation (FFS2), ovule development, approximately 10 days and 17 days after ovule initiation, marked as FFS3 and FFS4, respectively. Afterwards, fruit samples at four developmental stages were collected, that was 20, 50, 80, and 110 days after fruit set, named as FFS5, FS6, FFS7, and FFS8, respectively. All the samples were collected from 9 individual plants and tissues from 3 plants were pooled and set as one replicate. Three biological replicates were performed in each experiment. All the tissues were placed in tubes, frozen in liquid nitrogen and then stored at -80° C until further use.

### RNA-seq analysis

Total RNA from different tissues including the flower and fruit was isolated by using QIAGEN RNeasy Plant Mini Kit. 1 µg RNA was used as input material for library construction. Sequencing libraries were generated using NEBNext^®^ UltraTM RNA Library Prep Kit for Illumina^®^ (NEB, USA) following manufacturer’s recommendations. Briefly, mRNA was purified using poly-T oligo-attached magnetic beads. Then first and second strand cDNA were synthesized, DNA fragments were adenylated and ligated with NEBNext adaptor. Subsequently, cDNA fragments with preferentially 250~300 bp in length were purified with AMPure XP system (Beckman Coulter, Beverly, USA), size-selected, adaptor-ligated, and then PCR performed and its products were purified (AMPure XP system). Finally, the library preparations were sequenced on an Illumina Novaseq platform and 150 bp paired-end reads were generated.

RNA-seq data analysis including *de novo* transcriptome assembly, functional annotation and screening of differentially expressed genes (DEGs) were performed. Trinity software (https://github.com/trinityrnaseq/trinityrnaseq/wiki) was employed to pool and assemble all the clean reads. To obtain functional annotation, the assembled contigs and unigenes were aligned against six public databases (Nr, KEGG, GO, COG, Swiss-Prot, and Pfam) using BLASTx (e-value < 0.00001). DESeq2 was employed to identify DEGs from transcriptome data. FDR< 0.001 and the absolute value of Log2 ratio > 1 was used to judge the significance of gene expression difference (Love et al., 2014).

### Identification of putative MADS-box genes of *Z. armatum*

The transcriptome data conducted in this study was used to search for MADS-box transcription factors in *Z. armatum*. ORF Finder and ExPASy Translate tool were employed to identify the open reading frames (ORFs) and amino acids of the putative MADS-box genes. The query protein sequences were then subjected to NCBI BlastP analysis. Finally, all candidate MADS-box proteins were confirmed by comparison with the conserved domain against NCBI CDD (https://www.ncbi.nlm.nih.gov/cdd/) and Pfam (http://pfam.xfam.org/).

### Phylogenetic analysis of MADS-box genes

The *Arabidopsis* M-type MADS and MIKC MADS family protein sequences were downloaded from Plant Transcription Factor Database v5.0 (http://planttfdb.gao-lab.org/). The alignments of amino acid sequences of *AtMADSs* and *ZaMADSs* were performed using Clustal X. A phylogenetic tree was constructed using MEGA5.0 software by the Neighbor-joining method, with a bootstrap test replicated 1,000 times, the *p*-distance method and pairwise deletion. An online software Evolview v2 (https://www.evolgenius.info/evolview/) was employed to embellish the phylogenetic tree. MEME suite (http://meme-suite.org/tools/meme) was employed to analyze the conserved motifs of MADS proteins. And the parameters were set as follows: maximum number of motifs, 10; minimum width, 6; and maximum width, 50.

### Prediction of MADS protein‐protein interaction network

To obtain protein‐protein interactions among MADSs, the differentially expressed *MADS-box* genes among flower and fruit development were subjected to the online software UniProt (https://www.uniprot.org/), String (https://cn.string-db.org/), and GeneMANIA (http://genemania.org/).

### Transcriptional activation assay and protein interactions analysis by yeast two-hybrid

The GAL4-based yeast two-hybrid (Y2H) system was employed to assay the transcriptional activity of the central ZaMADSs of the protein interaction network, including ZaMADS40, ZaMADS42, ZaMADS50, ZaMADS70, ZaMADS80, ZaMADS89 and ZaMADS92. The full-length cDNA sequence of these *ZaMADSs* and their truncated versions were amplified by PCR and cloned into pGBKT7 vector (Takara, Japan) by using Takara In-Fusion HD cloning kit. Primers were listed in **Supplementary Table S1**. The recombined *ZaMADSs*-pGBKT7 plasmids were transformed into Y2H Gold yeast cells and spread onto SD/-Trp, SD/-Trp/X-α-gal and SD/-Trp/X-α-gal/AbA plates for transcriptional activation determination. Empty pGBKT7 vector was also transformed into the yeast cells as a negative control.

Y2H system was also carried out to investigate protein interactions. The full length or truncated *ZaMADSs* with no transcriptional activation activity above tested were used as baits. The full-length cDNAs of the different *ZaMADS* genes, including *ZaMADS40*, *ZaMADS42*, *ZaMADS48*, *ZaMADS50*, *ZaMADS54*, *ZaMADS57*, *ZaMADS67*, *ZaMADS70*, *ZaMADS74*, *ZaMADS75*, *ZaMADS89* and *ZaMADS95* were amplified using the primers listed in **Supplemental Table S1** and then cloned them into the pGADT7 vectors as preys. According to the predicted protein interaction network, each pair of bait and prey vectors were both transformed into yeast Y2H Gold strain (TaKaRa, Japan). The cotransformants were plated on SD/−Leu/−Trp medium, and then the positive transformants spread on SD/−Leu/−Trp/X-α-gal (DDO/X-α-gal), SD/−Leu/−Trp/X-α-gal/AbA (DDO/X-α-gal/AbA) and SD/−Ade/-His/−Leu/−Trp/X-α-gal/AbA (QDO/X-α-gal/AbA) plates for protein interactions test.

### Bimolecular fluorescence complementation analysis

The vectors of BiFC (pNC-BiFC-Ecn and pNC-BiFC-Enn) and protocol of the BiFC assay were performed as described in our previous study (Liu et al., 2022). In brief, the ORFs of *ZaMADS80* and *ZaMADS92* were ligated into the pNC-BiFC-Enn vector to generate nYFP fusion proteins, while the ORFs of *ZaMADS75*, *ZaMADS40* and *ZaMADS67* were ligated into the pNC-BiFC-Ecn vector to generate cYFP fusion proteins by using Nimble Cloning kit (Hainan Yitian Biotechnology Co., Ltd). The primers used were listed in **Supplementary Table S1**. We used the p2300-35S-H2B-mCherry-OCS (BioVector NTCC Inc.) recombinant plasmid as a marker for nuclear localization. The recombined plasmids were transformed into GV3101 (pSoup) and then the fusion proteins were co-expressed transiently in the leaves of *Nicotiana benthamiana*. After 72h of co-culture, the fluorescent protein signals were visualized by Leica DMi8 confocal laser scanning microscopy (Leica, Wetzlar, Germany) at Yangtze University.

### Overexpression of *ZaMADS80* and *ZaMADS92* and in tomato and *Arabidopsis*

To generate *ZaMADS80* and *ZaMADS92* overexpression (OX) plants, the ORF sequences of these two genes without the stop codon were amplified using TransStart FastPfu Fly DNA Polymerase (TransGen Biotech, Beijing, China) and primers listed in **Supplementary Table S1** and cloned into modified plant binary vector K303 under the CaMV 35S promoter through In-Fusion HD cloning method and a Gateway LR reaction, and then the plasmids K303-*ZaMADS80* and K303-*ZaMADS92* were transferred to *Agrobacterium tumefaciens* GV3101 for wild-type tomato (*Solanum lycopersicum* L. cv. Micro-Tom) genetic transformation according to Jones et al. (2002) with minor changes. Briefly, 7 days old cotyledons were used for the transformation. The explants were transferred to co-culture medium (MS + 100 μM AS + 0.1 mg/L KT + 0.2 mg/L 2,4-D), differentiation medium (MS + 2 mg/L ZR + 100 mg/L kanamycin + 400 mg/L augmentin+200 mg/L ticarcillin sodium), and root induction medium (1/2MS + 100 mg/L kanamycin) successively. The positive transgenic plants were identified by PCR. T1 generations of the transgenic lines were used for phenotype analysis. All plants were grown in plant culture room under controlled conditions (16 h light/8 h dark cycles, 25°C day/20°C night, and 60-70% relative humidity). In addition, the *A*. *tumefaciens* harbored *ZaMADS92* overexpression vector was transformed into *Arabidopsis thaliana* by floral dipping. The positive transgenic lines were screened with 100 mg/L kanamycin and confirmed by PCR. The T2 transgenic seedlings were used to observe the phenotypic characteristics.

### Phenotypical characterizations of *ZaMADS80*-OX and *ZaMADS92*-OX plants

Phenotypical characterization was performed on at least three independent transgenic tomato lines. For flower organ formation and development, the morphology of flower organs including sepals, petals and stamens was observed by a Cannon EOS 5D Mark digital camera (Tokyo, Japan) and a Leica S9i stereomicroscope (Leica Microsystems, Leica, Wetzlar, Germany), while the flowering time, the number of sepals and petals in each flower, as well as the quantity of flowers were documented. For fruit initiation and development, the fruit and sepal morphology were visualized, while the pistil size, the rate of fruit set and the number of seeds were counted. To observe the parthenocarpy feature of the *ZaMADS80*-OX lines, 7-day fruit was collected, fixed in Carnoy’s Fluid for 48 h at 4°C, sectioned, stained with Safranin O-fast green, and photographed by a standard bright field light microscope (Eclipse Ci-L, Nikon Instruments Inc., Tokyo, Japan) with a CCD camera. In addition, we observed the flowering time, the morphology of flower and fruit in wild-type and *ZaMADS92*-OX *Arabidopisis*.

## Results

### Identification and phylogenetic analysis of MADS-box genes in *Z. armatum*

The clean data of derived from RNA-Seq comprising 9 male floral buds (MFB), 24 fruit samples (F), and 6 male/female flowers (MFF) were assembled independently, and therefore generated three transcriptome databases, which contain 100238, 141105, and 77247 unigenes in MFB, F, and MFF libraries, respectively. Among them, 65, 136 and 86 candidate *MADS* genes were screened from the MFB, F and MFF transcriptome databases, respectively (unpublished data). After searching for the typic MADS-box domains and deletions of duplicate sequences, a total of 105 *MADS-box* genes were identified in *Z. armatum* and named *ZaMADS1* to *ZaMADS105* (**Supplemented Table S2**).

**Figure 1:**
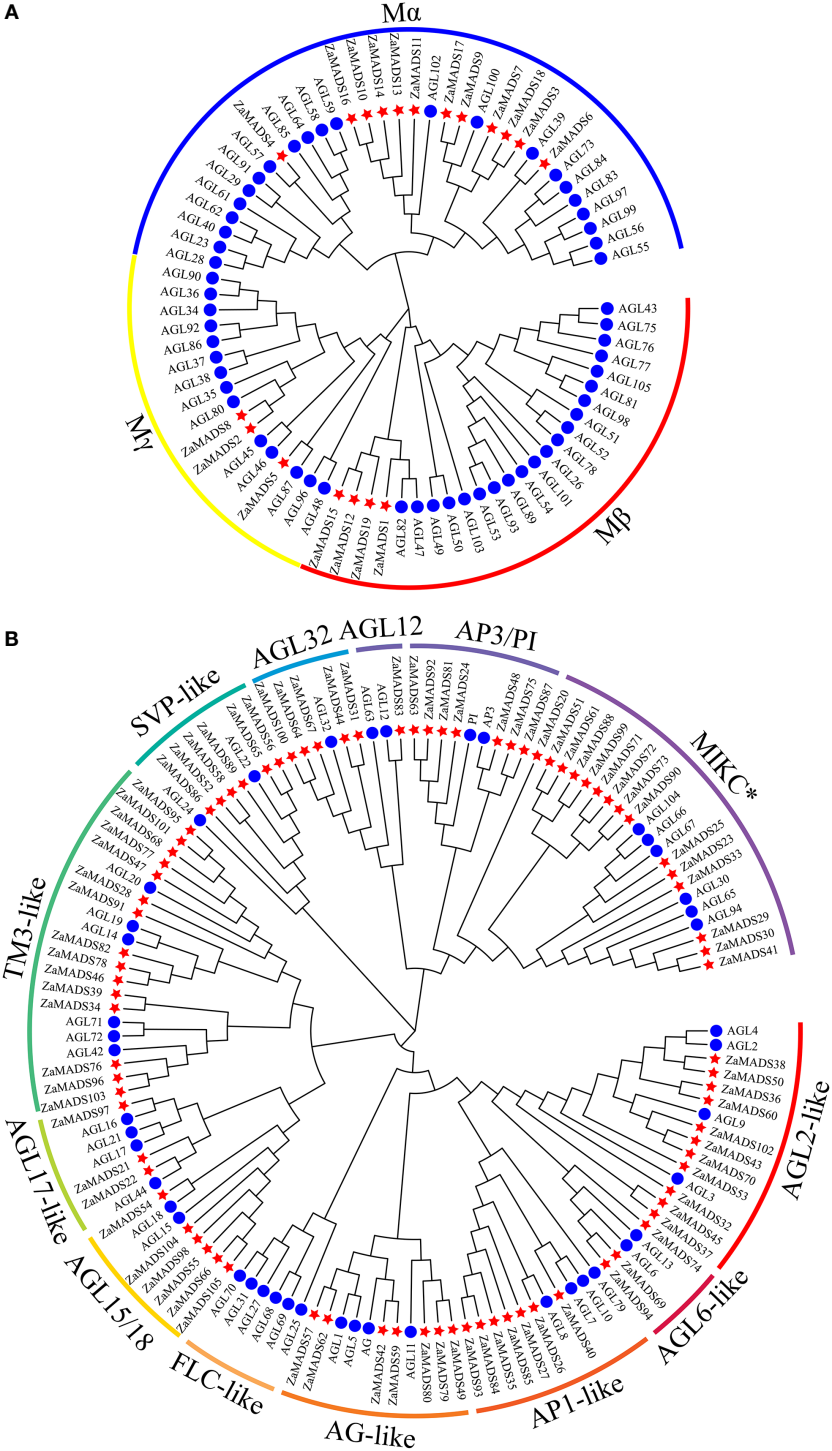
Phylogenetic analysis of the MADS-box proteins from *Z.armatum a*nd *Arabidopsis*. **(A)** A phylogenetic tree of 76 M-type MADS-box proteins from *Z.armatum* (19) and *Arabidopsis* (57) showing three subgroups including Mα, Mβ, and Mγ. **(B)** Phylogenetic analysis of MIKC type MADS-box proteins. 131 MIKC type MADS-box proteins from *Z.armatum* (86) and *Arabidopsis* (45) were classified into 12 MIKC^C^ subgroups and one MIKC* subgroup as marked in the Figure. The MADS-box genes are indicated by red stars and blue circles for *Z.armatum* and *Arabidopsis thaliana*, respectively. The sequences of M-type and MIKC type MADS-box proteins were listed in **Additional File 1** and **Additional File 2**, respectively.

A phylogenetic tree was constructed according to the conserved domain of the MADS proteins. 105 *MADS-box* genes were classified into two categories, M-type (type I) and MIKC MADS (type II), harboring 19 and 86 members, respectively (**Figure 1**). Independent phylogenetic trees of both types were constructed based on the amino acid sequences of MADSs from *Z. armatum* and *A. thaliana* (57 for M-type and 45 for MIKC) (**Additional File 1**, **File 2**). M-type *ZaMADS* genes were further divided into three subgroups: Mα, Mβ, and Mγ. The Mα subfamily was the largest, possessing 12 genes, while the Mβ and Mγ subgroups contained four and three members, respectively (**Figure 1A**). Based on structure divergence in the I domain, MIKC type ZaMADS proteins were classified into the MIKC^C^ and MIKC* subclades, including 71 and 15 genes, respectively. The *Z. armatum* MIKC^C^ proteins were further divided into 12 subfamilies in accordance with known classes of *Arabidopsis MADS-box* genes. Among them, the TM3-like branch contained the largest number (15) of ZaMADS MIKC^C^ -type genes. There were seven, six, five, eleven, seven, seven, and six MIKC^C^ members in AP3/PI, AGL15/18, AGL32, AGL2-, AP1-, AG-, and AGL32-like subfamilies, respectively. In contrast to *Arabidopsis*, the above eight MIKC^C^ subclusters were expanded by gene duplication. In addition, the remaining four subgroups consisted of only a few members, while FLC-like only existed in *Arabidopsis* (**Figure 1B**).

The conserved motifs from the 105 *ZaMADS* genes were identified using MEME online software (**Figures S2**, **S3**). The detailed motif information is available in **Figure S4**. The *MADS-box* genes in the same subgroup had similar motifs. For type I MADS, motif 1 corresponded to the typical MADS domain (SRF-like domain), which was found in the N-terminus of the seventeen M-type *ZaMADS* genes except for *ZaMADS2* and *ZaMADS18* (**Figure S2**). However, the NCBI CCD search showed that there was an SRF-domain in *ZaMADS18*. For type II MADS, Motifs 1 and 3 harbored the DNA binding site of MADS superfamily, while motifs 1 and 4 harbored a dimerization interface and putative phosphorylation site, which were observed in the majority of MIKC-type *ZaMADS* genes. Motifs 1 and 2 were regarded as the characteristic MADS domain, MEF2_like domain and K-box, respectively, which were found in the most of the MIKC^C^-type genes. However, no typic K-box was exhibited in MIKC^*^MADS. The complete K-Box was consisted of motifs 8, 2 and 5, mainly presented in AP1-like and AGL2-like subclades (**Figure S3**).

### Expression profiles of *MADS-box* genes during flower and fruit development in *Z. armatum*

**Figure 2:**
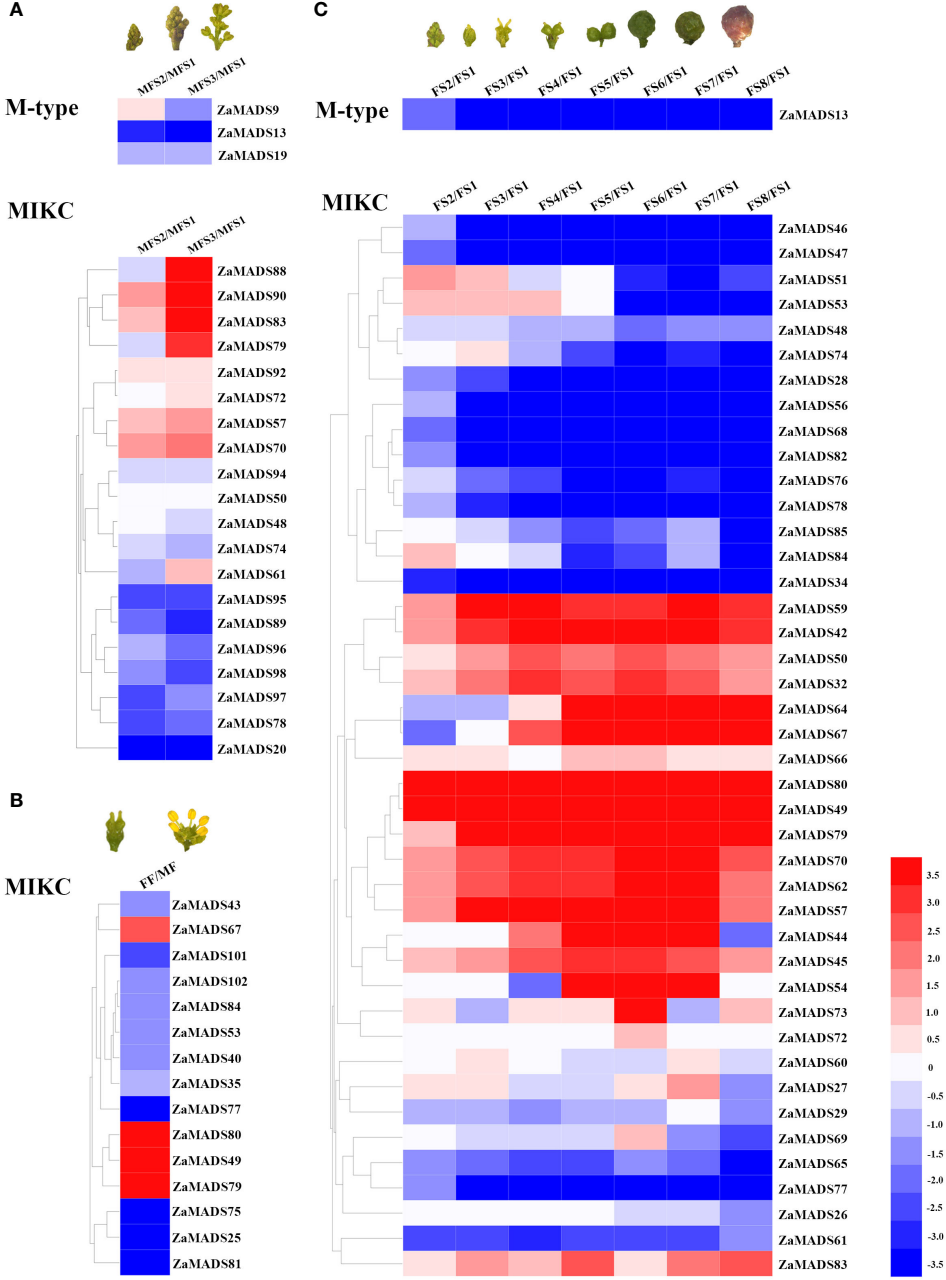
Transcriptomic mapping of *MADS-box* genes during flower and fruit development in *Z.armatum*. **(A)** Expression patterns of differentially expressed *MADS-box* genes during male flowers formation. **(B)** Expressions of differentially expressed *MADS-box* genes between male and female flowers. **(C)** Expressions of differentially expressed *MADS-box* genes during fruit initiation and development. Heatmap was illustrated by using Heml 1.0. Expression profile for each gene was shown in colored block. The expression changes were represented by Log 2 ratio. Red/blue color corresponds to up-/down-regulation of these genes, respectively.

To investigate the roles of *MADS-box* genes in floral organ determination and apomixis in dioecious *Z. armatum*, the transcript profiles of 105 *MADS-box* genes during male- and female-flowers at different developmental stages were obtained from RNA-seq data. There were ten *MADSs* presented exclusively and highly expressed in male floral organs, particularly three AP3/PI like genes (*ZaMADS92*, *ZaMADS81* and *ZaMADS75*), two SOC-like genes (*ZaMADS95* and *ZaMADS96*) and one SVP-like gene (*ZaMADS89*), while twenty-six *MADSs* were specifically restricted to female floral primordia and fruit, especially two STK/AGL11 like genes (*ZaMADS80* and *ZaMADS49*), one AG-like gene (*ZaMADS42*), two SOC-like genes (*ZaMADS47* and *ZaMADS68*), two AGL2-like genes (*ZaMADS53* and *ZaMADS32*) and two AGL32-like genes (*ZaMADS64* and *ZaMADS67*) (**Supplementary Table S3**; **Figure 2**), suggesting their potential roles in sexual differentiation.

In male plants, four *MADSs*, namely *ZaMADS92*, *ZaMADS57* (AGL1-like), *ZaMADS61*(AGL61-like) and *ZaMADS70* (SEP3-like), displayed remarkable upregulation with stamen initiation and development, whereas six genes, including *ZaMADS89*, *ZaMADS95*, *ZaMADS96*, *ZaMADS98* (AGL15-like), *ZaMADS74*(AGL6-like) and *ZaMADS78*(AGL19-like), showed dramatic declines in their transcripts (**Figure 2A**). Moreover, in female plants, several MADSs belonging to TM3-like subclades, including *ZaMADS28*, *ZaMADS34*, *ZaMADS46*, *ZaMADS47*, *ZaMADS68*, *ZaMADS76*, *ZaMADS78*, and *ZaMADS82*, as well as two SVP-like genes (*ZaMADS47* and *ZaMADS65*) exhibited relatively high transcript accumulation in pistil primordia and strongly downregulated following fruit set (FFS1 to FFS4) (**Figure 2C**), suggesting their potential roles in pistil development. Conversely, the transcript levels of four AG-like (*ZaMADS42*, *ZaMADS57*, *ZaMADS59* and *ZaMADS80*) and three AGL2-like (*ZaMADS70*, *ZaMADS50 ZaMADS53*) orthologous genes gradually increased with fruit set and expansion (FFS1 to FFS6), but significantly decreased response to fruit maturation, indicating that these genes might participate in apomixis and fruit growth. Additionally, we observed that *ZaMADS67*, an AGL32 homolog, was strictly restricted to enlarged and mature fruit (**Figure 2C**).

### Comprehensive interaction network of *Z. armatum* MADS-box proteins

**Figure 3:**
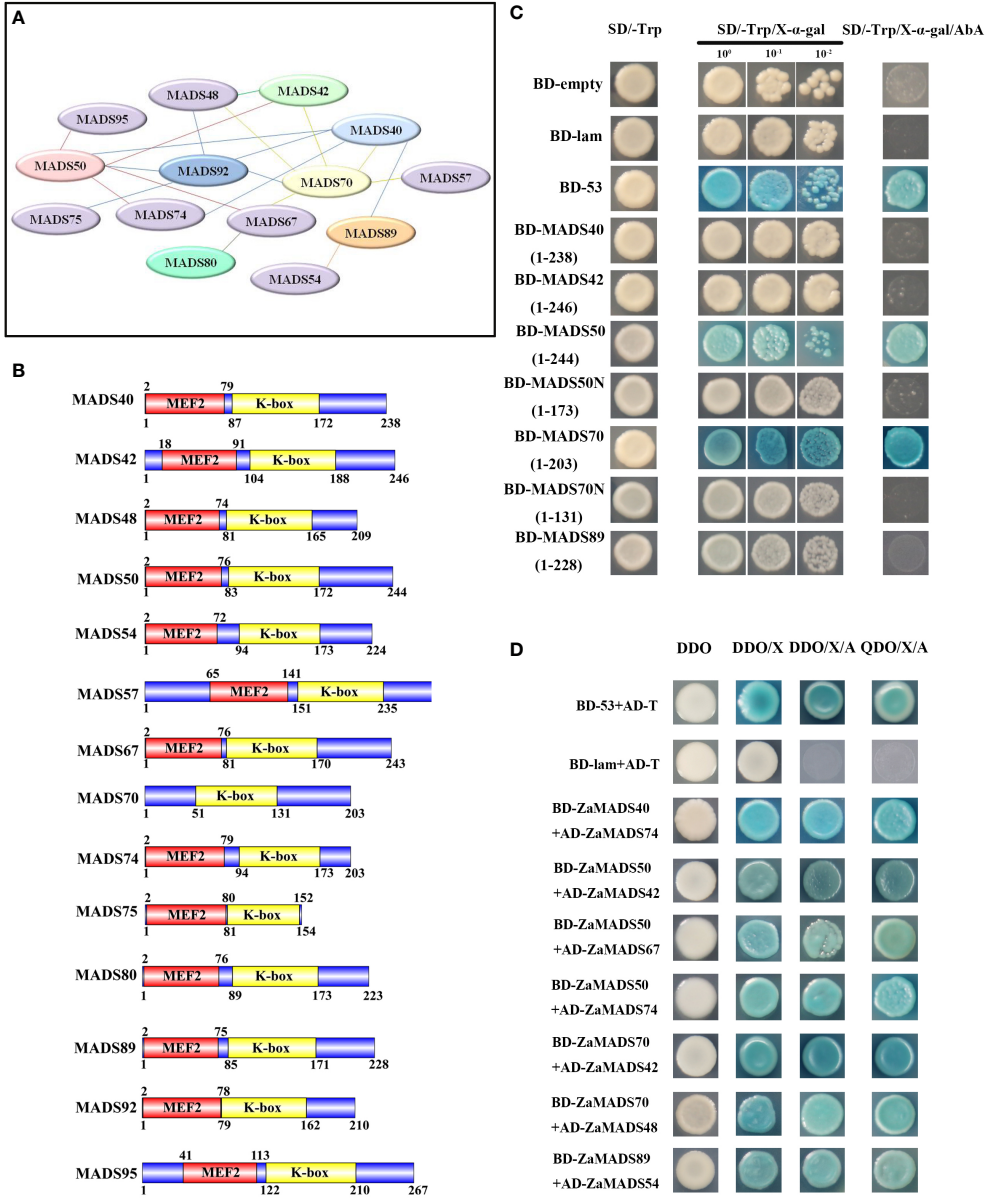
Protein-protein interaction analysis of MADS-box proteins. **(A)** The putative interaction network of MADSs predicted by String. **(B)** Analysis of conserved domain of MADS-box proteins. **(C)**Transcriptional activation activity assay of MADS-box proteins. Yeast cells were grown on the following selective medium: SD/-Trp, SD/-Trp+ x-α-Gal and SD/-Trp+x-α-Gal+AbA. Yeast cells harboring the pGBKT7-Lam vectors served as the negative control, and the pGBKT7-53 vectors served as the positive control. **(D)** Proteins-protein interaction analysis by yeast two-hybrid. AD-prey combined with BD-bait conferred yeast cells grew well on SD/−Leu/−Trp/-His/−Ade plates. DDO: SD/−Leu/−Trp medium, QDO: SD/−Ade/-His/-Leu/−Trp medium, DDO/X/A: DDO+X-α-Gal+AbA, QDO/X/A: QDO+X-α-Gal+AbA. AD-T + BD-53: positive control, AD-T + BD-Lam: negative control.

Fourteen MIKC^c^-type tomato MADS-domain proteins, candidates regulating sexual organ determination and apomixis differentiation according to their phylogenetic relationships with *Arabidopsis* and expression profiles during flower and fruit development, were subjected to the prediction of protein-protein interactions. A total of twenty interactions among these ZaMADSs were observed by using online prediction software (**Figure 3A**). Among them, ZaMADS50 (AGL2/SEP1 ortholog), ZaMADS70 (AGL9/SEP3 ortholog) and ZaMADS92 (PI ortholog) were hub proteins, showing a diverse interaction network. ZaMADS50 was predicted to interact with six proteins (ZaMADS40, ZaMADS42 ZaMADS67, ZaMADS74, ZaMADS92 and ZaMADS95), while ZaMADS70 was also hypothesized to interact with six proteins (ZaMADS40, ZaMADS42 ZaMADS48, ZaMADS57, ZaMADS67 and ZaMADS92). In addition, the interaction between ZaMADS92 and ZaMADS40/48/75 was screened out (**Figure 3A**).

To validate their interactions, 14 *MADS-box* genes were cloned into pGBKT7-BD and/or pGADT7-AD using a reciprocal mating scheme. Gene structure analysis showed that these ZaMADSs contain MEF and K-box domains, except for ZaMADS70 (**Figure 3B**). Transcriptional activity analysis showed that the yeast cells transformed with full-length *ZaMADS50* and *ZaMADS70* grew very well on the SD/-Trp and SD/-Trp/AbA medium and turned blue after supplementation with X- α -gal, indicating that these two MADS work as transcriptional activators. However, no transcriptional activation activities were observed in several MADSs including ZaMADS40, ZaMADS42, and ZaMADS89, as well as in C-terminal truncated ZaMADS50 (1-173) and ZaMADS70(1-131) (**Figure 3C**). A yeast two-hybrid assay revealed that ZaMADS50 strongly interacted with ZaMADS42, ZaMADS67, and ZaMADS74. Direct strong interactions were also present between ZaMADS40 (AP1 ortholog) and ZaMADS74, ZaMADS70 and ZaMADS42, as well as ZaMADS89 and ZaMADS54 (AGL15 ortholog) (**Figure 3D**). In addition, weak interactions were exhibited in several pairs such as ZaMADS40 and ZaMADS89, ZaMADS42 and ZaMADS48, ZaMADS50 and ZaMADS92, ZaMADS70 and ZaMADS67, indicated by the fact that the co-transformants showed X-α-galactosidase activity and grew well on DDO/X-α-gal/AbA, but could not grow on QDO/X-α-gal/AbA media (**Figure S5**).

### Overexpression of *ZaMADS80* results in dramatic modifications in vegetative and floral organ development

**Figure 4:**
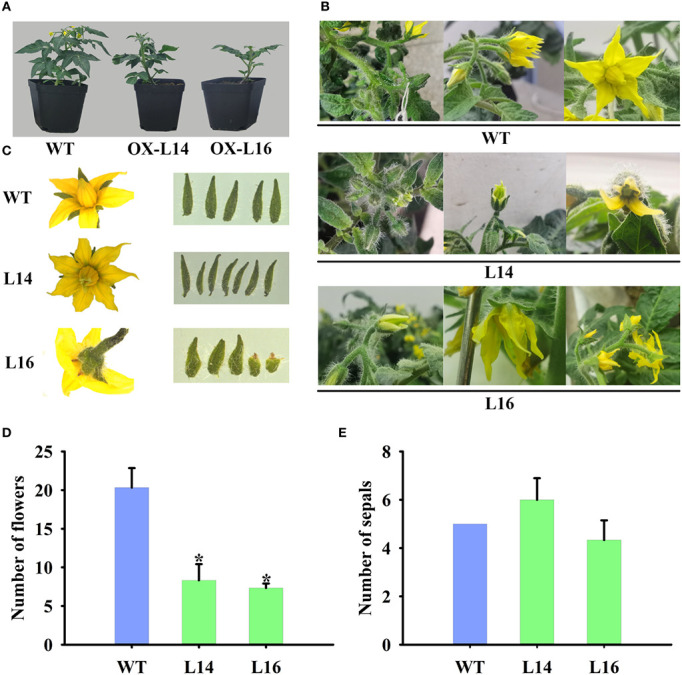
Dramatic modifications in tomato floral organ development triggered by *ZaMADS80* overexpression. **(A)** Vegetive growth and flowering in wild-type (WT) and *ZaMADS80*-OX lines (L14 and L16). **(B)** Observation of flowering processes of WT and *ZaMADS80*-OX tomatoes. **(C)** Morphology of floral organs, including sepals, petals and stamens, in WT and OX lines. **(D)** The total number of flowers in the first four inflorescences of WT and OX lines. **(E)** The number of sepals per flower in WT and OX lines. Error bars are the mean ± SE of at least three biological replicates. Asterisk indicates statistical significance (*P*<0.05) using Duncan’s new multiple range test. OX-L14 and OX-L16 represent two independent transgenic tomato plants.

Because of its fruit-specific expression in female plants, we investigated the function of ZaMADS80 in reproductive development. Overexpression of *ZaMADS80* in tomato resulted in shorter and thicker leaves with loss of serrated borders, severe growth retardation as well as delayed flowering. *ZaMADS80*-OX plants flowered 10-20 days later than wild-type (WT) plants (**Figure 4A**), likely due to deficiencies in the shoot apical meristem (**Figure S6A**). In WT tomatoes, sepals separated from petals and expanded, while petals wrapped stamens and pistils before anthesis, then turned yellow and expanded, the stamen still closed at anthesis. However, in *ZaMADS80*-OX lines, the sepals were tightly attached to petals in the majority of the flower buds, while petals were detached from stamens prior to anthesis. At anthesis, dehiscence in the apex of stamen was observed, and their stigmas protruded above the staminal cone (**Figure 4B**). Interestingly, the average number of floral organs in *ZaMADS80*-OX14 lines was higher than in the wild type due to the large number of flowers with six to seven sepals, petals, and stamens, compared with five sepals, petals, and stamens in WT flowers. However, OX16 lines exhibited deficient sepals or fusion of sepals, thus reducing the number of sepals, with only three to five pieces (**Figures 4B, C, E**). Moreover, compared to WT, the number of inflorescences and flowers (total number of flowers in first four inflorescences per plant) as well as the length of inflorescences and pedicels were extremely decreased in *ZaMADS80*-OX plants (**Figures 4A, B, D**).

### Overexpression of *ZaMADS80* induces parthenocarpy and improvement of fruit set

**Figure 5:**
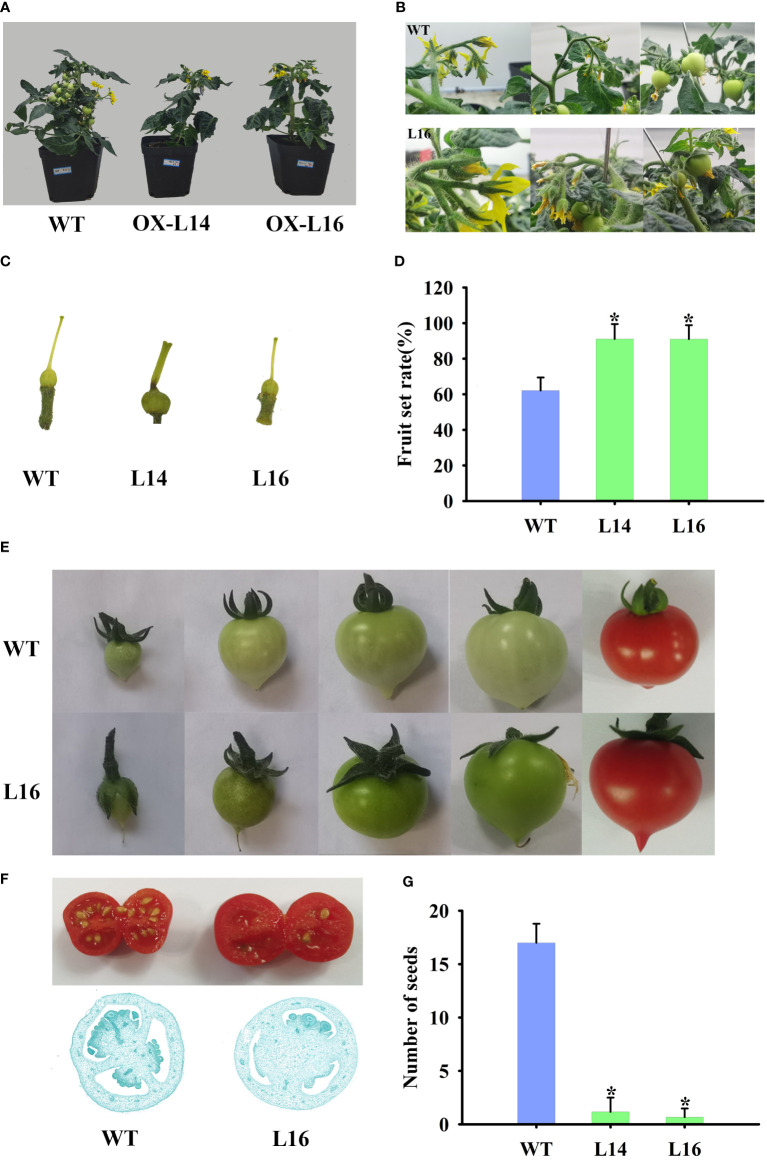
Overexpressing of *ZaMADS80* induces parthenocarpy. **(A)** Observation of fruit set and development in WT and *ZaMADS80*-OX tomatoes. **(B)** The processes of detailed fruit initiation and development in WT and OX-L16. **(C)** Morphology of pistils at the anthesis in WT and *ZaMADS80*-OX lines. **(D)** Fruit setting rate in WT and OX lines. **(E)** Fruits and sepals at different developmental stages in WT and OX-L16. **(F)** Transection of ripening fruit and histological observations of ovaries at 7 DPA in WT and OX-L16. **(G)** The number of seeds per fruit in WT and *ZaMADS80*-OX lines. Error bars are the mean ± SE of at least three biological replicates. Asterisk indicates statistical significance (*P*<0.05) using Duncan’s new multiple range test. OX-L14 and OX-L16 represent two independent transgenic tomato plants.

In accordance with the flowers, *ZaMADS80*-OX lines had fewer fruit per plant (**Figure 5A**). Under our growing conditions, we observed a relatively lower fruit set, with a rate of 60-70% from self-pollinated flowers of the first four inflorescences in WT plants. In contrast, *ZaMADS80*-overexpressing lines had a dramatic increase in fruit set, with a rate of 85-90% (**Figures 5B, D**). The ovaries in *ZaMADS80*-OX tomatoes began to expand at the flower-bud developmental stage (prior to anthesis), accompanied by thicker fruit stalks and enlarged sepals, facilitating the larger size of the ovaries. However, at this time, wild-type ovaries remained unchanged and underwent swelling just post anthesis and 72 h after pollination. Additionally, *ZaMADS80*-OX plants had thicker and shorter stigmas. We also observed that the sepals in several transgenic lines failed to scroll and enclosed the ovaries after fruit set, while the petals and stigmas remained attached to the fruit (**Figures 5C, E**). In contrast to WT, *ZaMADS80*-OX fruit displayed dark green color, larger size and higher weight (**Figure 5E**). Moreover, the average number of seeds in *ZaMADS80*-OX lines was extremely low compared to the wild type, with more than 15 per fruit. Approximately 80% of the fruit from transgenic plants was seedless (**Figures 5F, G**; **Figure S6B**). Histological observations of fruit sections also revealed remarkable declines in ovules numbers. In the inner part of the *ZaMADS80*-OX fruit, we observed a notable reduction in the locular space that was restricted to a thin ‘jelly’ surrounding the seeds (**Figure 5F**). The above results indicate the crucial roles of ZaMADS80 in parthenocarpy.

### ZaMADS80 functions *via* direct interaction with ZaMADS67 protein

**Figure 6:**
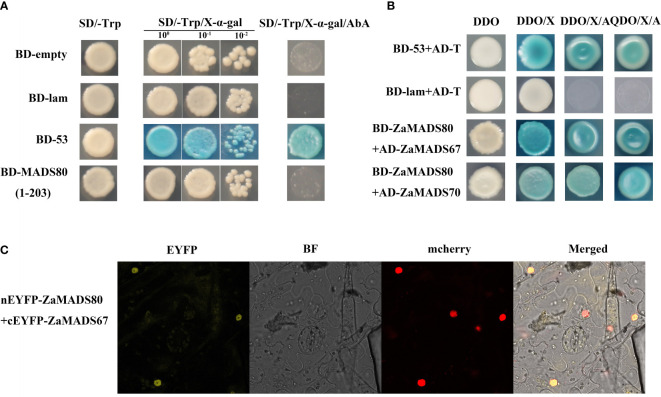
Protein-protein interaction between ZaMADS80 and ZaMADS67 determined by Y2H and BIFC. **(A)** Transcriptional activation activity assay of ZaMADS80 protein. **(B)** Protein interaction between ZaMADS80 and ZaMADS67 determined by Y2H. **(C)** Protein interaction between ZaMADS80 and ZaMADS67 determined by BIFC assay in *Nicotiana benthamiana* leaves. ZaMADS67 was fused with the C-terminal fragment of yellow fluorescence protein (EYFP) to form ZaMADS67-EYFP^C^. ZaMADS80 was fused with N-terminal fragment of EYFP to form ZaMADS80-EYFP^N^. The nucleus marker protein fused with red fluorescence protein (RFP).

Analysis of transcriptional activation in yeast revealed that the cells transformed with full-length *ZaMADS80* showed no X-α-galactosidase activity and could not grow on the SD/-Trp/AbA medium, suggesting that ZaMADS80 had no transcriptional activation activity and might function *via* interacting with other proteins (**Figure 6A**). In the predicted interaction network, ZaMADS80 was able to interact with ZaMADS67 (**Figure 3A**). Moreover, these two *MADSs* displayed fruit-specific expression and similar transcript profiles during fruit development (**Figure 2C**). Therefore, Y2H and BiFC assays were employed to validate their interactions. There was a strong interaction between ZaMADS80 and ZaMADS67, indicated by the fact that the co-transformants showed X-α -galactosidase activity and grew well on DDO/X-α-gal/AbA medium (**Figure 6B**). BiFC assay exhibited YFP fluorescence in the tobacco leaf co-infiltrated with ZaMADS80-EYFPN combined with ZaMADS67-EYFPC was clearly visualized, indicating the interaction of the two MADSs in the nucleus (**Figure 6C**). These results suggest that ZaMADS80 interacts with ZaMADS67 protein, which might coordinate to regulate floral development and apomixis in *Z. armatum*.

### *ZaMADS92* up-regulation has a limited effect on flower development

**Figure 7:**
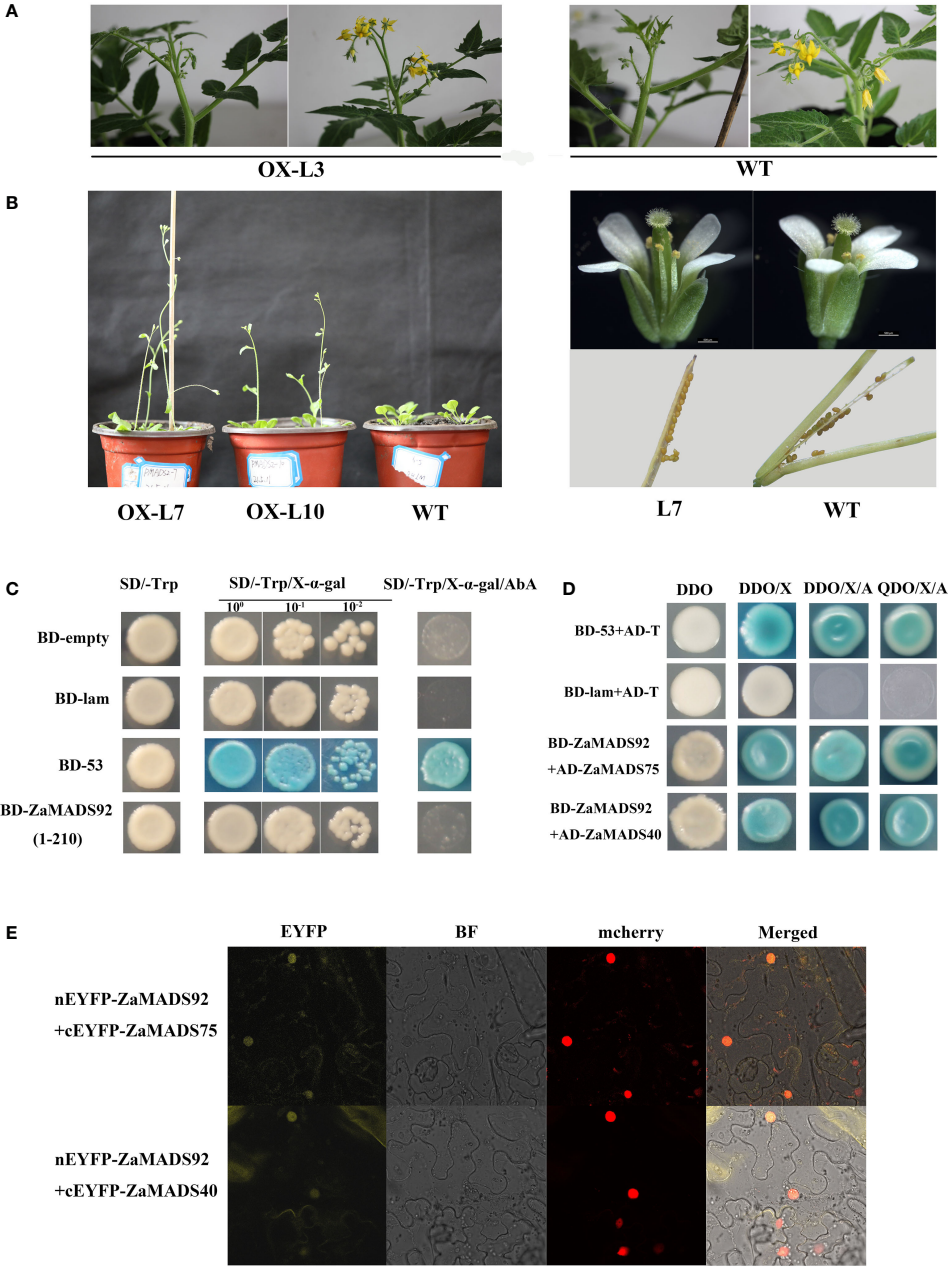
Functional characterization of ZaMADS92 gene involved in flowering. **(A)** Morphology of flowers in WT and *ZaMADS92-*overexpressing tomato plants. **(B)** Overexpressing *ZaMADS92* in *Arabidopsis* led to earlier flowering compared with WT. **(C)** Transcriptional activation activity assay of ZaMADS92 protein. **(D)** Protein-protein interaction analysis between ZaMADS92 and ZaMADS75/ZaMADS40 determined by Y2H. **(E)** Protein-protein interaction analysis between ZaMADS92 and ZaMADS75/ZaMADS40 determined by BIFC assay. ZaMADS75 and ZaMADS40 were fused with the C-terminal fragment of yellow fluorescence protein (EYFP) to form ZaMADS75-EYFP^C^ and ZaMADS40-EYFP^C^, respectively. ZaMADS80 was fused with N-terminal fragment of EYFP to form ZaMADS80-EYFP^N^. The nucleus marker protein fused with red fluorescence protein (RFP).

To explore the effects of male flower-specific genes on sex determination, we investigated the function of *ZaMADS92* using a transgenic strategy. Among the 4 overexpressing tomato lines, no obvious phenotypes were observed, either in the vegetative organs or in the flowers (**Figure 7A**). To validate this, more than ten individual OX lines in *Arabidopsis* were generated. All transgenic lines produced normal flowers and seeded fruit. Nonetheless, *ZaMADS92*-OX *Arabidopsis* exhibited earlier flowering than WT(**Figure 7B**). Transcriptional activation assay revealed that ZaMADS92 presented no transcriptional activation activity in yeast (**Figure 7C**). Y2H and BiFC assays demonstrated that ZaMADS92 strongly interacted with ZaMADS40 and ZaMADS75 (PMADS1 ortholog) *in vivo* (**Figures 7D, E**). These results indicate that ZaMADS92 might have limited functions in sexual differentiation of *Z. armatum*, but could control flowering time in coordination with ZaMADS40, ZaMADS75, and ZaMADS50.

## Discussion

### Involvement of MADS-box genes in sex determination and floral organ identity

Previous studies have revealed close correlations between MADS-box genes and sex determination (Di Stilio et al., 2005). In *Z. armatum*, due to the changes in cultivation patterns (cutting and rejuvenating fruit branches annually) and hormone application, the transition from female to male or hermaphrodite flowers occurred (**Figure S1A**). Moreover, only stamen or pistil primordia appeared in male or female flowers, respectively (**Figure S1B**). It is likely that MADS-box genes, especially class C, D, and E members, might participate in specifying sex organ identity and deletion of stamens or carpels by coordinated with their upstream regulators in *Z. armatum*. In this study, 105 MADS-box genes were identified in *Z. armatum*. Among them, 86 MIKC MADSs were observed (**Figure 1B**, **Supplementary Table S2**), which is higher than the numbers in *Arabidopsis* (Parenicova et al., 2003). The expansion of MIKC-type MADS-box genes might be due to the larger genome of *Z. armatum* cv Jiuyeqing (approximately 10 Gb estimated by k-mer analysis, unpublished data) as well as the requirements of floral organ identity in unisexual flowers of dioecious plants. Additionally, *Z. armatum* lacks the FLC ortholog (**Figure 1B**), the highly conserved and most important integrator of floral signals (Willmann and Poethig, 2011), which is consistent with watermelon and rice (Wang et al., 2019). We hypothesized that the gene regulatory network regulating flowering may be distinct in *Z. armatum*, which probably do not require vernalization for the flowering process, resulting in a relatively shorter transition from the juvenile-to-adult phase and vegetative-to-reproductive phase (flowering).

Class-B MADS-box genes PI/AP3 are responsible for the specification of male organs. In *Arabidopsis*, *ap3* and *pi* mutants displayed fewer carpels in the third whorl than the usual number of stamens, while in AP3/PI overexpression lines, there are many more stamens in the inner whorl than the usual number of carpels (Di Stilio et al., 2005). Two PI orthologs *ZaMADS92* and *ZaMADS81* as well as one AP3 ortholog *ZaMADS75* were only observed in male flowers and not detected in female inflorescences and fruit (**Figure 2**), which might be male sex-determining factors. Similarly, Liao et al. (2020) identified three MADS-box genes, *GbMADS1*, *GbMADS2*, and *GbMADS4*, the homologs of AP3/PI, exist only in male-specific contigs, implying that these genes might play an important role in sex-determining development in *Ginkgo*. The crucial role of PI in sex determination was also indicated by the fact that the sex determinant MeGI in persimmon, a repressor of the androecium, functions *via* regulating PI during early androecium development (Yang et al., 2019) and the feminizing factor ARR17 in *Populus*, controls dioecy by repressing PI expression (Müller et al., 2020; Leite Montalvão et al., 2022). Therefore, constitutive overexpression of PI/AP3 is expected to produce stamen-only flowers. However, no relevant phenotypes appeared in *ZaMADS92* overexpressing lines (**Figures 7A, B**). Further studies will focus on the functional significance of *ZaMADS81* and *ZaMADS75* in floral organ determination. We also observed an SVP ortholog, *ZaMADS89*, only presented in male flowers, indicating its involvement in sexual identity. In accordance with this, Urasaki et al. (2012) also reported that MADS-box gene *Cp2671* only exists on the Y and Y^h^ chromosomes in *Papaya*, which encodes a protein with 85% similarity to the SVP protein in *Arabidopsis*, a well-known transcriptional regulator for flowering time. Herein, we also observed several female-biased *MADS*, mainly AG, STK and SEP like genes (**Figure 2**), proved to associated with sex determination in various species (Zahn et al., 2005; Li and Liu, 2017), indicating their involvement in the determination of carpel identity. Previous studies have demonstrated that AG varies in the direction of sex bias across species. For instance, in grapevine, *VvMADS5*(AG homolog) exhibited female flower-limited expression, implying the involvement in gynoecium development (Feng et al., 2020), but in kiwifruit AG showed male-biased expression, indicating a role in androecium development (Yang et al., 2019). In this study, the transcripts of AG homologs were strictly limited to the female primordia and fruit, except for *ZaMADS57*, which was expressed in both male and female flowers (**Figure 2**). STK/AGL11 orthologues had strong sex-biased expression in female flowers (**Figure 2**), consistent with the carpel-specific expression of *LcMADS51* (LcSTK) in *Litchi* female flowers (Guan et al., 2021).

### Class-D MADS-box genes confer to apomixis in female plants of *Z. armatum*

The female plant of *Z. armatum* cv Jiuyeqing is a sporophytic apomictic species. Recently, several genes have been identified to regulate apomixis in many species, such as *RWP* in citrus, and *AGO9* and *MSI1* in *Arabidopsis* (Xu et al., 2022). In *Z*.*bungeanum*, Fei et al. (2021) reported that the MADS-box gene *ZaAGL11* was preferentially expressed at the apomictic nucellar embryo stage and its ectopic expression in *Arabidopsis* led to abnormal flower development and induced apomixis-like phenotypes. Additionally, previous studies have elucidated that silencing of *AGL6*, *TM8*, *TM29*, *TAP3*, *SlGLO1*, and *SlGLO2* or ectopic overexpression of *AGL11*, *TAG1* and *TAGL1* can induce parthenocarpy (Joldersma and Liu, 2018; Molesini et al., 2020). In view of the similar mechanistic basis for apomixis and parthenocarpy (Picarella and Mazzucato, 2019), we hypothesized that the female-specific expression of classes C, D, and E MADS might play crucial roles in apomixis of *Z. armatum* (**Figure 2**). Among them, three STK/AGL11 orthologues were identified and *ZaMADS80* showed female-limited and highly expressed in fruit (**Figures 2B, C**). Overexpressing *ZaMADS80* in tomato resulted in precocious fruit set and parthenocarpy (**Figures 5**, **6**). This is consistent with a previous report stating that *SlAGL11* up-regulation leads to seedless tomato fruit (Huang et al., 2017). Very recently, Huang et al. (2021) displayed that overexpression of *SlMBP3*, a *SlAGL11* homolog, also produces seedless fruit. Interestingly, they uncovered that suppression of *SlAGL11* had subtle effects on seed development, but silencing of *SlAGL11* and *SlMBP3* simultaneously resulted in parthenocarpy (Huang et al., 2021). This is in agreement with reports in *Petunia*, where simultaneous knockout of class D *FBP7*/*FBP11* led to seed defects and individual downregulation of *FBP7* or *FBP11* did not exhibit a seedless phenotype (Heijmans et al., 2012), suggesting a partial redundancy among class D genes.

In addition, a series of modifications in floral organs were observed in *ZaMADS80*-OX lines, which was partially consistent with those in *SlAGL11*- and *SlMBP3*-OX lines. For example, reduction in locular space, sepal swelling, and enclosing the inner organs were observed in *ZaMADS80*-, *SlAGL11*- and *SlMBP3*-OX tomatoes. However, *ZaMADS80*-OX lines produced larger fruit, but fruit size was reduced in *SlAGL11*- and *SlMBP3*-OX plants (**Figures 5**, **6**), indicating the different functions of AGL11 across species. In addition, *ZaMADS80*-OX plants shared phenotypes with tomato lines overexpressing class C *TAG1* and *TAGL1* genes, such as fused sepals, the precocious opening of the green immature anthers of petal and stamen, as well as the flowers failing to open until ovary swelling (Pnueli et al., 1994; Vrebalov et al., 2009), indicating that there might be an interaction between AGL11 and TAG1/TAGL1 to regulate parthenocarpy. Altogether, these results unraveled the critical roles of ZaMADS80 in *Z. armatum* apomixis. Further studies should characterize whether the other two AGL11 orthologs (*ZaMADS79* and *ZaMADS49*) are involved in apomixis.

### Protein-protein interactions of MADS proteins in *Z. armatum*

In *Z. armatum*, except for ZaMADS50(SEP1/AGL2-like) and ZaMADS70 (SEP3/AGL9-like), several MADS domain proteins representing the AP1, AP3/PI, SVP, SOC1, and AG subfamilies tested in this study, essential regulators for floral organ specification, exhibited no transcriptional activation activities (**Figure 3C**), indicating that these MADS transcription factors might function mediated by protein interactions *via* formation of heterodimers (Yan et al., 2016). This is in accordance with the viewpoint that SEP proteins function as bridges enabling floral quartets formation with the B- and C-class MADSs and provide transcriptional activation capacity for these complexes (Morel et al., 2019). Previous studies have revealed that interactions between MADS domain proteins are conserved among various plant species such as *Arabidopsis*, rice and tomato (de Folter et al., 2005; Leseberg et al., 2008). Moreover, the interaction partners share similar functions. AP1 has a dual function in floral meristem identity and floral organ determination (Mandel et al., 1992; Ferrandiz et al., 2000), which can interact with *AGL6* and *SVP-like* genes in rice and *Arabidopsis*, known to be involved in the timing of flowering, as well as with SEP1 and SEP3, masters governing floral organ identity (Fornara et al., 2004; de Folter et al., 2005; McCarthy et al., 2015). Herein, ZaMADS40 (a represented *AP1* ortholog) interacted with ZaMADS74 (AGL6-like) and ZaMADS89 (SVP-like), but not ZaMADS50 and ZaMADS70 (**Figure 3D**, **Figure S5**), which might contribute to the loss of sepals or petals in *Z. armatum* (only two whorls floral organ presented, tepal and stamen/pistil) (**Figure S1**). Hugouvieux et al. (2018) pointed out that SEP3 is the most promiscuous and active member, can interact with all floral organ identity MADS TFs, which can form heterotetramer with AG to activate key genes involved in meristem determinacy. In accordance with this, we observed that ZaMADS70 strongly interacted with ZaMADS42 and ZaMADS48(AP3-like) (**Figure 3D**). In *Aristolochia fimbriata*, SEP1 showed weak interactions with AG and AGL6 (Peréz‐Mesa et al., 2019), but in *Z. armatum*, strong interactions between these two partners were observed (**Figure 3D**), indicating divergence in interaction patterns across species. The PI protein pMADS2 was able to interact with AP3-like pMADS1 in *Petunia* (Vandenbussche et al., 2004), consistent with the observation of interaction between ZaMADS92(PI-like) and ZaMADS75(close homology to pMADS1), rather than ZaMADS48 (**Figure 7**, **Figure S5**). Moreover, the heterodimer formed by ZaMADS92 and ZaMADS40 might facilitate early flowering in *ZaMADS92*-OX lines (**Figure 7**). We demonstrated that ZaMADS80 (STK/AGL11) formed a heterodimer with ZaMADS67, a homolog of AGL32, its overexpression line displayed defects in female reproductive and produced few-seed fruit (Li et al., 2022). Additionally, ZaMADS70 was an interaction partner of ZaMADS80 (**Figure S7**). This result indicates that the carpel and ovule identities might be attributed to the interactions between ZaMADS80 and its counterparts, thus leading to apomixis of *Z. armatum*.

## Data availability statement

The original contributions presented in the study are publicly available. This data can be found here: NCBI, PRJNA901352 and PRJNA901699.

## Author contributions

Conceptualization, NT and ZCh; Methodology, NT, ZCa and XZ; Software, ZCa, PW and YL; Validation, NT, PW and ZCa; Formal analysis, ZCa, PW and QW; Investigation, XZ and QW; Resources, YH and SS; Data curation, XS and JL; Writing-original draft, NT; Writing-review and editing, ZCh; Visualization, JL; Supervision, JL; Funding acquisition, ZCh. All authors contributed to the article and approved the submitted version.

## Funding

This work was financially supported by the Projects for Innovative Research Groups of Chongqing Universities (No. CXQT21028), Chongqing talent program for Zexiong Chen, Scientific Technological Research Program of Chongqing Municipal Education Commission (No. KJZD-K201901303), and National Key Research and Development Project (No. 2019YFD1001205). The funders have no role in study design, data collection, data analysis, data interpretation, or writing of the manuscript.

## Conflict of interest

The authors declare that the research was conducted in the absence of any commercial or financial relationships that could be construed as a potential conflict of interest.

## Publisher’s note

All claims expressed in this article are solely those of the authors and do not necessarily represent those of their affiliated organizations, or those of the publisher, the editors and the reviewers. Any product that may be evaluated in this article, or claim that may be made by its manufacturer, is not guaranteed or endorsed by the publisher.

## Supplementary material

Supplementary Figure 1 Morphology of different types of flowers in *Zanthoxylum armatum.* **(A)** After pruning and hormone application, male and hermaphroditic flowers occur in female plants. **(B)** Anatomic structures of female flowers primordium. **(C)** Anatomic structures of male flower and hermaphroditic flower primordia. MF, male flower primordia; FF, female flower primordium; HF, hermaphroditic flower primordia; se, sepal; sp, stamen primordia; pp, pistil primordia.

Supplementary Figure 2 Distribution of conserved motifs in *Z.armatum* M-type MADS-box proteins. The conserved motifs of the ZaMADS proteins were identified by using MEME suite with zoops (zero or one occurrence per sequence) models of minimum and maximum width of 6 and 50 amino acid residues, respectively, and 10 maximum number of motifs. Different motifs are indicated by different colors.

Supplementary Figure 3 Distribution of conserved motifs in *Z.armatum* MIKC type MADS-box proteins identified by using MEME.

Supplementary Figure 4 The sequences of the conserved motifs in M-type and MIKC MADS-box proteins.

Supplementary Figure 5 Protein-protein interactions detected by yeast two-hybrid assay. **(A)** Yeast cells harboring bait and prey proteins exhibited X-α-galactosidase activity and grew well on DDO/X-a-gal/AbA, but not on QDO/X-a-gal/AbA media. **(B)** Yeast cells exhibited weak X-α-galactosidase activity on DDO without AbA application. **(C)** There were no interactions between bait and prey. DDO: SD/−Leu/−Trp medium, QDO: SD/−Ade/-His/-Leu/−Trp medium, DDO/X/A: DDO+X-α-Gal+AbA, QDO/X/A: QDO+X-α-Gal+AbA. AD-T + BD-53: positive control, AD-T + BD-Lam: negative control.

Supplementary Figure 6 The phenotype of the T2 generation of *ZaMADS80*-overexpressing tomato lines. **(A)** Transgenic tomatoes exhibited reduction in the differentiation of apical meristem. **(B)** Seedless fruit production in *ZaMADS80*-overexpressing lines.

Supplementary Figure 7 The putative interaction networks between ZaMADS80 and its counterparts.

Additional File 1 The amino acid sequences used in phylogenetic tree construction for M-type MADS-Box genes.

Additional File 2 The amino acid sequences used in phylogenetic tree construction for MIKC MADS-Box genes.

## References

[B1] Akagi T. Henry I. M. Tao R. Comai L. (2014). A y-chromosome–encoded small RNA acts as a sex determinant in persimmons. Science 346 (6209), 646–650. doi: 10.1126/science.1257225 25359977

[B2] de Folter S. Immink R. G. Kieffer M. Parenicova L. Henz S. R. Weigel D. . (2005). Comprehensive interaction map of the arabidopsis MADS box transcription factors. Plant Cell 17 (5), 1424–1433. doi: 10.1105/tpc.105.031831 15805477 PMC1091765

[B3] Di Stilio V. S. Kramer E. M. Baum D. A. (2005). Floral MADS box genes and homeotic gender dimorphism in thalictrum dioicum (Ranunculaceae)–a new model for the study of dioecy. Plant J. 41 (5), 755–766. doi: 10.1111/j.1365-313X.2005.02336.x 15703062

[B4] Fei X. Shi Q. Qi Y. Wang S. Lei Y. Hu H. . (2021). ZbAGL11, a class d MADS-box transcription factor of zanthoxylum bungeanum, is involved in sporophytic apomixis. Horticult. Res. 8, 23. doi: 10.1038/s41438-020-00459-x PMC7848008 33518706

[B5] Feng G. Sanderson B. J. Keefover-Ring K. Liu J. Ma T. Yin T. . (2020). Pathways to sex determination in plants: how many roads lead to Rome? Curr. Opin. Plant Biol. 54, 61–68. doi: 10.1016/j.pbi.2020.01.004 32106015

[B6] Ferrandiz C. Gu Q. Martienssen R. Yanofsky M. F. (2000). Redundant regulation of meristem identity and plant architecture by FRUITFULL, APETALA1 and CAULIFLOWER. Development 127, 725–734. doi: 10.1242/dev.127.4.725 10648231

[B7] Fornara F. Parenicová L. Falasca G. Pelucchi N. Masiero S. Ciannamea S. . (2004). Functional characterization of OsMADS18, a member of the AP1/SQUA subfamily of MADS box genes. Plant Physiol. 135 (4), 2207–2219. doi: 10.1104/pp.104.045039 15299121 PMC520791

[B8] Guan H. Wang H. Huang J. Liu M. Chen T. Shan X. . (2021). Genome-wide identification and expression analysis of MADS-box family genes in litchi (Litchi chinensis sonn.) and their involvement in floral sex determination. Plants 10 (10), 2142. doi: 10.3390/plants10102142 34685951 PMC8540616

[B9] Guerin J. Rossel J. B. Robert S. Tsuchiya T. Koltunow A. (2000). A DEFICIENS homologue is down-regulated during apomictic initiation in ovules of hieracium. Planta 210 (6), 914–920. doi: 10.1007/s004250050697 10872222

[B10] Heijmans K. Ament K. Rijpkema A. S. Zethof J. Wolters-Arts M. Gerats T. . (2012). Redefining c and d in the petunia ABC. Plant Cell 24 (6), 2305–2317. doi: 10.1105/tpc.112.097030 22706285 PMC3406901

[B11] Huang B. Hu G. Wang K. Frasse P. Maza E. Djari A. . (2021). Interaction of two MADS-box genes leads to growth phenotype divergence of all-flesh type of tomatoes. Nat. Commun. 12 (1), 1–14. doi: 10.1038/s41467-021-27117-7 34824241 PMC8616914

[B12] Huang B. Routaboul J. M. Liu M. Deng W. Maza E. Mila I. . (2017). Overexpression of the class d MADS-box gene sl-AGL11 impacts fleshy tissue differentiation and structure in tomato fruits. J. Exp. Bot. 68 (17), 4869–4884. doi: 10.1093/jxb/erx303 28992179

[B13] Hugouvieux V. Silva C. S. Jourdain A. Stigliani A. Charras Q. Conn V. . (2018). Tetramerization of MADS family transcription factors SEPALLATA3 and AGAMOUS is required for floral meristem determinacy in arabidopsis. Nucleic Acids Res. 46 (10), 4966–4977. doi: 10.1093/nar/gky205 29562355 PMC6007258

[B14] Ito T. Wellmer F. Yu H. Das P. Ito N. Alves-Ferreira M. . (2004). The homeotic protein AGAMOUS controls microsporogenesis by regulation of SPOROCYTELESS. Nature 430 (6997), 356–360. doi: 10.1038/nature02733 15254538

[B15] Joldersma D. Liu Z. (2018). The making of virgin fruit: the molecular and genetic basis of parthenocarpy. J. Exp. Bot. 69 (5), 955–962. doi: 10.1093/jxb/erx446 29325151 PMC6018997

[B16] Jones B. Frasse P. Olmos E. Zegzouti H. Li Z. G. Latché A. . (2002). Down-regulation of DR12, an auxin-response-factor homolog, in the tomato results in a pleiotropic phenotype including dark green and blotchy ripening fruit. Plant J. 32 (4), 603–613. doi: 10.1046/j.1365-313X.2002.01450.x 12445130

[B17] Leite Montalvão A. P. Kersten B. Kim G. Fladung M. Müller N. A. (2022). ARR17 controls dioecy in populus by repressing b-class MADS-box gene expression. Philos. Trans. R. Soc. B 377 (1850), 20210217. doi: 10.1098/rstb.2021.0217 PMC8935312 35306887

[B18] Leseberg C. H. Eissler C. L. Wang X. Johns M. A. Duvall M. R. Mao L. (2008). Interaction study of MADS-domain proteins in tomato. J. Exp. Bot. 59 (8), 2253–2265. doi: 10.1093/jxb/ern094 18487636

[B19] Liao Q. Du R. Gou J. Guo L. Shen H. Liu H. . (2020). The genomic architecture of the sex-determining region and sex-related metabolic variation in ginkgo biloba. Plant J. 104 (5), 1399–1409. doi: 10.1111/tpj.15009 33015884

[B20] Li F. Jia Y. Zhou S. Chen X. Xie Q. Hu Z. . (2022). SlMBP22 overexpression in tomato affects flower morphology and fruit development. J. Plant Physiol. 272, 153687. doi: 10.1016/j.jplph.2022.153687 35378388

[B21] Li Q. Liu B. (2017). Genetic regulation of maize flower development and sex determination. Planta 245 (1), 1–14. doi: 10.1007/s00425-016-2607-2 27770199

[B22] Liu X. He X. Liu Z. Wu P. Tang N. Chen Z. . (2022). Transcriptome mining of genes in zanthoxylum armatum revealed ZaMYB86 as a negative regulator of prickly development. Genomics 114 (3), 110374. doi: 10.1016/j.ygeno.2022.110374 35489616

[B23] Love M. I. Huber W. Anders S. (2014). Moderated estimation of fold change and dispersion for RNA-seq data with DESeq2. Genome Biol. 15 (12), 1–21. doi: 10.1186/s13059-014-0550-8 PMC4302049 25516281

[B24] Mandel M. A. Gustafson-Brown C. Savidge B. Yanofsky M. F. (1992). Molecular characterization of the arabidopsis floral homeotic gene APETALA1. Nature 360, 273–277. doi: 10.1038/360273a0 1359429

[B25] McCarthy E. W. Mohamed A. Litt A. (2015). Functional divergence of APETALA1 and FRUITFULL is due to changes in both regulation and coding sequence. Front. Plant Sci. 6, 1076. doi: 10.3389/fpls.2015.01076 26697035 PMC4667048

[B26] Molesini B. Dusi V. Pennisi F. Pandolfini T. (2020). How hormones and mads-box transcription factors are involved in controlling fruit set and parthenocarpy in tomato. Genes 11 (12), 1441. doi: 10.3390/genes11121441 33265980 PMC7760363

[B27] Morel P. Chambrier P. Boltz V. Chamot S. Rozier F. Rodrigues Bento S. . (2019). Divergent functional diversification patterns in the SEP/AGL6/AP1 MADS-box transcription factor superclade. Plant Cell 31 (12), 3033–3056. doi: 10.1105/tpc.19.00162 31591161 PMC6925017

[B28] Müller N. A. Kersten B. Leite Montalvão A. P. Mähler N. Bernhardsson C. Bräutigam K. . (2020). A single gene underlies the dynamic evolution of poplar sex determination. Nat. Plants 6 (6), 630–637. doi: 10.1038/s41477-020-0672-9 32483326

[B29] Parenicova L. de Folter S. Kieffer M. Horner D. S. Favalli C. Busscher J. . (2003). Molecular and phylogenetic analyses of the complete MADS-box transcription factor family in arabidopsis: new openings to the MADS world. Plant Cell 15 (7), 1538–1551. doi: 10.1105/tpc.011544 12837945 PMC165399

[B30] Peréz-Mesa P. Suárez-Baron H. Ambrose B. A. González F. Pabón-Mora N. (2019). Floral MADS-box protein interactions in the early diverging angiosperm aristolochia fimbriata Cham.(Aristolochiaceae: Piperales). Evol. Dev. 21 (2), 96–110. doi: 10.1111/ede.12282 30734997

[B31] Picarella M. E. Mazzucato A. (2019). The occurrence of seedlessness in higher plants; insights on roles and mechanisms of parthenocarpy. Front. Plant Sci. 9, 1997. doi: 10.3389/fpls.2018.01997 30713546 PMC6345683

[B32] Pnueli L. Hareven D. Rounsley S. D. Yanofsky M. F. Lifschitz E. (1994). Isolation of the tomato AGAMOUS gene TAG1 and analysis of its homeotic role in transgenic plants. Plant Cell 6 (2), 163–173. doi: 10.1105/tpc.6.2.163 7908549 PMC160424

[B33] Subbaraya U. Rajendran S. Simeon S. Suthanthiram B. Somasundram S. M. (2020). Unravelling the regulatory network of transcription factors in parthenocarpy. Sci. Hortic. 261, 108920. doi: 10.1016/j.scienta.2019.108920

[B34] Theißen G. Melzer R. Rümpler F. (2016). MADS-domain transcription factors and the floral quartet model of flower development: Linking plant development and evolution. Development 143 (18), 3259–3271. doi: 10.1242/dev.134080 27624831

[B35] Urasaki N. Tarora K. Shudo A. Ueno H. Tamaki M. Miyagi N. . (2012). Digital transcriptome analysis of putative sex-determination genes in papaya (Carica papaya). PloS One 7 (7), e40904. doi: 10.1371/journal.pone.0040904 22815863 PMC3397944

[B36] Vandenbussche M. Zethof J. Royaert S. Weterings K. Gerats T. (2004). The duplicated b-class heterodimer model: whorl-specific effects and complex genetic interactions in petunia hybrida flower development. Plant Cell 16 (3), 741–754. doi: 10.1105/tpc.019166 14973163 PMC385285

[B37] Vrebalov J. Pan I. L. Arroyo A. J. M. McQuinn R. Chung M. Poole M. . (2009). Fleshy fruit expansion and ripening are regulated by the tomato SHATTERPROOF gene TAGL1. Plant Cell 21 (10), 3041–3062. doi: 10.1105/tpc.109.066936 19880793 PMC2782289

[B38] Wang M. Tong S. Ma T. Xi Z. Liu J. (2021). Chromosome-level genome assembly of sichuan pepper provides insights into apomixis, drought tolerance, and alkaloid biosynthesis. Mol. Ecol. Resour. 21 (7), 2533–2545. doi: 10.1111/1755-0998.13449 34145765

[B39] Wang P. Wang S. Chen Y. Xu X. Guang X. Zhang Y. (2019). Genome-wide analysis of the MADS-box gene family in watermelon. Comput. Biol. Chem. 80, 341–350. doi: 10.1016/j.compbiolchem.2019.04.013 31082717

[B40] Willmann M. R. Poethig R. S. (2011). The effect of the floral repressor FLC on the timing and progression of vegetative phase change in arabidopsis. Development 138 (4), 677–685. doi: 10.1242/dev.057448 21228003 PMC3026413

[B41] Wuest S. E. O’Maoileidigh D. S. Rae L. Kwasniewska K. Raganelli A. Hanczaryk K. . (2012). Molecular basis for the specification of floral organs by APETALA3 and PISTILLATA. Proc. Natl. Acad. Sci. 109 (33), 13452–13457. doi: 10.1073/pnas.1207075109 22847437 PMC3421202

[B42] Xu Y. Jia H. Tan C. Wu X. Deng X. Xu Q. (2022). Apomixis: genetic basis and controlling genes. Horticult. Res 9, uhac150. doi: 10.1093/hr/uhac150 PMC9437720 36072837

[B43] Yan W. Chen D. Kaufmann K. (2016). Molecular mechanisms of floral organ specification by MADS domain proteins. Curr. Opin. Plant Biol. 29, 154–162. doi: 10.1016/j.pbi.2015.12.004 26802807

[B44] Yang H. W. Akagi T. Kawakatsu T. Tao R. (2019). Gene networks orchestrated by me GI: a single-factor mechanism underlying sex determination in persimmon. Plant J. 98 (1), 97–111. doi: 10.1111/tpj.14202 30556936 PMC6850717

[B45] Zahn L. M. Kong H. Leebens-Mack J. H. Kim S. Soltis P. S. Landherr L. L. . (2005). The evolution of the SEPALLATA subfamily of MADS-box genes: a preangiosperm origin with multiple duplications throughout angiosperm history. Genetics 169 (4), 2209–2223. doi: 10.1534/genetics.104.037770 15687268 PMC1449606

[B46] Zhang X. Tang N. Liu X. Ye J. Zhang J. Chen Z. . (2020). Comparative transcriptome analysis identified differentially expressed genes between male and female flowers of zanthoxylum armatum var. novemfolius. Agronomy 10 (2), 283. doi: 10.3390/agronomy10020283

