# Supplementary figures and images for: Genome-wide identification, interaction of the MADS-box proteins in Zanthoxylum armatum and functional characterization of ZaMADS80 in floral development
Source: Front Plant Sci. 2022 Nov 25;13:1038828. doi: 10.3389/fpls.2022.1038828 (PMC9732391; doi:10.3389/fpls.2022.1038828)

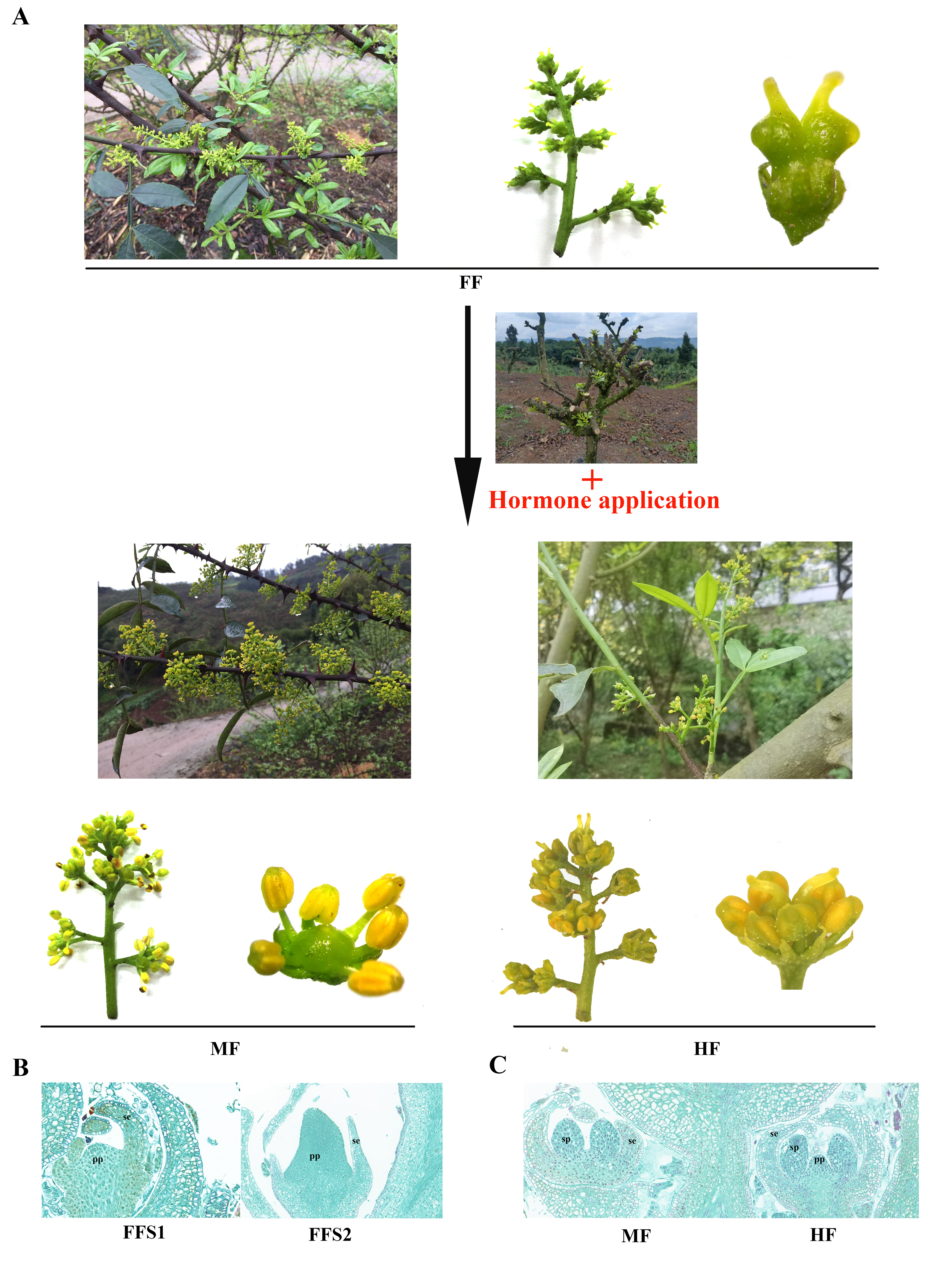

Supplement: Supplementary Figure 1 — Morphology of different types of flowers in Zanthoxylum armatum. (A) After pruning and hormone application, male and hermaphroditic flowers occur in female plants. (B) Anatomic structures of female flowers primordium. (C) Anatomic structures of male flower and hermaphroditic flower primordia. MF, male flower primordia; FF, female flower primordium; HF, hermaphroditic flower primordia; se, sepal; sp, stamen primordia; pp, pistil primordia. [file Image_1.jpeg]

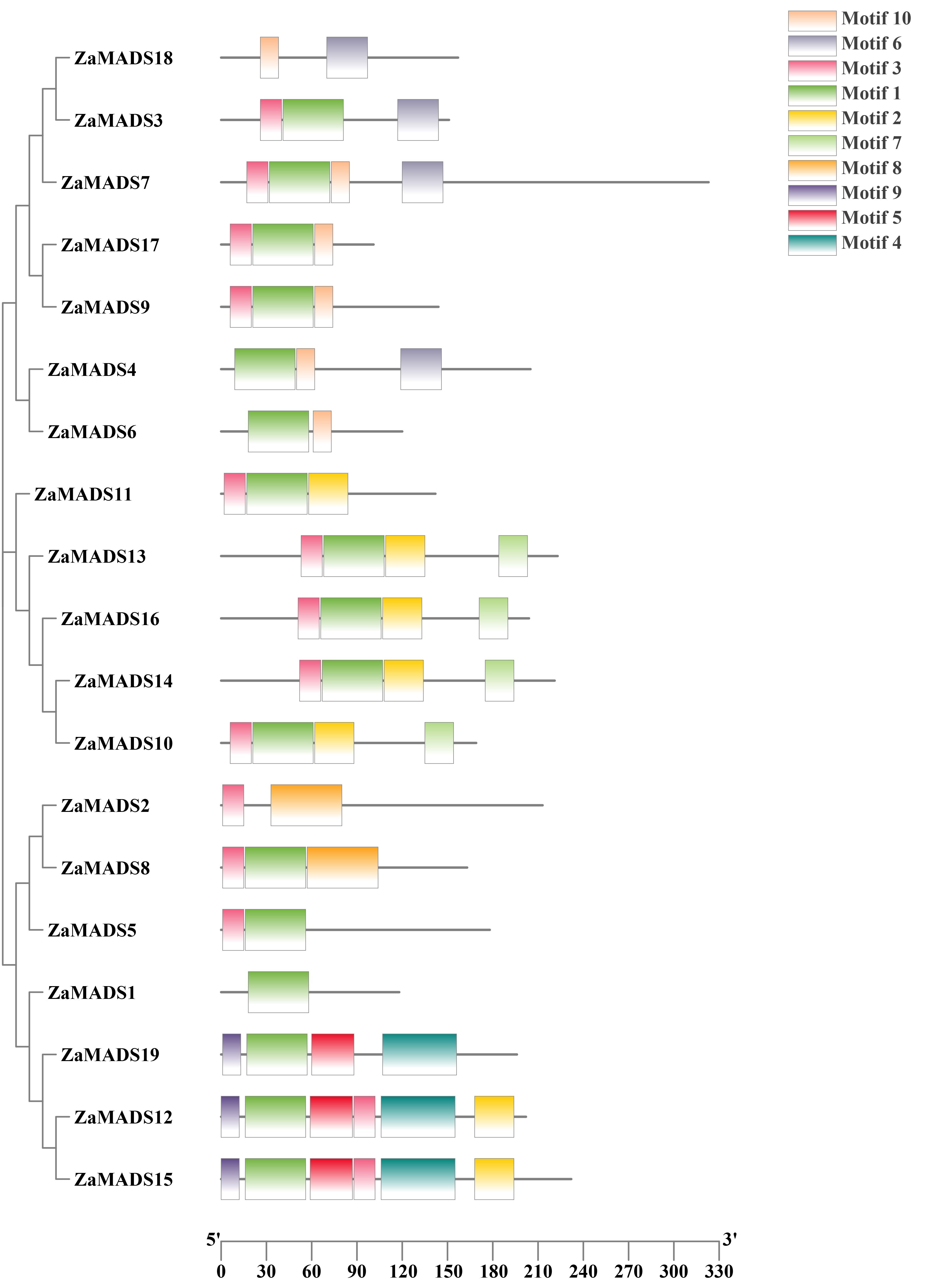

Supplement: Supplementary Figure 2 — Distribution of conserved motifs in Z.armatum M-type MADS-box proteins. The conserved motifs of the ZaMADS proteins were identified by using MEME suite with zoops (zero or one occurrence per sequence) models of minimum and maximum width of 6 and 50 amino acid residues, respectively, and 10 maximum number of motifs. Different motifs are indicated by different colors. [file Image_2.jpeg]

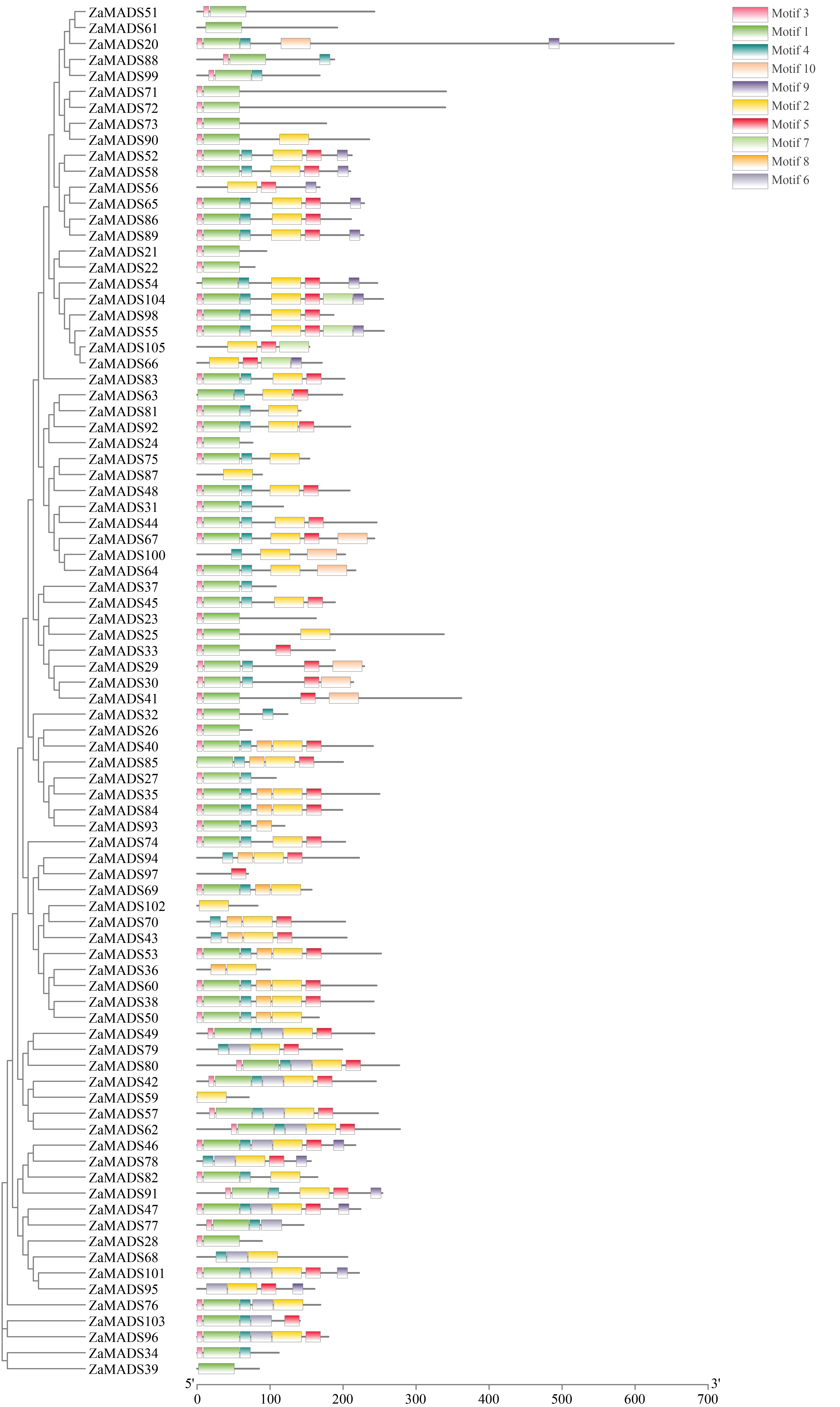

Supplement: Supplementary Figure 3 — Distribution of conserved motifs in Z.armatum MIKC type MADS-box proteins identified by using MEME. [file Image_3.jpeg]

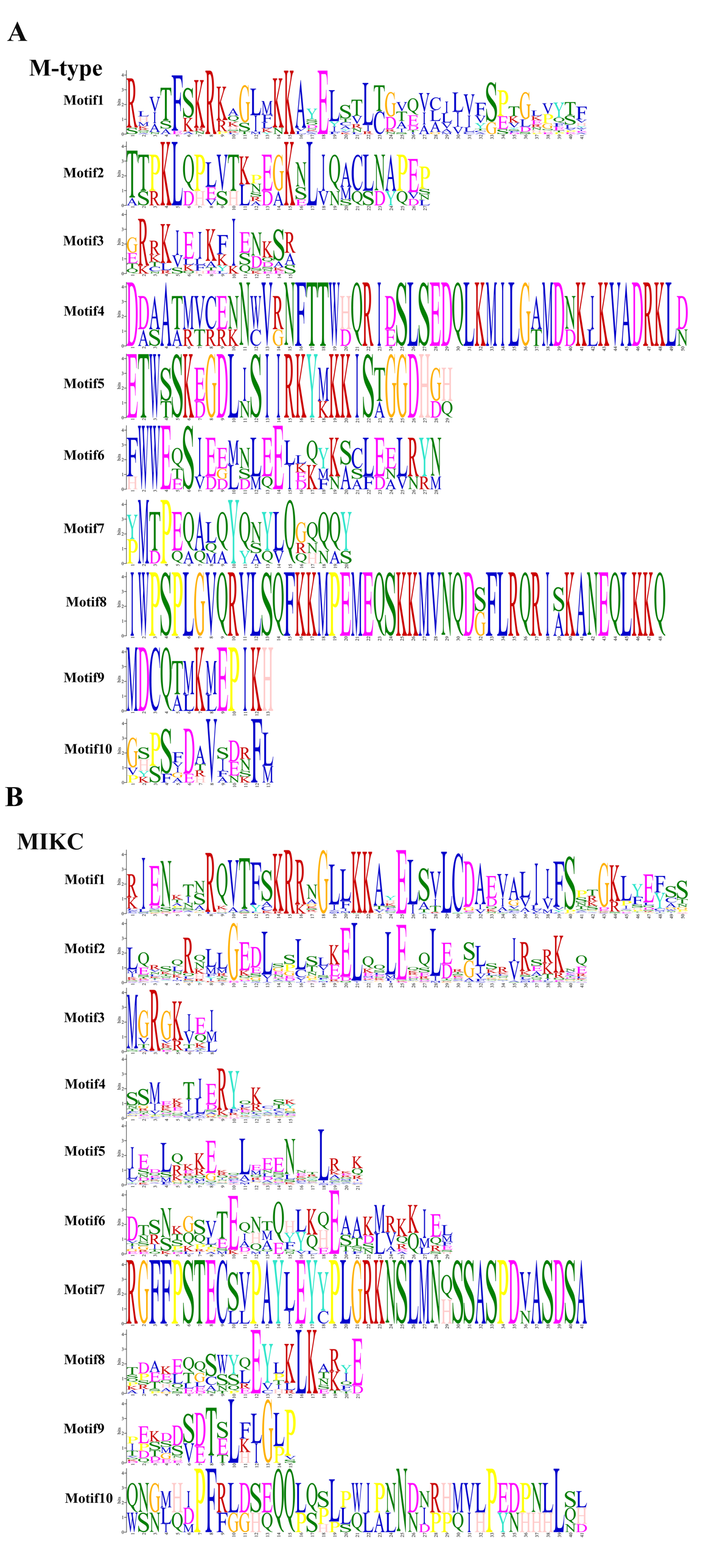

Supplement: Supplementary Figure 4 — The sequences of the conserved motifs in M-type and MIKC MADS-box proteins. [file Image_4.jpeg]

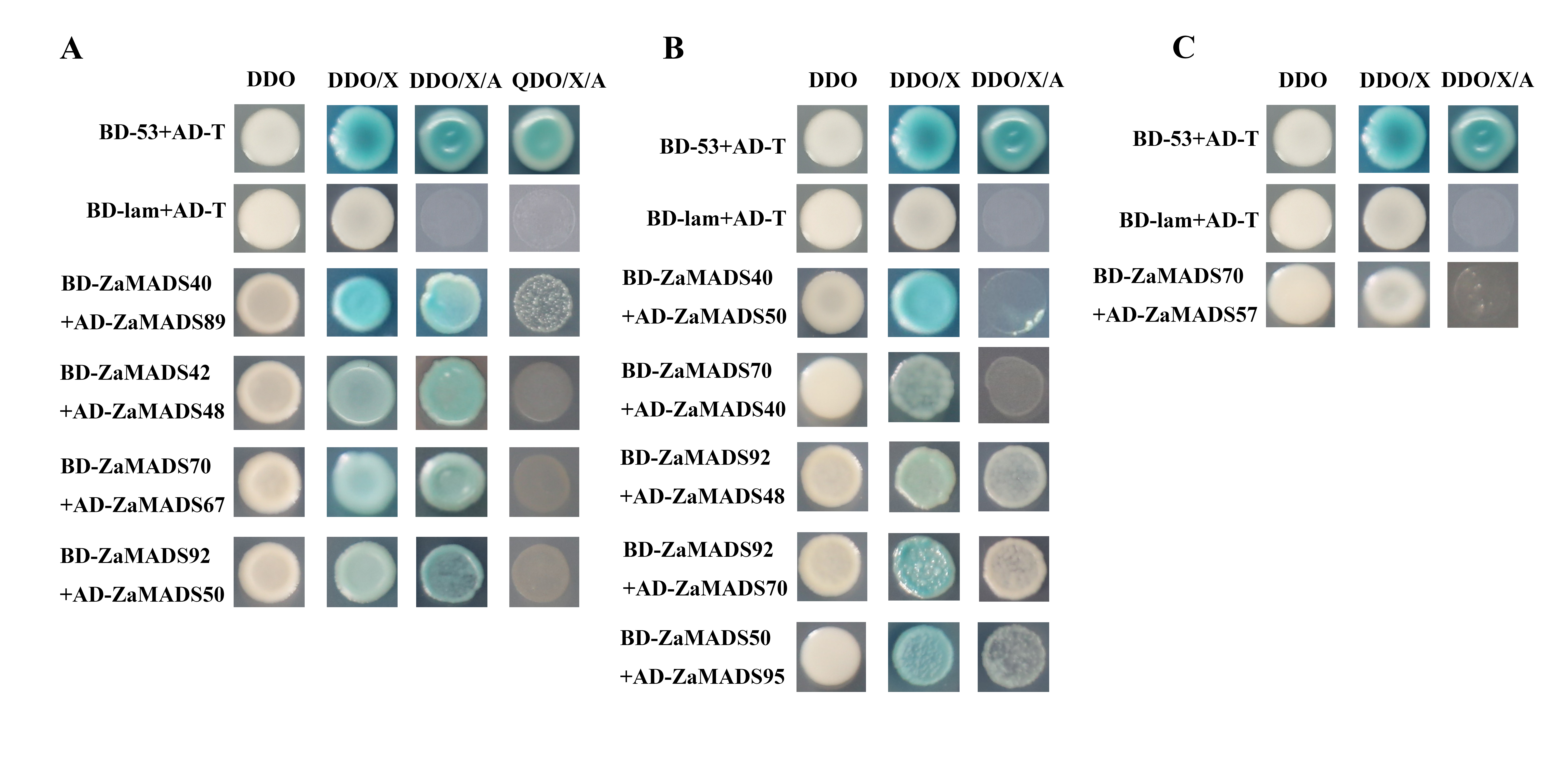

Supplement: Supplementary Figure 5 — Protein-protein interactions detected by yeast two-hybrid assay. (A) Yeast cells harboring bait and prey proteins exhibited X-α-galactosidase activity and grew well on DDO/X-a-gal/AbA, but not on QDO/X-a-gal/AbA media. (B) Yeast cells exhibited weak X-α-galactosidase activity on DDO without AbA application. (C) There were no interactions between bait and prey. DDO: SD/−Leu/−Trp medium, QDO: SD/−Ade/-His/-Leu/−Trp medium, DDO/X/A: DDO+X-α-Gal+AbA, QDO/X/A: QDO+X-α-Gal+AbA. AD-T + BD-53: positive control, AD-T + BD-Lam: negative control. [file Image_5.jpeg]

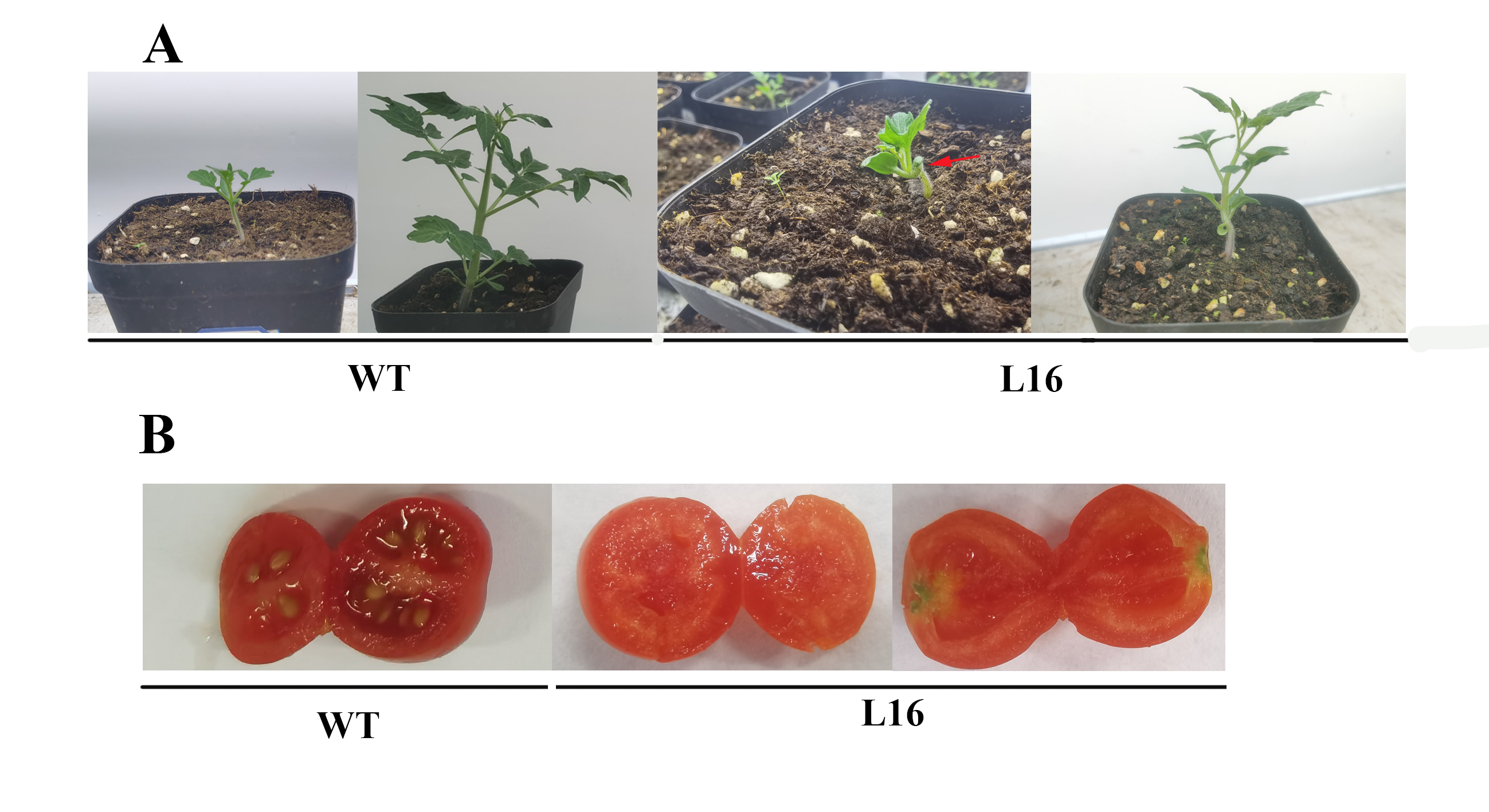

Supplement: Supplementary Figure 6 — The phenotype of the T2 generation of ZaMADS80-overexpressing tomato lines. (A) Transgenic tomatoes exhibited reduction in the differentiation of apical meristem. (B) Seedless fruit production in ZaMADS80-overexpressing lines. [file Image_6.jpeg]

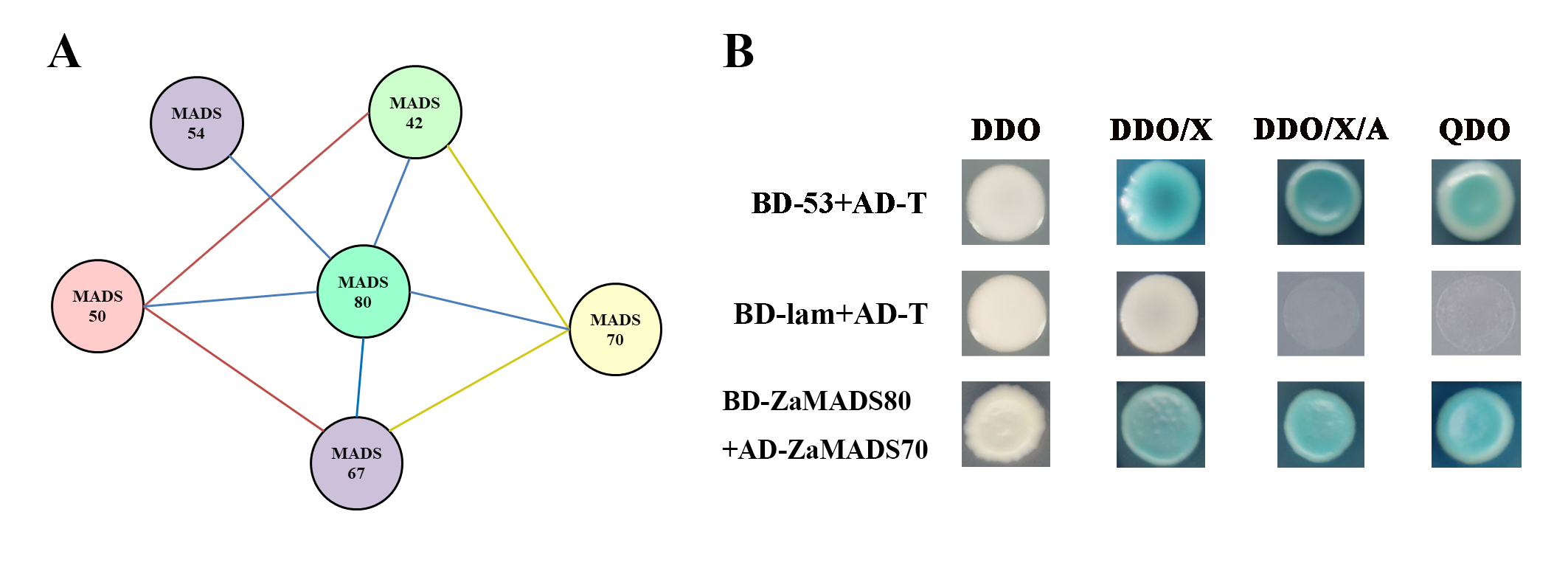

Supplement: Supplementary Figure 7 — The putative interaction networks between ZaMADS80 and its counterparts. [file Image_7.jpeg]
