# Supplementary material for: Genome-wide identification, interaction of the MADS-box proteins in Zanthoxylum armatum and functional characterization of ZaMADS80 in floral development
Source: Front Plant Sci. 2022 Nov 25;13:1038828. doi: 10.3389/fpls.2022.1038828 (PMC9732391; doi:10.3389/fpls.2022.1038828)
Supplement: Supplementary file 8 [file Table_1.docx]

**Supplementary Table S1. The primers used in this study**

| **EXP Type** | **Gene name** | **Forward/reverse primer (5’-3’)** |
| --- | --- | --- |
| Overexpression | ZaMADS80 | ATACTTCCAACTAGTGCGGCCGCATGGGGAGGGGAAAGATTGAG |
|  |  | ATGGTCATCCCGGGACCTGCAGGTTACCCAAGATGAAGTGTCTTTTTGTC |
|  | ZaMADS92 | ATACTTCCAACTAGTGCGGCCGCATGGGAAGAGGCAAGATTGAGATA |
|  |  | ATGGTCATCCCGGGACCTGCAGGCTAGTATGTTCTAGCCTGTTTGTTG |
| **EXP Type** | **Gene name** | **Forward/reverse primer (5’-3’)** |
| Yeast two-hybrid | AD-ZaMADS40 | TGGAGGCCAGTGAATTCATGGGAAGAGGTAGGGTTCAG |
|  |  | TCGAGCTCGATGGATCCCTAGCGAAGCATCCGTGTGA |
|  | AD-ZaMADS42 | TGGAGGCCAGTGAATTCATGGCGTTTCCAAATGAATTGGC |
|  |  | TCGAGCTCGATGGATCCTTAAACAAACCGAAGGGCCATCT |
|  | AD-ZaMADS48 | TGGAGGCCAGTGAATTCATGGCTAGAGGAAAGATCCAGAT |
|  |  | TCGAGCTCGATGGATCCTTAGTTAGGCTGCAGGCGTAT |
|  | AD-ZaMADS50 | TGGAGGCCAGTGAATTCATGGGGAGAGGGAGAGTTGA |
|  |  | TCGAGCTCGATGGATCCTCAAAGCATCCATCCTGGGATG |
|  | AD-ZaMADS54 | TGGAGGCCAGTGAATTCATGGGGAGGGGCAAAATAGAG |
|  |  | TCGAGCTCGATGGATCCTTACCCTAAATGCAAGGAAGTATCAG |
|  | AD-ZaMADS57 | TGGAGGCCAGTGAATTCATGAACGAAGAACTCACTTCCATTTTC |
|  |  | TCGAGCTCGATGGATCCTCAAACGAGCTGGAGAGGGA |
|  | AD-ZaMADS67 | TGGAGGCCAGTGAATTCATGGGACGGGGGAAGATAG |
|  |  | TCGAGCTCGATGGATCCCTAGATATTGGGATCCTGAAGG |
|  | AD-ZaMADS70 | TGGAGGCCAGTGAATTCATGAAGCCAATTGAGACGTTTTTTAG |
|  |  | TCGAGCTCGATGGATCCTCACACTTGGTCCTGAGTGAC |
|  | AD-ZaMADS74 | TGGAGGCCAGTGAATTCATGGGAAGGGGAAGAGTGGA |
|  |  | TCGAGCTCGATGGATCCTCAAAGCATCCACTCTGGAATG |
|  | AD-ZaMADS75 | TGGAGGCCAGTGAATTCATGCAAGAGACATACAGGAAATTGAAG |
|  |  | TCGAGCTCGATGGATCCAGATTCATTTAGATCGAGTTGAGATATTG |
|  | AD-ZaMADS89 | TGGAGGCCAGTGAATTCATGGCAAGAGAGAAGATCAAGATC |
|  |  | TCGAGCTCGATGGATCCTCAGTTGGAGAAGGGAAGCC |
|  | AD-ZaMADS95 | TGGAGGCCAGTGAATTCATGCTCTTGCAATATCATAAAAACCAC |
|  |  | TCGAGCTCGATGGATCCTCAGTTATGCAGCGACATTGG |
|  | BD-ZaMADS40 | CATGGAGGCCGAATTCATGGGAAGAGGTAGGGTTCAG |
|  |  | GCAGGTCGACGGATCCCTAGCGAAGCATCCGTGTGA |
|  | BD-ZaMADS42 | CATGGAGGCCGAATTCATGGCGTTTCCAAATGAATTGGC |
|  |  | GCAGGTCGACGGATCCTTAAACAAACCGAAGGGCCATCT |
|  | BD-ZaMADS50 | CATGGAGGCCGAATTCATGGGGAGAGGGAGAGTTGA |
|  |  | GCAGGTCGACGGATCCTCAAAGCATCCATCCTGGGATG |
|  | BD-ZaMADS50R2 | GCAGGTCGACGGATCCATTTCATCCAGCTTTATTGTCAAAGCT |
|  | BD-ZaMADS70 | CATGGAGGCCGAATTCATGAAGCCAATTGAGACGTTTTTTAG |
|  |  | GCAGGTCGACGGATCCTCACACTTGGTCCTGAGTGAC |
|  | BD-ZaMADS70R2 | GCAGGTCGACGGATCCTCAACCTTTGTCTGAGGTTCTTATTT |
|  | BD-ZaMADS80 | CATGGAGGCCGAATTCATGGGGAGGGGAAAGATTGAG |
|  |  | GCAGGTCGACGGATCCTTACCCAAGATGAAGTGTCTTTTTGTC |
|  | BD-ZaMADS89 | CATGGAGGCCGAATTCATGGCAAGAGAGAAGATCAAGATC |
|  |  | GCAGGTCGACGGATCCTCAGTTGGAGAAGGGAAGCC |
|  | BD-ZaMADS92 | CATGGAGGCCGAATTCATGGGAAGAGGCAAGATTGAGAT |
|  |  | GCAGGTCGACGGATCCCTAAATAGGCTGCATGTTGAAAGTG |
| **EXP Type** | **Gene name** | **Forward/reverse primer (5’-3’)** |
| BIFC | ZaMADS40 | AGTGGTCTCTGTCCAGTCCTATGGAAAAGATACTGGAACGCTAC |
|  |  | GGTCTCAGCAGACCACAAGTGCGAAGCATCCGTGTGAG |
|  | ZaMADS67 | AGTGGTCTCTGTCCAGTCCTATGGGACGGGGGAAGATAG |
|  |  | GGTCTCAGCAGACCACAAGTGATATTGGGATCCTGAAGGTTG |
|  | ZaMADS75 | AGTGGTCTCTGTCCAGTCCTATGCAAGAGACATACAGGAAATTGAAG |
|  |  | GGTCTCAGCAGACCACAAGTAGATTCATTTAGATCGAGTTGAGATATTGA |
|  | ZaMADS80 | AGTGGTCTCTGTCCAGTCCTATGGGGAGGGGAAAGATTGAG |
|  |  | GGTCTCAGCAGACCACAAGTCCCAAGATGAAGTGTCTTTTTGTC |
