# Supplementary material for: Genome-wide identification, interaction of the MADS-box proteins in Zanthoxylum armatum and functional characterization of ZaMADS80 in floral development
Source: Front Plant Sci. 2022 Nov 25;13:1038828. doi: 10.3389/fpls.2022.1038828 (PMC9732391; doi:10.3389/fpls.2022.1038828)
Supplement: Supplementary file 9 [file Table_2.docx]

**Supplementary Table S2. Sequence features of MADS-box genes in *Z. armatum.***

| No. | Gene name | Length(aa) | CDS | Group | *Arabidopsis* |
| --- | --- | --- | --- | --- | --- |
| 1 | ZaMADS1 | 118 | partial | Mβ | AGL105 |
| 2 | ZaMADS2 | 213 | complete | Mγ | AGL80 |
| 3 | ZaMADS3 | 151 | partial | Mα | AGL28 |
| 4 | ZaMADS4 | 205 | complete | Mα | AGL61 |
| 5 | ZaMADS5 | 178 | partial | Mγ | AGL87 |
| 6 | ZaMADS6 | 120 | partial | Mα | AGL55 |
| 7 | ZaMADS7 | 323 | complete | Mα | AGL29 |
| 8 | ZaMADS8 | 163 | complete | Mγ | AGL80 |
| 9 | ZaMADS9 | 144 | partial | Mα | AGL29 |
| 10 | ZaMADS10 | 169 | partial | Mα | AGL69 |
| 11 | ZaMADS11 | 142 | partial | Mα | AGL69 |
| 12 | ZaMADS12 | 202 | complete | Mβ | AGL82 |
| 13 | ZaMADS13 | 223 | complete | Mα | AGL69 |
| 14 | ZaMADS14 | 221 | complete | Mα | AGL69 |
| 15 | ZaMADS15 | 232 | complete | Mβ | AGL82 |
| 16 | ZaMADS16 | 204 | complete | Mα | AGL69 |
| 17 | ZaMADS17 | 101 | complete | Mα | AGL64 |
| 18 | ZaMADS18 | 157 | complete | Mα | AGL61 |
| 19 | ZaMADS19 | 196 | complete | Mβ | AGL82 |
| 20 | ZaMADS20 | 653 | complete | MIKC | AGL15 |
| 21 | ZaMADS21 | 95 | complete | AGL17-like | AGL16 |
| 22 | ZaMADS22 | 79 | partial | AGL17-like | AGL44 |
| 23 | ZaMADS23 | 164 | partial | MIKC | AGL104 |
| 24 | ZaMADS24 | 76 | complete | AP3/PI | AGL3 |
| 25 | ZaMADS25 | 338 | complete | MIKC | AGL104 |
| 26 | ZaMADS26 | 75 | complete | AP1-like | AGL8 |
| 27 | ZaMADS27 | 108 | complete | AP1-like | AGL7 |
| 28 | ZaMADS28 | 89 | complete | TM3-like | AGL14 |
| 29 | ZaMADS29 | 229 | complete | MIKC | AGL30 |
| 30 | ZaMADS30 | 214 | complete | MIKC | AGL30 |
| 31 | ZaMADS31 | 118 | partial | AGL32 | AGL32 |
| 32 | ZaMADS32 | 124 | complete | AGL2-like | AGL9 |
| 33 | ZaMADS33 | 189 | complete | MIKC | AGL94 |
| 34 | ZaMADS34 | 112 | complete | TM3-like | AGL42 |
| 35 | ZaMADS35 | 250 | complete | AP1-like | AGL7 |
| 36 | ZaMADS36 | 100 | partial | AGL2-like | AGL2 |
| 37 | ZaMADS37 | 108 | partial | AGL2-like | AGL3 |
| 38 | ZaMADS38 | 242 | complete | AGL2-like | AGL4 |
| 39 | ZaMADS39 | 85 | complete | TM3-like | AGL42 |
| 40 | ZaMADS40 | 238 | complete | AP1-like | AGL7 |
| 41 | ZaMADS41 | 362 | complete | MIKC | AGL30 |
| 42 | ZaMADS42 | 246 | complete | AG-like | AG |
| 43 | ZaMADS43 | 205 | complete | AGL2-like | AGL9 |
| 44 | ZaMADS44 | 246 | complete | AGL32 | AGL32 |
| 45 | ZaMADS45 | 189 | partial | AGL2-like | AGL7 |
| 46 | ZaMADS46 | 217 | complete | TM3-like | AGL19 |
| 47 | ZaMADS47 | 224 | complete | TM3-like | AGL20 |
| 48 | ZaMADS48 | 209 | partial | AP3/PI | AP3 |
| 49 | ZaMADS49 | 243 | complete | AG-like | AGL11 |
| 50 | ZaMADS50 | 244 | complete | AGL2-like | AGL2 |
| 51 | ZaMADS51 | 243 | partial | MIKC | AGL61 |
| 52 | ZaMADS52 | 212 | complete | SVP-like | AGL22 |
| 53 | ZaMADS53 | 252 | complete | AGL2-like | AGL2 |
| 54 | ZaMADS54 | 224 | complete | AGL15/AGL18 | AGL18 |
| 55 | ZaMADS55 | 256 | complete | AGL15/AGL18 | AGL15 |
| 56 | ZaMADS56 | 168 | complete | SVP-like | AGL22 |
| 57 | ZaMADS57 | 294 | complete | AG-like | AGL1 |
| 58 | ZaMADS58 | 210 | complete | SVP-like | AGL22 |
| 59 | ZaMADS59 | 71 | complete | AG-like | AGL5 |
| 60 | ZaMADS60 | 246 | complete | AGL2-like | AGL4 |
| 61 | ZaMADS61 | 192 | complete | MIKC | AGL61 |
| 62 | ZaMADS62 | 278 | complete | AG-like | AGL1 |
| 63 | ZaMADS63 | 199 | partial | AP3/PI | PI |
| 64 | ZaMADS64 | 217 | complete | AGL32 | AGL32 |
| 65 | ZaMADS65 | 229 | complete | SVP-like | AGL22 |
| 66 | ZaMADS66 | 171 | complete | AGL15/AGL18 | AGL15 |
| 67 | ZaMADS67 | 243 | complete | AGL32 | AGL32 |
| 68 | ZaMADS68 | 206 | partial | TM3-like | AGL20 |
| 69 | ZaMADS69 | 157 | complete | AGL6-like | AGL6 |
| 70 | ZaMADS70 | 203 | complete | AGL2-like | AGL9 |
| 71 | ZaMADS71 | 341 | complete | MIKC | AGL48 |
| 72 | ZaMADS72 | 340 | complete | MIKC | AGL80 |
| 73 | ZaMADS73 | 177 | complete | MIKC | AGL80 |
| 74 | ZaMADS74 | 203 | complete | AGL6-like | AGL6 |
| 75 | ZaMADS75 | 154 | complete | AP3/PI | AP3 |
| 76 | ZaMADS76 | 169 | complete | TM3-like | AGL42 |
| 77 | ZaMADS77 | 146 | partial | TM3-like | AGL20 |
| 78 | ZaMADS78 | 156 | partial | TM3-like | AGL19 |
| 79 | ZaMADS79 | 199 | partial | AG-like | AGL11 |
| 80 | ZaMADS80 | 223 | complete | AG-like | AGL11 |
| 81 | ZaMADS81 | 142 | complete | AP3/PI | PI |
| 82 | ZaMADS82 | 165 | partial | TM3-like | AGL19 |
| 83 | ZaMADS83 | 202 | complete | AGL12 | AGL12 |
| 84 | ZaMADS84 | 199 | partial | AP1-like | AGL8 |
| 85 | ZaMADS85 | 200 | partial | AP1-like | AGL7 |
| 86 | ZaMADS86 | 211 | partial | SVP-like | AGL22 |
| 87 | ZaMADS87 | 89 | complete | AP3/PI | AP3 |
| 88 | ZaMADS88 | 188 | complete | MIKC | AGL61 |
| 89 | ZaMADS89 | 228 | complete | SVP-like | AGL22 |
| 90 | ZaMADS90 | 236 | complete | MIKC | AGL80 |
| 91 | ZaMADS91 | 254 | partial | TM3-like | AGL20 |
| 92 | ZaMADS92 | 210 | complete | AP3/PI | PI |
| 93 | ZaMADS93 | 120 | partial | AP1-like | AGL8 |
| 94 | ZaMADS94 | 222 | complete | AGL6-like | AGL6 |
| 95 | ZaMADS95 | 267 | complete | TM3-like | AGL20 |
| 96 | ZaMADS96 | 180 | complete | TM3-like | AGL20 |
| 97 | ZaMADS97 | 70 | complete | AGL17-like | AGL16 |
| 98 | ZaMADS98 | 187 | complete | AGL15/AGL18 | AGL15 |
| 99 | ZaMADS99 | 168 | partial | MIKC | AGL29 |
| 100 | ZaMADS100 | 203 | partial | AGL32 | AGL32, |
| 101 | ZaMADS101 | 222 | complete | TM3-like | AGL20 |
| 102 | ZaMADS102 | 83 | complete | AGL2-like | AGL2 |
| 103 | ZaMADS103 | 141 | partial | TM3-like | AGL20 |
| 104 | ZaMADS104 | 255 | complete | AGL15/AGL18 | AGL15 |
| 105 | ZaMADS105 | 154 | partial | AGL15/AGL18 | AGL15 |
