# Supplementary material for: Genome-wide identification, interaction of the MADS-box proteins in Zanthoxylum armatum and functional characterization of ZaMADS80 in floral development
Source: Front Plant Sci. 2022 Nov 25;13:1038828. doi: 10.3389/fpls.2022.1038828 (PMC9732391; doi:10.3389/fpls.2022.1038828)
Supplement: Additional File 1 — The amino acid sequences used in phylogenetic tree construction for M-type MADS-Box genes. [file Table_3.docx]

>AGL28

MARKNLGRRKIELVKMTNESNLQVTFSKRRSGLFKKGSELCTLCDAEIAIIVFSPSGKAY

SFGHPNVNKLLDHSLGRVIRHNNTNFAESRTKLRIQMLNESLTEVMAEKEKEQETKQSIV

QNERENKDAEKWWRNSPTELNLAQSTSMKCDLEALKKEVDEKVAQLHHRNLNFYVGSSSN

VAAPAAVSGGNISTNHGFFDQNGNSTSAPTLPFGFNVMNRTPAGYNSYQLQNQEVKQVHP

QYWARYY

>AGL100

MKDLFMEGERETSSMTCLTPKDSVQSPNMLVRQPKKETTTQTPKTTRGRQKIEIKKIEEE

TKRQVTFSKRRRGLFKKSAELSVLTGAKIAVITFSKCDRIYRFGHVDALIDKYLRKSPVK

LEGYSGDNAADEESRRPWWERPVESVPEEELEEYMAALSMLRENIGKKIVAMGNDRTVDM

VPAWPINVMGWKPTMDMQKLENLTDGVNRCRVGQNGD

>AGL87

MGRRKVTHQLISDNATRRVTFRKRKDGLLKKIYELTVLCGLPACAIIYSEYKDGPELWPN

LNEVRSILNRLSELPVEKQTKYMMDQKDLMNKMIQDAEKKLEKEKMHTRAMKLGLMAGSN

DLITDTDCSEELARAADVVDKKLKAIRERIKAVEAGAPIIKRD

>AGL58

MNPKKTKGKQKINIKKIEKDEDRSVTLSKRLNAIYTMIIELSILCGVEVAFIGYSCSGKP

YTFGSPSFQAVVERFLNGEASSSSSSSLQRSVKNAHKQAKIQELCKRYNRLVEELKVDEV

KVKKAAALAETRAVNKDAWWKADPNDVKDHEKAKKMMEKYQELKEKLREEVALRIKRGHD

ENNNK

>AGL59

MNPKKTKGKQKINIKKIEKDEGRSVTFSKRLNGIYTKISELSILCGVEVAFIGYSCSGKP

YTFGSPSFQAVAERFLNGDASSSSSSSLVMNAHKQAKIQELCKKYNRLVEELKVDEVKVK

KAAALAETRVVNKDVWWKVDPNDVKDHEKAKKMMEKYQELYDKLCEQAASRIKRGHDENN

NK

>AGL64

MKPKKTKGKQRINIKKIEKDEDRLVTLSKRRNGIYTKLSELSILCGAEVAFLGYSCSGKP

YTFGSPSFQAVAERFLNGEASSSSSSSLQRSVMNAHQQAKIQELCKVYNRLVEEITVEEV

KLKKTAALAEMMPMNEDAWWKVDPNDVKDREEVKKMMEKHQELYEKLCEEAASRIKRGHD

ENNNK

>AGL86

MRSKIKLSLIANKTSRRTTFRKRKGGITNKLHELTTLCGVKACAVISSPYENPVVWPSTE

GVQEAVSMFMERPATEQSKLMMSHETYLQDKITKETKKLESLRRENRESQLRQFMFDCVE

GKMSEHQYGARDLQDLSLYIDHYINQLNSSVMLLTNNGASSSSFPPPLHTSVAGAGAGAG

AAPLVVAGAGAAPLAVAGAGASPLAVAGVGAAPLAVAGAGPPMAQNQYEPIQPYIPTAFS

DNIQYQAPVDFNHQIQHGIYDNLSLDPNHQYPFQDDPFMEMLMEYPYEQVGYAAEHAHIP

FMNGNYYNYHQPPTVGLTTTGHMPSNNATTTTTTNTTVV

>AGL92

MRTKTKLVLIPDRHFRRATFRKRNAGIRKKLHELTTLCDIKACAVIYSPFENPTVWPSTE

GVQEVISEFMEKPATERSKTMMSHETFLRDQITKEQNKLESLRRENRETQLKHFMFDCVG

GKMSEQQYGARDLQDLSLFTDQYLNQLNARKKFLTEYGESSSSVPPLFDVAGANPPVVAD

QAAVTVPPLFAVAGANLPVVADQAAVTVPPLFAVAGANLPVVADQAAVNVPTGFHNMNVN

QNQYEPVQPYVPTGFSDHIQYQNMNFNQNQQEPVHYQALAVAGAGLPMTQNQYEPVHYQS

LAVAGGGLPMSQLQYEPVQPYIPTVFSDNVQYQHMNLYQNQQEPVHYQALGVAGAGLPMN

QNQYEPVQPYVPTGFSDHFQFENMNLNQNQQEPVQYQAPVDFNHQIQQGNYDMNLNQNMK

HAHIPFMDGNYYNYHQPPTVGLTSTGHMPSTTTTTTNNNNNNNV

>AGL97

MGGVKRKIAIEKIQNKNPRAVSFSKRRKGLYSKASELCLLSDAEIAIIATPVSSNSNAAF

YSFGHSSVDNVVAAFLANQRPCDERFWWEDESLLKSENLEELREAMDSMSTMLRDLKELE

KQRDHQTQTLIHQPCSARVCIQDYVTVNFDGFNTEEQTLAVSDNSNNNGLLGNLDECNED

FDDLDQIFDTVTNSEFLSVNLEMDDVTVNSEGNTEEQTLAVSDNSNNNGLLGNLDECNED

FDDLDQIIEYLTSSEALSMNLKMDDV

>AGL102

MGRRKIEIKFIEDSIERKATFSRRRNGIFKKADELAKLCNVEIAVLVISPTNIPYTYGYP

CFNDVVERIQNPSASSKLRSLMKELEQIKEFQEDLRKKQQRNLEKSNMKENVDLKLEDLV

AFKAKLEAYQAGLKRKHVEMEDLSSPSILSKNTKNKMMRTEYSSGQSKGMYEFRAFGPGF

LGTI

>AGL85

MKTDWSHYLSVEMESTISNELSILCGAEVAFLGYSCSGKPYTFGSPSFQAVAERFLNREA

SSSLQRSVMNAHQQAKIQELCKVYNRMVEEAKTEEAKVKKAAALAETMPVDEDAWWKVDP

KEVEDHEEAKKIMEKCEGLYEKLCNEAAARIQRGDAENNNK

>AGL50

MAPRQKKPNKSDDDDDLRRKKQSFFKQRFPGFKKKASELSVLCGNSVGFICYGPDSDLHV

WPQSQDHNPQALHEIVAKFNALSDERRKNHACDLNDFPHHLKGLSREELRKHLLHLDSQL

LGVREQKIEILKKTLTGSSEKDGARVSENSAISDHKLKIEPHLKDILSEDHLIRVSDKKL

GSCDVFDELAYVVRGSRNLNENVSKYESKDADNTGLDHLVTLGGDYLQEAAAELYQTYNL

GNFCDDHVWDLEFASRLPLLHTFSDPLMTTNTCQTMSTDMISI

>AGL49

MAPRQKKPNKSDDDDGDLHRKKQSFFKQRFPGFKKKASELSVLCGNSVGFICYGPDNDLH

VWPQSQDHNPQALHEIVAKFNALSDERRKNHACDLNDFPHHLKGLSREELRKHLLHLDSQ

LLGVREQKIEILKKTLTGSSEKDGARVSENSAISDHKLKIEPNLTDILSEDHLIRVSDKK

LGSCDVFDELAYVVRGSRNLNENVSNYESKDAAYTGMDHLGTFGGNYLQEAAAELYQTYN

LGNFCDDHVWDLEFASRLPPLHTFSDPLMTTNTCQTMSSDMISI

>AGL56

MGGKKTKIEIKKIINKPAKTVAFTKRREGLFRKASQLCLLSPATQIAILAAPMTSKSHAS

FYSFGHSSVDNVVSSLLYDHPPLTANQDNRSGLGFWWEDKRFDVSENVEELKEAVDAVSR

MLNNVRCRLNDAVKSTQRDGGLEILHHQEEEVLQTRNDETKTNQTHEFEGGETSGSASWL

ENEDDILHFDDDFYTGIDPLF

>AGL55

MGGTKRKIEMKRIEDKNVRAVAFTKRKSGLFHKASELCLLSPGTQIAILATPLSSHSHAS

FYSFGHSSVDHVVSSLLHNQHPSLPTNQDNRSGLGFWWEDQAFDRLENVDELKEAVDAVS

RMLNNVRLRLDDAVKSNQRDGSLVIHQEDEEVLQLGYKDTNQITKLEGETSASASLLKNV

VDNLHIDDRYY

>AGL38

MKRKMKLSLIENSVSRKTTFTKRKKGMTKKLTELVTLCGVEACAVVYSPFNSIPEAWPSR

EGVEDVVSKFMELSVLDRTKKMVDQETFISQRIAKEKEQLQKLRDENHNSQIRELMFGCL

KGETNVYNLDGRDLQDLSLYIDKYLNGLTRRIEILIENGESSSSLPLPIVANAAAPVGFD

GPMFQYHNQNQQKPVQFQYQALYDFYDQIPKKIHGFNMNMNKDSNQSMVLDLNQNLNDGE

DEGIPCMDNNNYHPEIDCLATVTTAPTDVCAPNITNDL

>AGL37

MRGKMKLSFIENDSVRKTTFTKRKKGMLKKFNELVTLCGVDACAVIRSPYNSIQEPWPSR

EGVEEVMSKFMEFSVLDRTKKMVDQETFLRQRIAKETERLQKLRDENRNSQIRDLMFGCL

KGEVDVSHLHGRDLLDLNVFLNKYLNGVIRRVEILKENGESSSSVPPPIGVAPTVVDASV

PIGFDGRMIQDQNQNQQEPVQFQYQALYDFYDQIPKKLHDFNMKMNIDPNQSMNLDLNDG

EDEGIPCMDNNNYHPEIDCLATVTTAPTDVCAPNIINDL

>AGL23

MVKKTLGRRKVEIVKMTKESNLQVTFSKRKAGLFKKASEFCTLCDAKIAMIVFSPAGKVF

SFGHPNVDVLLDHFRGCVVGHNNTNLDESYTKLHVQMLNKSYTEVKAEVEKEQKNKQSRA

QNERENENAEEWWSKSPLELNLSQSTCMIRVLKDLKKIVDEKAIQLIHQTNPNFYVGSSS

NAAAPATVSGGNISTNQGFFDQNGMTTNPTQTLLFGFDIMNRTPGV

>AGL61

MYVTKYKNKLPTLQVDLIIMPHQTQACIYKQTLSLHQIFQQRKATKTLHTKKTMMSKKKE

SIGRQKIPMVKIKKESHRQVTFSKRRAGLFKKASELCTLCGAEIGIIVFSPAKKPFSFGH

PSVESVLDRYVSRNNMSLAQSQQLQGSPAASCELNMQLTHILSEVEEEKKKGQAMEEMRK

ESVRRSMINWWEKPVEEMNMVQLQEMKYALEELRKTVVTNMASFNEAKDDVFGFLDNKVT

VPPYVNMPSGPSNIYNFANGNGCF

>AGL46

MARKKLNLTYIFNDRMRKRSFKQRREGFLKKLNDLKVLCDVNACAVVYNPFNSNPDVWPS

KSEVNNIIKKFEMLPETQKKVKSVNHEEFLNLYISKVEKQSKKLIVENKETCLKEVMFKC

LGGNMGDFVMNDNDRLDLCKFIDHYLRNLYHHKNVTLNNPNFEIGESSSLMDMAPTATTG

NMATTVVDEGMTPLLIAEGSSSSFLNSPLFNSPQLTNELQLIVSQNHRLENSLASNLFFS

EGQDICIPDMNQSIIPSNQGAEHVDFLESNFLPNNNQEVYIPVMDQDEVYNPNQNHYENQ

QGFIDEMMKYAEKTSFPWMVENHCYNHNQ

>AGL29

MGRRKIKMEMVQDMNTRQVTFSKRRTGLFKKASELATLCNAELGIVVFSPGGKPFSYGKP

NLDSVAERFMREYDDSDSGDEEKSGNYRPKLKRLSERLDLLNQEVEAEKERGEKSQEKLE

SAGDERFKESIETLTLDELNEYKDRLQTVHGRIEGQVNHLQASSCLMLLSRK

>AGL48

MTRKKVKLVWIENDKSRATSLQKMRVGLLKKVKELTILCAVRAIVIIFSPDKVGPLVWPS

PQATHGLLDEFFALPKSVQKKKESNVESYLKEKTHKFQEQLKKSKKKNKEHVIDELMMQL

QSGREIADLNQSEMYALLSFSRDTILLCRKKLAFMQFPPLRDPPVFPFEIQVEEFKTTTN

DGFVGGGQDNKRAGRTDEATRFINTDIFKQSKSYYFFDEWVFPPSPPKYEIPQQMENGNP

NPKSYRLYQGSSSNGNPHLEMDPFRLQMMTSQGLAGSVSQPLQHHSMINNPTMAMNQPSQ

DPFDYMRSELGINEGININNSQFYMSNNTITANDGVRQEPYPNVTTAGENNGDATTSNTN

MVWPGFNNHHF

>AGL57

MSSTKQAKGRKTKGKQKIEMKKVENYGDRMITFSKRKTGIFKKMNELVAMCDVEVAFLIF

SQPKKPYTFAHPSMKKVADRLKNPSRQEPLERDDTRPLVEAYKKRRLHDLVKKMEALEEE

LAMDLEKLKLLKESRNEKKLDKMWWNFPSEGLSAKELQQRYQAMLELRDNLCDNMAHLRL

GKDCGGSSSVRVGRRVSGGVRLFDREA

>AGL45

MTRKKLNLSYITNESMRKATFNKRKKGLVKKIHELSVLCGIEACAVIYSPFNSNPEVWPS

NSEVKNVMENFEMLTKLEQEKKMVSHEGFIRQNISKTMESNNKKMIDNAERTMKEAMFQL

LSGKGEKLNLTDRNREDLCKYIDQYLKELYHHKNKTINQSHIEPGESSGATNAMTPTSVV

EPIISSIQRPNQNPNFNHLSHNQYQYQQQFGYPILVQDGIYNPSQIQNQHEEWLDDHMMN

HSKEISHPLMDDNNFYYQQP

>AGL103

MASSSSSSLSFSTSKKNKTFFKKPNSAFSSSRATSLIKRQQTVFKKAKELSILCDIDVCV

ICYGSNGELKTWPEEREKVKAIARRYGELSETKRRKGSVDLHEFLEKMNKDDPEKEEKKK

IKVRRVPKVKYPVWDPRFDNYSVEQLMGLVQSLERNLTRIQHRTCAVVEAQGQRRVQYTN

MANQELMMANTMNQLQQHSNQVSMYLWNHGNGAFSQIPVSALASNQTQSLAPIPPELMIY

PNSDAGNYSGSLGVQGTGINGLQNMNMLTYNNINSVNDFSKQFDQNSRAESYSSLLGVHE

DGNNEFENPNMSSRNNFNVQDCAGLLGMQGAGTNGLQSMNMHDYSNNNSINSNGLSHQYV

QFPTYNSQHQDRVFNLDQNGNNTRSL

>AGL91

MGRRKIKMEKVQDTNTKQVTFSKRRLGLFKKASELATLCNAEVGIVVFSPGNKPYSFGKP

NFDVIAERFKNEFEEEEEGDSCETSGYSRGNRARQEKKICKRLNSITEEAEAEKKHGEDL

HKWLESAEQDKFNKPIEELTLEELKEFEAKIKKISCGIQSNISHMQASSSLMFLSNDN

>AGL51

MKQSSFSSSSSSRNSTSLTNRLKTIFKKAEELSILCAIDVCVIYYGPDGELRTWPKERNT

VKDMASRYKEATKRKKKRTLSTLQERLRIVESQKQQNKNLVHQSLTPSYLNQIQHLNPSN

FSPYMYNHGDAATLSQLPLSASLSNQLQLPESLDAAWFWSEHVFGQHHQQQQLSTSWRVK

HTRILTVSFGTSICSE

>AGL52

MKQASSSSSCNPTSLTNRLKTIFKKAEELSILCAIDVCVIYYGPDGDLRTWPKDRETVKN

MALRYKEDRKRKKCLNLHEFLEKEKVKDKDKYKGKTNYVKNPNWYPNFDHYSPQQLSQLI

QSLERTLSTLQKRLRIVESQKKQNTNLVHQSLTPSYLNQTQHLDPSKFSLYMYNHGDATL

SQLPLSASQSNQLINYQMQHGFGQNMCLDNITNNNNFQHPGVSNTQDYSPLLSANNYGLN

NHLMQQQDQLHGFDQNLCMMSEIINNNNGLQHPNLSNTVPHEFPYGNTSFSQDMFSSYDG

SSLLQTSSLPPLHNIPNSYCFSDNSRLLC

>AGL40

MVRSTKGRQKIEMKKMENESNLQVTFSKRRFGLFKKASELCTLSGAEILLIVFSPGGKVF

SFGHPSVQELIHRFSNPNHNSAIVHHQNNNLQLVETRPDRNIQYLNNILTEVLANQEKEK

QKRMVLDLLKESREQVGNWYEKDVKDLDMNETNQLISALQDVKKKLVREMSQYSQVNVSQ

NYFGQSSGVIGGGNVGIDLFDQRRNAFNYNPNMVFPNHTPPMFGYNNDGVLVPISNMNYM

SSYNFNQS

>AGL99

MGGVKRKISIELIEKKDSRAVAFSKRSRGLYSKASDLCLLSDAQIAIIATPVSSKSNVSF

YTFGHSSVDNVVAAFLTNQRPREGLGLDYWWEDERLSKSEDLEELRDAMDSMSKMLKDLK

DLQNQRDCEEDVKKKGVLHGTHQKQTFNPESCSVNFDGFNKNTEEFDLDEIFDYVSTAEA

LSMNLDMDDVSVVTTNQNPVSASETVEDRELVVHKNMDEDNIHVSDMDDKDTMLMISDKN

NVLPENLDEFDQELDLDQLLDFETNYESLLKSCEMEDYASMVTTKQNLCSNPEAVEDGGL

MIQKDLPEDNLCFSDYFSDLHC

>AGL96

MARKKVRAAWIRDDRMRRASLKRRLTGLIKKVNELSILCDMRASVVVFNREEEQLTAWPS

PEAANSLIDNFYSLTDHERTMKAVDPESYVQTVIEKIEKKRADTRKVITEFEMDELMFQV

QNGRELADLSPTEADKLIPYADKKLMWLSKRMGSTGVDALRASNVASGSGGNGLNMMETG

RSFYYVDKWVFVDPQVQNPCDVETHLPTMVSGLDLNMEPSDEDLGTYKGESSMAGGAEDD

AE

>AGL34

MGMKKVKLSLIANEISRETSFMKRKNGIMKKLYELSTLCGVQACTLIYSPFIPVPEFLEM

SPTARTRKMMNQETYLMERITKAKEQLQNLVGANQELQVRRFMFDCVEGKMSQYRYDAKD

LQDLLSCINLYLDQLNGRIEILKEHGDSLPSVSPFPTRIGVEETGDESSSDSPILATTGV

VDTPNATNPRVLVADTTHFLDANATAVTAPFGFSNHIQYKNMNMSQDLHRPFQHLVPTNF

CDFFQNQNMNQVQYQAPPNDMFNQIQREFYNINLNQKSNQYMNQQQPFMNPMVEQHMSHV

GGRESIPFMDGNYYNYNQLPVVDHGSTSYMPSTTGVYDPYFNNNL

>AGL35

MTRQKVKMTFIENETARKSTFKKRKKGLLKKAQELGILCGVPIFAVVNSPYELNPEVWPS

REAANQVVSQWKTMSVMDKTKKMVNQETFLQQRITKATESWKKLRKENKELEMKNIMFDC

LSGKTLVSSIEKTELRDFGYVIEQQLKDVNRRIEILKRNNEPSSALVPVAAPTTSSVMPV

VEMGSSSVGFYDKVRDQIQITLNMKQTTNDLDLNKKQW

>AGL36

MKKVKLSLIANERSRKTSFIKRKDGIFKKLHELSTLCGVQACALIYSPFIPVPESWPSRE

GAKKVASRFLEMPPTARTKKMMDQETYLMERITKAKEQLKNLAAENRELQVRRFMFDCVE

GKMSQYHYDAKDLQDLQSCINLYLDQLNGRIESIKENGESLLSSVSPFPTRIGVDEIGDE

SFSDSPIHATTGVVDTLNATNPHVLTGDMTPFLDADATAVTASSRFFDHIPYENMNMSQN

LHEPFQHLVPTNVCDFFQNQNMNQVQYQAPNNLFNQIQREFYNINLNLNLNLNSNQYLNQ

QQSFMNPMVEQHMNHVGGRESIPFVDGNCYNYHQLPSNQLPAVDHASTSYMPSTTGVYDP

YINNNL

>AGL26

MESCCRSVIASRTFHLRSSGRLFPSLSLTHLKGKLSLSINSFSSKIQSHALRGVGIGESD

KKNPLPRGAGEGVKEDARSKLLHVVLVSPQIPGNTGCIARTCAASAVGLHLVGPLGFQVD

DARVKRAGLDYWPFVVVKAHSSWAEFQEYFRLQEGEKRMIAFTKRGTRIHSDFSYRSGDY

LLFGSETSGLPPEALSDCNHEPYGGGTLRIPMVETYVRCLNLSILRCQEENQIFCEKKPN

LSMREDTMFKKALELSTLCDIEVCVILYSRDGELIKTWPEDQSKVRDMAERFSKLHERER

RKKRTNLSLFLRKKILDNSKLSEKVLEMKDSLESGLRVLQDKLLLLQPEKNQTELGQIPV

INNGQNHW

>AGL93

MDSSMSTKKKTKLSVRNQTCFKKSSLSSSSTAKKTTNLSMREQTMFKKALELSTLCNIDV

CVIYYGRDGKLIKTWPDDQSKVRDMAERFSRLHERERCKKRTNLSLFLRKKILDDTKLSE

KVLEMEDSLESGLRVLQDKLLLLQPEKNQTEFGQTRAVSSTTNPLSPPPSLIEDHRHQQR

TEPLMSGVSNTEQDLSTSSLSQNQSKFSVFLYNHDNCSFYQVPDSVSSFDSLTSTGLLGE

QGSGLGSSFDLPMVFPPQMQTQTPLVPFDQFAPWNQAPSFADPMMFPYN

>AGL101

MFKKALELSTLCNIEVCVIYYGRDGELFKTWPEDESKVRDMAERFTKLNERERRKKRTNL

SLFLRKKILDDNKLSGKVLEMKDSLERGLRVLQDKLLLLQPENQTKSLTRSVSSLDYVFV

>AGL53

MDSSMSTKKKTKLSVRNQTCFKKSSLSSSSTAKKTTNLSMREQTMFKKALELSTLCNIDV

CVIYYGRDGKLIKTWPEDQSKVRDMAERFSRLHERERCKKRTNLSLFLRKKILDDTKLSE

KVLEMEDSLESGLRVLQDKLLLLQPEKNQTEFGQTRAVSSTTNPLSPPPSLIEDHRHQQW

TEPLMSGVSNTEQDLSTSSLSQNQSRISVFLYNHDNRSFYQVPDSVSSFDQSALLGEQGS

GLGSNFDLPPMVFPPQMQTQTPLVPFDQFAAWNQAPSFADPMMFPYN

>AGL54

MDSSTSTKKNTKLFVRNQTCFKKSSLSSSNAKKTTNLSMREQTMFKKALELSTLCDIEVC

VIYYGRDGKLIKTWPEDQSKVRDMAERFSRLHERERCKKRTNLSLFLRKQILHDKKLSEK

VLEMEDSLESGLRVLQDKLLLLQPEKNQTELGQSCAVYSTTYPLSSPSLIEDHQHQQQWT

EPLSNTE

>AGL39

MPSSDSTMMKKGTKRKIEIKKRETKEQRAVTCSKRRQTVFSKAADLCLISGANIAVFVTS

PSDSSDVVYSFSGYSSAYEIADCYLNRKPPPKIVNPAGSKLGFWWEDPDLYHSCDDLSEL

SIIEDRLQRMKKHVMACLEKEEKSQLVSSFDQNPNSTCSLDVEDCDGSSYSQIASTFTPN

SVNEYCSDQTFSSFHGDQNPNLSSPSFDQDCYSSLYQICGESSSQVASFDQNPSSEIQGF

ETEEEINQINLLLQETQTEANVNLDDEICFWNDLSNDDVFGLNSYFGLDNTNAMINFGDS

DFRRHV

>AGL89

MDSSMSTKKKTKLSVRNQTCFKKSSLSSSSTAKKTTNLSMREETMFKKALELSTLCDIEV

CVIYYGRDGELIKTWPEDQSKVRDMAERFSKLHERERRKKRTNLSLFLRKKILDDNKLSE

KVLEMKDSLESGLRVLQDKLLLLQPENQTELGQSRAVSSTTNPLSSPEDHHHQQWTEPLV

TGVSNTEQDLSTSPLSNHQSKYSVFVYNHDSGSFYQVPDSICF

>AGL90

MKKVKLSLIANERSRKTSFMKRKNGIFKKLHELSTLCGVQACALIYSPFIPVPESWPSRE

GAKKVASKFLEMPRTARTRKMMDQETHLMERITKAKEQLKNLAAENRELQVRRFMFDCVE

GKMSQYRYDAKDLQDLLSCMNLYLDQLNGRIESIKENGESLLSSVSPFPTRIGVDEIGDE

SFSDSPIHSTTRVVDTPNATNPHVLAGDMTPFLDADANANMNQVQYQAPNNLFNQIQREF

YNINLNLNLNLNSNQYLNQQQSFMNPMVEQHMNHVGGRESIPFVDRNYYNYNQLPAVDLA

STSYMPSTTDVYDPYINNNL

>AGL105

MRDPDIVQNTRSLILLRVLTHEKLIQSHRYFFVGWIHKNLSRFRRKIRRRFLHCDFSGFG

FFPRRFLFGFVLEKMDHLQESFRGSELCILCDIEACVIYYGPDGELKTWPKEREKVEDIA

LRYSQLNEALRRKKSVTLYDFLNKKKDKTNLEKKAMITDNDDLKTCLKNVNVLKSPIADH

YFNDQISQLIQSLEPHVSKVQERIRFVESQKHKETKLDHQSLASIYSLNQSLNPSQFTLF

LYNHGDNTMSQIPNMFMNNNNFQHSFVSNTQDYSALQESVNNNYGLMPNVLCGYDQNLFT

SDITNNNLLIDNSMYL

>AGL73

MVKGTKRKIAIETIQKRDSLRVTCTKRRKGLYSKASQLCLLSDAQIAILATPPSSESDVS

FYSFGHSSVDAVVSAFLSGKRPVSAPKDNKETREDVGICLTRKNLGLGFWWNDESLVRSE

NPQEISEAIGSMWTLLSNLKELRADEACVNDHKDLKKNEKSDVHGTQDQTLIFQSASAVC

CIPENLNDITQEPNQTLDIQSSSSAICCVPDKSPEIFNEITEEQDQILSICETFCVTDNN

NNNNNNAALPEVNLYYNQDMAIDQLIDFNTPFESSIDDWFSDNTTHQETTSASILNDVGV

DDQVSVDTNPFSYFQSLEDADLVFQRCLDGDNLRFSDCFNDFANTIAAV

>AGL77

MTTIRSSPSSSRCSNSSSSSSYSLASTSLSNRLETIFKKASELCTLCDIEACVIYYGPDG

ELKTWPKEREKVRDIALRFNQLNEALRHKKSVNLHGFLNKKKKNKGLKNPNKKKKTSLKN

VNVLKYPLADHYSPDQVSQLTQSLELNVSKFQERLRFLESQKQNETKPDHQSLTSISSLN

QSLNPSQFSLFMYNHGYNTLSQIPVSASNFNQDYISALLEQSELKSQIMKQEVCGYEQNM

CMSNHGDATLSQIPFSASNFNQDFSANNNFQHSFVSNTQDYYSVQKSVNNNYGLKNQLMK

HDLCGYEHNMCMSNHGDATFSQIPLSASNFNQDFSVSIQEESGLMQQELCGYDQNQNMSM

GDITNNNFQVTCASVLESVNNFGLNQLMHKEFYGCHQNMSMGNINNNSFQHPWVSNADHT

RRYKNL

>AGL81

MAIRSLPSSSRCSSSSSSSSYSLASTSLSNRLETIFKKASELCTLCDIEACVIYYGPDGE

LKTWPPEREKVEDIALRYSQLNEALRRKKSVTLYDFLNKKKDKTNLEKKAKITDNDDLKT

CLKNVNILKYPLADHYSPDQVSQLIQSLEPHVSKVRERIRFVESQKHKETKPDHQSLASS

SLNHQTQSLNPSQFSLFMYNHGDNTLSQIPVSASNFNQDYFSALLEQSELKSQIMKQDLC

GYEQNMCMSNHGDATLSQIPLSASNLNQDFSALLQDESGLMQQELCGYDQNMFMNNNNFQ

HSFVSNTQDHSAPVVQESVNNNYGLMPHVPCGYDQNLFTSDITNNNLLINNSMFL

>AGL98

MAIRSLPSSSGCSNSSSSSSYSLASTSLSNRLETIFRKASELCTLCDIEACVIYYGPDGE

LKTWPPEREKVEDIALRYSQLNEALRRKKSVTLYDFLNKKKNKTNLEKKAKIKDNDLKRL

SLEPHVSKVRERIRFVESQKHKETKPDHQSLASSSLNNQTQSLNPSQFSLFMYNHGDNIL

SQIPVSASNFNQDYFSALLEQSELKSQIMKQEVCGYEQNMCMSNNGDATLSQIPLSASNF

NQEFSALLQEESGLMQQELCNYDQNMFMNNNNFQHSFVSNTQDHSAPAVQESVNNNYGLM

PHVPCGYDQNLFTSDITNNNLLIDNSMFL

>AGL76

MTMRSLPFSSSSYSLASTSLSNRLETIFKKASELCTLCDIEACVIYYGPDGELKTWPKEK

EKVRDIALRYSQLNEALRSKKSVNLHGFLNKKKKKKKKGLKNPNNKRKTCLKKNVNVLKY

PLADHYPPDQVSQLTQSLKLHVSKFQERLRFLESQKQTKPDHQSLTPSSLNHQTQSLNPR

QFSLFMYNHGDNTLSQISVSASNFNQNYFSALLEQSELKNQLMKQDGYDQNQNMRMGDIT

NNNFQLPYFSKKEAVQESVNYFGMNQLMLKELYGCDQNMCMGNINSNSFQHPCVSKAQHY

SAVEGSVNNQRQSELMQQELCGYEQNMCFTNNNFQVSNKEAVQESVTNFGLMQHELYGCD

QNMSMGNIINNSFQQRLKHRTRICE

>AGL43

MTMRSSLPSSSSAYSLASTSLSNRLETIFKKASELCTLCDIEACVIYYGPDGELKTWPPE

REKVRDIALRYSQLNEALRRKKSVNLHGFLNKKKKNKGLKNTDKKRKTSLKKVNVLKYPL

ADHYPPDQVSPLIQSLELHVSKFHERLEFLESRKQNETQPDHHSLASSSLNHQTQSLNPS

QFSLFMYNHGDNTLSQIPVSASNFNQDYFSALLEQSELKNQLMKQEICGNDQNQNMWMGN

ITNNNFQLPCVSVQESVNNFGLMHKEFYGCDHNMSVGNINSNSCEHPCVSSTQHYSAVEE

SVNNPWLNQLMQNELYGYGYADFC

>AGL75

MTMRSSSPSSSSSYSLAFTSLSNRLETIFKKASELCTLCDIEACVIYYGPDGELKTWPKE

KEKVRDIALRYSLLNEALRRKKSVNLHGFLNKKKNKGLKNPNKKMKTSLKNVNILKYPLA

DHYPPDQVSPLIQSLELHVSKFQERLRFLESQKQNQTKPDHQSLTPSSLNHYTQSLNPSQ

FSLFMYNHGDNTLSQIPVSASNFNQDYFSALLEESELKNQLMKPEICGYDQNQNMSMGDI

TNNKFQDPCVSNKEAVQESVNNFGLNQLMYKEFYGCDQNMSMGNINSNSFQNPCVSNTQH

YSAVEESVKNPWLNQLMQNELYGYGYAGFC

>AGL80

MTRKKVKLAYISNDSSRKATFKKRKKGLMKKVHELSTLCGITACAIIYSPYDTNPEVWPS

NSGVQRVVSEFRTLPEMDQHKKMVDQEGFLKQRIAKATETLRRQRKDSRELEMTEVMFQC

LIGNMEMFHLNIVDLNDLGYMIEQYLKDVNRRIEILRNSGTEIGESSSVAVAASEGNIPM

PNLVATTAPTTTIYEVGSSSSFAAVANFVNPIDLQQFRHPAAQHVGLNEQPQNLNLNLNQ

NYNQNQEWFMEMMNHPEQMRYQTEQMGYQFMDDNHHNHIHHQPQEHQHQIHDESSNALDA

ANSSSIIPVTSSSITNKTWFH

>AGL84

MVKKGGTKRKIAIETIQKRDSLRVTCTKRREGLYSKASQLCLLSDAQIAILATPPSSESN

VSFYSFGHSSVDAVVSAFLSGQRPVPKDNKETREDVGICLTRNNLGLGFWWNDESLARSE

NPQEISEAIDSMRTLLRNLKELRADEALACNQAFVNDREDLKNNDKCDFVSDHETHDQTL

ILQSASPICCIPENLNEITQEPNQTLNIQSSTSAICCVPDNSPENFNEITEEQDQIRSIC

ETFCVMDNNAALPEMNLDYDQDIGFDTPFESALNDWFSDNTTHQEISASILNAVVDDQVS

VDLTPFSYFQRCLDGDNLRFSDCFKDFANTISAL

>AGL83

MRFVPYLYEIERLWLSLVNYLSPRKNKNRRCGEIDKIRMVKKGGTKRKIAIETIQKSDYL

RVTCTKRREGLFSKASQLCLLSDAQIAILATPPTSESNISFYSFGHSSVDAVVSSFLSGQ

RCVPLQEDTKEMREDVAICLSRTNLGLGFWWNNESLNKSENPQEISDAINSMLTLLSNLK

ELSGEEALVNDHKDLKKNERSDVVLQHGTQYETLNPNSNTTTICCVPDELPANSNEIVGI

SPNPLIMLEKKKSQIEEKFEKEWQVSVTRIENEATSSYAKRRRSI

>AGL47

MGRKMVKMTRITNEKTRITTYKKRKACLYKKASEFSTLCGVDTCVIVYGPSRAGDEMVME

PELWPKDGSKVREILTKYRDTASSSCTKTYTVQECLEKNNTKVEKPTIATKYPTWDKKLD

QCSLNDLYAVFMAVENKIQEATNRNQTFPDTSCWSNDQLGLCGYNRQCFEQYQLFPLPTM

DYNGLSFFPFNNQMTSNTAEVSSFSNVTEPMIANGQSLFYGSCSDGPYGPMVQRTAYMEP

IHWGLGNSMFNNVKQFQDYPFRFAQVNDLEDSSKLSM

>AGL82

MVPKVVDLQRIANDKTRITTYKKRKASLYKKAQEFSTLCGVETCLIVYGPTKATDVVISE

PEIWPKDETKVRAIIRKYKDTVSTSCRKETNVETFVNDVGKGNEVVTKKRVKRENKYSSW

EEKLDKCSREQLHGIFCAVDSKLNEAVTRQERSMFRVNHQAMDTPFPQNLMDQQFMPQYF

HEQPQFQGFPNNFNNMGFSLISPHDGQIQMDPNLMEKWTDLALTQSLMMSKGNDGTQFMQ

RQEQPYYNREQVVSRSAGFNVNPFMGYQVPFNIPNWRLSGNQVENWELSGKKTI

>AGL62

MVKKSKGRQKIEMVKMKNESNLQVTFSKRRSGLFKKASELCTLCGAEVAIVVFSPGRKVF

SFGHPNVDSVIDRFINNNPLPPHQHNNMQLRETRRNSIVQDLNNHLTQVLSQLETEKKKY

DELKKIREKTKALGNWWEDPVEELALSQLEGFKGNLENLKKVVTVEASRFFQANVPNFYV

GSSSNNAAFGIDDGSHINPDMDLFSQRRMMDINAFNYNQNQIHPNHALPPFGNNAYGINE

GFVPEYNVNFRPEYNPNQNQIQNQNQVQIQIQNQSFKRENISEYEHHHGYPPQSRSDYY

>AGL78

MKQASSSSSSRNSTSLTNRLKTIFKKAEELSILCAIEVCVIYYGPDGELRTWPKERETVK

DMALRYKEARKRKKSRNLHEFLEKEKDKDKGKTNLKKNWYPNFDHYSPQQLSQLIQSLER

TLSTLQERLRIVEAQKLQNTNLVHQSLTPSYLNQTQHLNPSKFSLFMYNHGDATLSQLPL

SAPHSNQLINYQNHLMQHGFGQNMCSDNITNNNFEHPGVSNTQDYSPLLSVQASAVNNYG

LNNHLMQQQDQLHGFDQNMCMVSEIINNNNGLQHPNLSNTVPHEFSSDFNQNPYGNAVGN

ISFSQDMFSSYDASSLLQTSSLPPLHNIPSSYCFPGNSRLL

>ZaMADS2

MTRKKVKLAYISNDSARKATFKKRKKGLLKKPEIWPSPLGVQRVLSQFKKMPEMEQSKKMVNQDSFLRQRIAKANEQLKKQRKDNREKEMTQVMFQSLTGKALLSLNMMDLNDLGWLIEQNLKEICKRIETLKNNIATPSSNVVVRNNGETLQHVEKSSGVDQMNMEAMQKQQWFLDLMNPQEHMGFGGEEIMYPFGESSSHNTLWPNPFYP*

>ZaMADS12

MDCQTMKMEPIKHEKARLATFKKRKQSLMKKAYEISTLCDVEVCIILYGPKLEGQSVKLETWSSKEGDLISIIRKYMKKISTGGDHGHQRTLSLFKFKENKARRQVDDAATMVCENNWVRNFTTWHQRIDSLSEDQLKMILGAMDNKIKVADRKLDMIRRDQNLMNKATSRKLDHESHLNDAKSLVNMQSDYQVSQSSKEE*

>ZaMADS19

MMDCQALKLEPIKHDRARLATFKKRKHSLLKKADELATLCDVEACIIIYGPKLKGQSAKPETWTSKDGDLNSIIRKYKKKISAGGDHDQRRMSCLSKFDETRGKQVDDASAARTRRKNCVGNFTTWDQRIESLSEDQLKMILGTMDDKLKVADRKLNMIKLEHDSLSNELQPESSDMETQRRGESGSSITLTQSP*

>ZaMADS4

MKRIENEDDRLITFSKRRSGIYKKASELVTLTGAEIGIVVFSPSGKPFSFGHPSIEAVANRFLGGNHPPTDNTHPLVEAHRQVRIGELNQVHNELLHQLDAERERGKVLKQMSEGKETQHWWETSVEELNMQELLQMNAAFDNLNRMAVTTLNERTAGSGAGAGAGASSSVAPSMNLVPFATNPHEFDSSGINPFSYASKPRRF*

>ZaMADS15

MDCQTMKMEPIKHEKARLATFKKRKQSLMKKAYEISTLCDVEVCIILYGPKLEGQSVKLETWSSKEGDLISIIRKYMKKISTGGDHGHQRTLSLFKFKENKARRQVDDAATMVCENNWVRNFTTWHQRIDSLSEDQLKMILGAMDNKIKVADRKLDMIRRDQNLMNKATSRKLDHESHLNDAKSLVNMQSDYQELCNWEWLLSVVAFLVFFIPIPGISVVWVCVFRFGAHS*

>ZaMADS7

MDKTEATSSIKKKPCGSGRRKVEIKKIESSSSRMVAFSKRKKGIFNKACELYRLCDADIAVVIFSSTGRPFTFGKPSADHVIDRFLRDEYEDYNAEEESLEMLQLGEDDEEEEEEEEEEGFWWEESIEGLSLEELDKFKASLEALRYNLRQLSSFSLTDLEVLHRKSPMAPAKVDSSKKGDAKAQAAKVAKAVKSGPTFKKTAKKIRTSVTFHRPKTLKKDRNPKYPRISAPPRNKLDHYQILKYPLTTESAMKKIEDNNTLVFIVDIRADKKKIKDAVKKMYDIQTKKVNTLIRPDGTKKAYVRLTPDYDALDVANKIGII*

>ZaMADS8

MTRKKVKLAYISNDSARKATFKKRKKGLLKKVSELKTLCGIDACAIIFSPYDPHEPEIWPSPLGVQRVLSQFKKMPEMEQSKKMVNQDGFLRQRISKANEQLKKQLKDNHEKEMTQVMFQGMKGSSQAFLNLNIMGLNDLSSQIEQNLKEIYKMIEILNAKP*

>ZaMADS17

MAPTGKGKCKIEITKIENQESSMVSFSKRPQGLFKKAQEYSNITGAQIAILVFSPVGNPYLHVSPSFDTVSENFLLGGGGGTVAKRVPMTLPRVLDGFGG*

>ZaMADS16

MADLPDPNTGLDQPSPGDDLQGNGVDSRGTKRQRTSMAGDDDDDDDEKGGRERRKIEIKFIQDKSRRHITFSKRKAGIMKKAYELSVLTGTQVLLLVVSETGLVYTFTTPKLQPLVTKAEGKNLIQACLNAPEPAGNENGVDDGVEAQSPDDVPQMPQPQHNMQRAAPAGGYMTPEQQQQYYAQLQQQQQYGGMPTMPQHQRA*

>ZaMADS13

MADITDQHDQTSPTELDDSQNVGNGNATESRGIKRQRPSAGDDDDDDDEKGSRERRKIEIKFISDKSRRHITFSKRKAGIMKKAYELSVLTGTQVLLLVVSETGLVYTFTTPKLQPLVTKSEGKNLIQACLNAPEPTPGNENGVDGGDQVESPEEPPNQHLPPQGNRPGMPQNPHMPNNYMPNMPMDPQQALAYQSYVQRNQAYGSGIPPQPGMPANSHHQS*

>ZaMADS9

MAPTGKGKCKIEIKKIENQKARMVTFSKRRQGLFKKAQEYSNITGAQIAVLVFSPVGNPYVHGSPSFDAVSENFLNGGGASGSREGADDVGRGFLMGLEDKMKRCRSVDDLAAVKAELEGVRVKVLQRLKHVEEVDFVNSLLN*

>ZaMADS5

MGRNKVQHELISKESARNVTFKKRKAGLKKKLNELTTLCGVIACAIICSSFDHQPEIWPSTTEAAQVLDKFNNLPAKRKGKYMMDQNIFLSKNISNLSLRVQKEREKNRGLEMDLMVIECLDGKDLNNFKCLEDLGAVDGLLEEKIKLIDNKIKCEMGLESNPLAANNGNKCTTKEA*

>ZaMADS14

MASEITEHDQNASPQLHDDGNGALNAGDANRGTKRPRPAADDDDDDDDKPGRERRKIEIKFIQDKSRRHITFSKRKAGIMKKAYELSVLTGTQVLLLVVSETGLVYTFTTPKLQPLVTKPEGKNLIQACLNAPEPSSGDANGVEDNQVDSPEDNHAQMPPGQPRGVPNNNPIPQAYMTPEAALQYQNYLQGHQQSQQYPMAQHGMPRHPSQHYPQDQKLG*

>ZaMADS18

MFKKASELCRLCGAEIAVIVFSPKGRPYSFGDRVIDKFMAENDETPAVIDRSEEQMNEDNSSEEMHEELGFWWEQSIDDMDLEEIEKYKSCLEELRYNVAEKIEEMVMRRTCERDFLGVCIASISTPDSFIAASSFLNPSSVKPSAYAFTPALMNS*

>ZaMADS1

MASETVKRQRNRKIKFRDSRNSEANRKLTLKKKAKELATLCNITVCMVCFGPDGEVETWPESKAEVEQVINNYRKNGGKNVRENLSLLGFLETKRADLKEELVMIERGNNKRRKLGET

>ZaMADS3

MDPARTTRSLPHSMITVLPKKKPNGTGRKKIEIKKIENNSSRKVAFSKRRKGMFKKADELCRLCDVEIAIIVFSPKGRPYSFGDHVIDKFLAQNEGELVVITKENVSVEEMHEEEEDFWWEQSIEEMNLEEIKQYKSCLEEVRYNVSLKLE

>ZaMADS11

GSGRRRIEIKFIENKSRRQVTFSRRKRGLMKKAYELTTLTGTQALVLIASETGHVYTFATPKLQPVVTLREGKELIQSCLNAPDNNYPPENNYQNNNPQSSVNMTPGTPNLPKTEPHPHVSALAHHQHNDPHSLQMPMGYQQ

>ZaMADS10

DDKPGRERRKIEIKFIQDKSRRHITFSKRKAGIMKKAYELSVLTGTQVLLLVVSETGLVYTFTTPKLQPLVTKPEGKNLIQACLNAPEPASGNENGIDDSAQVDSPDDNSHVQAQRGAMPQQGGLPYGGGNMAQQPMTPEQAMQYQNYLQGQNQYPPMPHNQMGGHRQQ

>ZaMADS6

MAGQKRKIPMKRRETKEQRAVSFSKRRHGLLNKAAEFCLRTRSQVALLVTSPVSKETLYAYGHSSVDAVFDSFMNNRLPDAAGNESIVRSGILLFNEIKDMEHEKAKDKSKRKRDCYESE
