# Supplementary material for: Genome-wide identification, interaction of the MADS-box proteins in Zanthoxylum armatum and functional characterization of ZaMADS80 in floral development
Source: Front Plant Sci. 2022 Nov 25;13:1038828. doi: 10.3389/fpls.2022.1038828 (PMC9732391; doi:10.3389/fpls.2022.1038828)
Supplement: Additional File 2 — The amino acid sequences used in phylogenetic tree construction for MIKC MADS-Box genes. [file Table_4.docx]

>AGL94

MGRVKLKIKKLQNMNGRQCTYTKRRHGIMKKAKELSILCDIDVVLLMFSPMGKASICIGK

HSIGEVIAKFAQLSPQERAKRKLENLEALRKTFMKANHDIDISKFLDRISTPTVEVLSEK

IRFLQTQLSDIHTRLSYWTDVDNIDSVDVLQQLEHSLRQSLAQIYGRKASMPQRQQQQLM

SSQCKNQLQTEIDIDFGMEMEQQLENFSWVRTDENMNVPIEEEDPNLQLHHMYKDITCSA

SSALGNYSGLFSKSSDILQKLETGSIPGTSADPNQQFSNLSFLNDQKLKQLAEWNLLGSP

ADYYVSQILEASYKPQIGGKNNGASSETLPYVAVFDDPLYFWPN

>AGL30

MGRVKLKIKKLENTNGRQSTFAKRKNGILKKANELSILCDIDIVLLMFSPTGKAAICCGT

RRCFSFESSELEENFPKVGSRCKYTRIYSLKDLSTQARILQARISEIHGRLSYWTEPDKI

NNVEHLGQLEISIRQSLDQLRAHKMQDGIQIPLEQQLQSMSWILNSNTTNIVTEEHNSIP

QREVECSASSSFGSYPGYFGTGKSPEMTIPGQETSFLDELNTGQLKQDTSSQQQFTNNNN

ITAYNPNLHNDMNHHQTLPPPPLPLTLPHAQVYIPMNQREYHMNGFFEAPPPDSSAYNDN

TNQTRFGSSSSSLPCSISMFDEYLFSQMQQPN

>AGL65

MGRVKLKIKRLESTSNRQVTYTKRKNGILKKAKELSILCDIDIVLLMFSPTGRATAFHGE

HSCIEEVISKFAQLTPQERTKRKLESLEALKKTFKKLDHDVNIHDFLGARNQTIEGLSNQ

VAIYQAQLMECHRRLSCWTNIDRIENTEHLDLLEESLRKSIERIQIHKEHYRKNQLLPIE

CATTQFHSGIQLPMAMGGNSSMQEAHSMSWLPDNDHQQTILPGDSSFLPHREMDGSIPVY

SSCFFESTKPEDQICSNPGQQFEQLEQQGNGCLGLQQLGEEYSYPTPFGTTLGMEEDQEK

KIKSEMELNNLQQQQQQQQQQQQQDPSMYDPMANNNGGCFQIPHDQSMFVNDHHHHHHHH

HQNWVPDSMFGQTSYNQVCVFTPPLELSR

>AGL104

MGRVKLEIKRIENTTNRQVTFSKRRNGLIKKAYELSILCDIDIALIMFSPSDRLSLFSGK

TRIEDVFSRFINLPKQERESALYFPDQNRRPDIQNKECLLRILQQLKTENDIALQVTNPA

AINSDVEELEHEVCRLQQQLQMAEEELRRYEPDPIRFTTMEEYEVSEKQLLDTLTHVVQR

RDHLMSNHLSSYEASTMQPNIGGPFVNDVVEGWLPENGTNQTHLFDASAHSNQLRELSSA

MYEPLLQGSSSSSNQNNMSECHVTNHNGEMFPEWAQAYSSSALFASMQQQHEGVGPSIEE

MMPAQQSDIPGVTAETQVDHEVSDYETKVPQLSSQ

>AGL9

MGRGRVELKRIENKINRQVTFAKRRNGLLKKAYELSVLCDAEVALIIFSNRGKLYEFCSS

SSMLRTLERYQKCNYGAPEPNVPSREALAELSSQQEYLKLKERYDALQRTQRNLLGEDLG

PLSTKELESLERQLDSSLKQIRALRTQFMLDQLNDLQSKERMLTETNKTLRLRLADGYQM

PLQLNPNQEEVDHYGRHHHQQQQHSQAFFQPLECEPILQIGYQGQQDGMGAGPSVNNYML

GWLPYDTNSI

>AGL10

MGRGRVELKRIENKINRQVTFSKRRTGLLKKAQEISVLCDAEVSLIVFSHKGKLFEYSSE

SCMEKVLERYERYSYAERQLIAPDSHVNAQTNWSMEYSRLKAKIELLERNQRHYLGEELE

PMSLKDLQNLEQQLETALKHIRSRKNQLMNESLNHLQRKEKEIQEENSMLTKQIKERENI

LRTKQTQCEQLNRSVDDVPQPQPFQHPHLYMIAHQTSPFLNMGGLYQEEDQTAMRRNNLD

LTLEPIYNYLGCYAA

>AGL63

MRKGKRVIKKIEEKIKRQVTFAKRKKSLIKKAYELSVLCDVHLGLIIFSHSNRLYDFCSN

STSMENLIMRYQKEKEGQTTAEHSFHSCSDCVKTKESMMREIENLKLNLQLYDGHGLNLL

TYDELLSFELHLESSLQHARARKSEFMHQQQQQQTDQKLKGKEKGQGSSWEQLMWQAERQ

MMTCQRQKDPAPANEGGVPFLRWGTTHRRSSPP

>AGL7

MGRGRVQLKRIENKINRQVTFSKRRAGLLKKAHEISVLCDAEVALVVFSHKGKLFEYSTD

SCMEKILERYERYSYAERQLIAPESDVNTNWSMEYNRLKAKIELLERNQRHYLGEDLQAM

SPKELQNLEQQLDTALKHIRTRKNQLMYESINELQKKEKAIQEQNSMLSKQIKEREKILR

AQQEQWDQQNQGHNMPPPLPPQQHQIQHPYMLSHQPSPFLNMGGLYQEDDPMAMRRNDLE

LTLEPVYNCNLGCFAA

>AGL12

MARGKIQLKRIENPVHRQVTFCKRRTGLLKKAKELSVLCDAEIGVVIFSPQGKLFELATK

GTMEGMIDKYMKCTGGGRGSSSATFTAQEQLQPPNLDPKDEINVLKQEIEMLQKGISYMF

GGGDGAMNLEELLLLEKHLEYWISQIRSAKMDVMLQEIQSLRNKEGVLKNTNKYLLEKIE

ENNNSILDANFAVMETNYSYPLTMPSEIFQF

>AGL27

MGRRKIEIKRIENKSSRQVTFSKRRNGLIDKARQLSILCESSVAVVVVSASGKLYDSSSG

DEIEALFKPEKPQCFELDLEEKIQNYLPHKELLETVQSKLEEPNVDNVSVDSLISLEEQL

ETALSVSRARKAELMMEYIESLKEKEKLLREENQVLASQMGKNTLLATDDERGMFPGSSS

GNKIPETLPLLN

>AGL67

MGRVKLELKRIEKSTNRQITFSKRKKGLIKKAYELSTLCDIDLALLMFSPSDRLCLFSGQ

TRIEDVLARYINLPDQERENAIVFPDQSKRQGIQNKEYLLRTLEKLKIEDDMALQINEPR

PEATNSNVEELEQEVCRLQQQLQISEEELRKFEPDPMRLTSMEEIEACEANLINTLTRVV

QRREHLLRKSCEAQSNQQSMDGILLNDIVEDWGPEPEPKQAHMIANSAHHSNQPSYDLLL

RRSNSSSNQNPK

>AGL66

MGRVKLEIKRIENTTNRQVTFSKRRNGLIKKAYELSILCDIDIALLMFSPSDRLSLFSGK

TRIEDVFSRYINLSDQERENALVFPDQSRRPDFQSKEYLLRTLQQLKAENDIALQLTNPT

AINSDVEELEHEVYKLQQQLLMAEEELRKYEPDPIRFTTMEEYETCEKQLMDTLTRVNQR

REHILSQDQLSSYEASALQQQQSMGGPFGNDVVGGWLTENGPNEAHLFDASAHSAMYETL

LQGSSSSSNQNNIMGESNVSNHNGDMFQEWAQAYNSTTAHNPSTLFPPMQHQHGLVVDPN

IEEIEIPVMKKDAQADHEVSDYDIRMPQLSSQ

>AGL3

MGRGKVELKRIENKINRQVTFAKRRNGLLKKAYELSVLCDAEIALLIFSNRGKLYEFCSS

PSGMARTVDKYRKHSYATMDPNQSAKDLQDKYQDYLKLKSRVEILQHSQRHLLGEELSEM

DVNELEHLERQVDASLRQIRSTKARSMLDQLSDLKTKEEMLLETNRDLRRKLEDSDAALT

QSFWGSSAAEQQQQHQQQQQGMSSYQSNPPIQEAGFFKPLQGNVALQMSSHYNHNPANAT

NSATTSQNVNGFFPGWMV

>AGL44

MGRGKIVIRRIDNSTSRQVTFSKRRSGLLKKAKELSILCDAEVGVIIFSSTGKLYDYASN

SSMKTIIERYNRVKEEQHQLLNHASEIKFWQREVASLQQQLQYLQECHRKLVGEELSGMN

ANDLQNLEDQLVTSLKGVRLKKDQLMTNEIRELNRKGQIIQKENHELQNIVDIMRKENIK

LQKKVHGRTNAIEGNSSVDPISNGTTTYAPPQLQLIQLQPAPREKSIRLGLQLS

>AGL22

MAREKIQIRKIDNATARQVTFSKRRRGLFKKAEELSVLCDADVALIIFSSTGKLFEFCSS

SMKEVLERHNLQSKNLEKLDQPSLELQLVENSDHARMSKEIADKSHRLRQMRGEELQGLD

IEELQQLEKALETGLTRVIETKSDKIMSEISELQKKGMQLMDENKRLRQQGTQLTEENER

LGMQICNNVHAHGGAESENAAVYEEGQSSESITNAGNSTGAPVDSESSDTSLRLGLPYGG

>AGL17

MGRGKIVIQKIDDSTSRQVTFSKRRKGLIKKAKELAILCDAEVCLIIFSNTDKLYDFASS

SVKSTIERFNTAKMEEQELMNPASEVKFWQREAETLRQELHSLQENYRQLTGVELNGLSV

KELQNIESQLEMSLRGIRMKREQILTNEIKELTRKRNLVHHENLELSRKVQRIHQENVEL

YKKAYGTSNTNGLGHHELVDAVYESHAQVRLQLSQPEQSHYKTSSNS

>AGL5

MEGGASNEVAESSKKIGRGKIEIKRIENTTNRQVTFCKRRNGLLKKAYELSVLCDAEVAL

VIFSTRGRLYEYANNSVRGTIERYKKACSDAVNPPTITEANTQYYQQEASKLRRQIRDIQ

NLNRHILGESLGSLNFKELKNLESRLEKGISRVRSKKHEMLVAEIEYMQKREIELQNDNM

YLRSKITERTGLQQQESSVIHQGTVYESGVTSSHQSGQYNRNYIAVNLLEPNQNSSNQDQ

PPLQLV

>AGL6

MGRGRVEMKRIENKINRQVTFSKRRNGLLKKAYELSVLCDAEVALIIFSSRGKLYEFGSV

GIESTIERYNRCYNCSLSNNKPEETTQSWCQEVTKLKSKYESLVRTNRNLLGEDLGEMGV

KELQALERQLEAALTATRQRKTQVMMEEMEDLRKKERQLGDINKQLKIKFETEGHAFKTF

QDLWANSAASVAGDPNNSEFPVEPSHPNVLDCNTEPFLQIGFQQHYYVQGEGSSVSKSNV

AGETNFVQGWVL

>AGL20

MVRGKTQMKRIENATSRQVTFSKRRNGLLKKAFELSVLCDAEVSLIIFSPKGKLYEFASS

NMQDTIDRYLRHTKDRVSTKPVSEENMQHLKYEAANMMKKIEQLEASKRKLLGEGIGTCS

IEELQQIEQQLEKSVKCIRARKTQVFKEQIEQLKQKEKALAAENEKLSEKWGSHESEVWS

NKNQESTGRGDEESSPSSEVETQLFIGLPCSSRK

>AGL4

MGRGRVELKRIENKINRQVTFAKRRNGLLKKAYELSVLCDAEVSLIVFSNRGKLYEFCST

SNMLKTLERYQKCSYGSIEVNNKPAKELENSYREYLKLKGRYENLQRQQRNLLGEDLGPL

NSKELEQLERQLDGSLKQVRCIKTQYMLDQLSDLQGKEHILLDANRALSMKLEDMIGVRH

HHIGGGWEGGDQQNIAYGHPQAHSQGLYQSLECDPTLQIGYSHPVCSEQMAVTVQGQSQQ

GNGYIPGWML

>AGL79

MGRGRVQLRRIENKIRRQVTFSKRRTGLVKKAQEISVLCDAEVALIVFSPKGKLFEYSAG

SSMERILDRYERSAYAGQDIPTPNLDSQGECSTECSKLLRMIDVLQRSLRHLRGEEVDGL

SIRDLQGVEMQLDTALKKTRSRKNQLMVESIAQLQKKEKELKELKKQLTKKAGEREDFQT

QNLSHDLASLATPPFESPHELRRTISPPPPPLSSGDTSQRDGVGEVAAGTLIRRTNATLP

HWMPQLTGE

>AP3

MARGKIQIKRIENQTNRQVTYSKRRNGLFKKAHELTVLCDARVSIIMFSSSNKLHEYISP

NTTTKEIVDLYQTISDVDVWATQYERMQETKRKLLETNRNLRTQIKQRLGECLDELDIQE

LRRLEDEMENTFKLVRERKFKSLGNQIETTKKKNKSQQDIQKNLIHELELRAEDPHYGLV

DNGGDYDSVLGYQIEGSRAYALRFHQNHHHYYPNHGLHAPSASDIITFHLLE

>AGL16

MGRGKIAIKRINNSTSRQVTFSKRRNGLLKKAKELAILCDAEVGVIIFSSTGRLYDFSSS

SMKSVIERYSDAKGETSSENDPASEIQFWQKEAAILKRQLHNLQENHRQMMGEELSGLSV

EALQNLENQLELSLRGVRMKKDQMLIEEIQVLNREGNLVHQENLDLHKKVNLMHQQNMEL

HEKVSEVEGVKIANKNSLLTNGLDMRDTSNEHVHLQLSQPQHDHETHSKAIQLNYFSFIA

>AGL18

MGRGRIEIKKIENINSRQVTFSKRRNGLIKKAKELSILCDAEVALIIFSSTGKIYDFSSV

CMEQILSRYGYTTASTEHKQQREHQLLICASHGNEAVLRNDDSMKGELERLQLAIERLKG

KELEGMSFPDLISLENQLNESLHSVKDQKTQILLNQIERSRIQEKKALEENQILRKQVEM

LGRGSGPKVLNERPQDSSPEADPESSSSEEDENDNEEHHSDTSLQLGLSSTGYCTKRKKP

KIELVCDNSGSQVASD

>AGL1

MEEGGSSHDAESSKKLGRGKIEIKRIENTTNRQVTFCKRRNGLLKKAYELSVLCDAEVAL

VIFSTRGRLYEYANNSVRGTIERYKKACSDAVNPPSVTEANTQYYQQEASKLRRQIRDIQ

NSNRHIVGESLGSLNFKELKNLEGRLEKGISRVRSKKNELLVAEIEYMQKREMELQHNNM

YLRAKIAEGARLNPDQQESSVIQGTTVYESGVSSHDQSQHYNRNYIPVNLLEPNQQFSGQ

DQPPLQLV

>AGL13

MGRGKVEVKRIENKITRQVTFSKRKSGLLKKAYELSVLCDAEVSLIIFSTGGKLYEFSNV

GVGRTIERYYRCKDNLLDNDTLEDTQGLRQEVTKLKCKYESLLRTHRNLVGEDLEGMSIK

ELQTLERQLEGALSATRKQKTQVMMEQMEELRRKERELGDINNKLKLETEDHDFKGFQDL

LLNPVLTAGCSTDFSLQSTHQNYISDCNLGYFLQIGFQQHYEQGEGSSVTKSNARSDAET

NFVQ

>AGL11

MGRGKIEIKRIENSTNRQVTFCKRRNGLLKKAYELSVLCDAEVALIVFSTRGRLYEYANN

NIRSTIERYKKACSDSTNTSTVQEINAAYYQQESAKLRQQIQTIQNSNRNLMGDSLSSLS

VKELKQVENRLEKAISRIRSKKHELLLVEIENAQKREIELDNENIYLRTKVAEVERYQQH

HHQMVSGSEINAIEALASRNYFAHSIMTAGSGSGNGGSYSDPDKKILHLG

>AGL14

MVRGKTEMKRIENATSRQVTFSKRRNGLLKKAFELSVLCDAEVALIIFSPRGKLYEFSSS

SSIPKTVERYQKRIQDLGSNHKRNDNSQQSKDETYGLARKIEHLEISTRKMMGEGLDASS

IEELQQLENQLDRSLMKIRAKKYQLLREETEKLKEKERNLIAENKMLMEKCEMQGRGIIG

RISSSSSTSELDIDDNEMEVVTDLFIGPPETRHFKKFPPSN

>AG

TAYQSELGGDSSPLRKSGRGKIEIKRIENTTNRQVTFCKRRNGLLKKAYELSVLCDAEVA

LIVFSSRGRLYEYSNNSVKGTIERYKKAISDNSNTGSVAEINAQYYQQESAKLRQQIISI

QNSNRQLMGETIGSMSPKELRNLEGRLERSITRIRSKKNELLFSEIDYMQKREVDLHNDN

QILRAKIAENERNNPSISLMPGGSNYEQLMPPPQTQSQPFDSRNYFQVAALQPNNHHYSS

AGRQDQTALQLV

>AGL19

MVRGKTEMKRIENATSRQVTFSKRRNGLLKKAFELSVLCDAEVALVIFSPRSKLYEFSSS

SIAATIERYQRRIKEIGNNHKRNDNSQQARDETSGLTKKIEQLEISKRKLLGEGIDACSI

EELQQLENQLDRSLSRIRAKKYQLLREEIEKLKAEERNLVKENKDLKEKWLGMGTATIAS

SQSTLSSSEVNIDDNMEVETGLFIGPPETRQSKKFPPQN

>AGL24

MAREKIRIKKIDNITARQVTFSKRRRGIFKKADELSVLCDADVALIIFSATGKLFEFSSS

RMRDILGRYSLHASNINKLMDPPSTHLRLENCNLSRLSKEVEDKTKQLRKLRGEDLDGLN

LEELQRLEKLLESGLSRVSEKKGECVMSQIFSLEKRGSELVDENKRLRDKLETLERAKLT

TLKEALETESVTTNVSSYDSGTPLEDDSDTSLKLGLPSWE

>AGL21

MGRGKIVIQRIDDSTSRQVTFSKRRKGLIKKAKELAILCDAEVGLIIFSSTGKLYDFASS

SMKSVIDRYNKSKIEQQQLLNPASEVKFWQREAAVLRQELHALQENHRQMMGEQLNGLSV

NELNSLENQIEISLRGIRMRKEQLLTQEIQELSQKRNLIHQENLDLSRKVQRIHQENVEL

YKKAYMANTNGFTHREVAVADDESHTQIRLQLSQPEHSDYDTPPRANE

>AGL25

MGRKKLEIKRIENKSSRQVTFSKRRNGLIEKARQLSVLCDASVALLVVSASGKLYSFSSG

DNLVKILDRYGKQHADDLKALDHQSKALNYGSHYELLELVDSKLVGSNVKNVSIDALVQL

EEHLETALSVTRAKKTELMLKLVENLKEKEKMLKEENQVLASQMENNHHVGAEAEMEMSP

AGQISDNLPVTLPLLN

>AGL15

MGRGKIEIKRIENANSRQVTFSKRRSGLLKKARELSVLCDAEVAVIVFSKSGKLFEYSST

GMKQTLSRYGNHQSSSASKAEEDCAEVDILKDQLSKLQEKHLQLQGKGLNPLTFKELQSL

EQQLYHALITVRERKERLLTNQLEESRLKEQRAELENETLRRQVQELRSFLPSFTHYVPS

YIKCFAIDPKNALINHDSKCSLQNTDSDTTLQLGLPGEAHDRRTNEGERESPSSDSVTTN

TSSETAERGDQSSLANSPPEAKRQRFSV

>AGL2

MGRGRVELKRIENKINRQVTFAKRRNGLLKKAYELSVLCDAEVALIIFSNRGKLYEFCSS

SNMLKTLDRYQKCSYGSIEVNNKPAKELENSYREYLKLKGRYENLQRQQRNLLGEDLGPL

NSKELEQLERQLDGSLKQVRSIKTQYMLDQLSDLQNKEQMLLETNRALAMKLDDMIGVRS

HHMGGGGGWEGGEQNVTYAHHQAQSQGLYQPLECNPTLQMGYDNPVCSEQITATTQAQAQ

QGNGYIPGWML

>PI

MGRGKIEIKRIENANNRVVTFSKRRNGLVKKAKEITVLCDAKVALIIFASNGKMIDYCCP

SMDLGAMLDQYQKLSGKKLWDAKHENLSNEIDRIKKENDSLQLELRHLKGEDIQSLNLKN

LMAVEHAIEHGLDKVRDHQMEILISKRRNEKMMAEEQRQLTFQLQQQEMAIASNARGMMM

RDHDGQFGYRVQPIQPNLQEKIMSLVID

>AGL32

MGRGKIEIKKIENQTARQVTFSKRRTGLIKKTRELSILCDAHIGLIVFSATGKLSEFCSE

QNRMPQLIDRYLHTNGLRLPDHHDDQEQLHHEMELLRRETCNLELRLRPFHGHDLASIPP

NELDGLERQLEHSVLKVRERKQQLENLSRKRRMLEEDNNNMYRWLHEHRAAMEFQQAGID

TKPGEYQQFIEQLQCYKPGEYQQFLEQQQQQPNSVLQLATLPSEIDPTYNLQLAQPNLQN

DPTAQND

>AGL72

MVRGKIEIKKIENVTSRQVTFSKRRSGLFKKAHELSVLCDAQVAAMIFSQKGRLYEFASS

DIRNTIKRYAEYKREYFVAETHPIEQYVQGLKKEMVTMVKKIEVLEVHNRKMMGQSLDSC

SVKELSEIATQIEKSLHMVRLRKAKLYEDELQKLKAKERELKDERVRLSLKKTIYTHLCQ

VGERPMGMPSGSKEKEDVETDLFIGFLKNRP

>AGL71

MVRGKIEIKKIENVTSRQVTFSKRRSGLFKKAHELSVLCDAQVAAIVFSQSGRLHEYSSS

QMEKIIDRYGKFSNAFYVAERPQVERYLQELKMEIDRMVKKIDLLEVHHRKLLGQGLDSC

SVTELQEIDTQIEKSLRIVRSRKAELYADQLKKLKEKERELLNERKRLLEEVNMHHSSKG

NTEGGHRTKHSSEVETDLFIGLPVTRL

>AGL8

MGRGRVQLKRIENKINRQVTFSKRRSGLLKKAHEISVLCDAEVALIVFSSKGKLFEYSTD

SCMERILERYDRYLYSDKQLVGRDVSQSENWVLEHAKLKARVEVLEKNKRNFMGEDLDSL

SLKELQSLEHQLDAAIKSIRSRKNQAMFESISALQKKDKALQDHNNSLLKKIKEREKKTG

QQEGQLVQCSNSSSVLLPQYCVTSSRDGFVERVGGENGGASSLTEPNSLLPAWMLRPTTT

NE

>AGL42

MVRGKIEMKKIENATSRQVTFSKRRNGLLKKAYELSVLCDAQLSLIIFSQRGRLYEFSSS

DMQKTIERYRKYTKDHETSNHDSQIHLQQLKQEASHMITKIELLEFHKRKLLGQGIASCS

LEELQEIDSQLQRSLGKVRERKAQLFKEQLEKLKAKEKQLLEENVKLHQKNVINPWRGSS

TDQQQEKYKVIDLNLEVETDLFIGLPNRNC

>AGL31

MGRKKVEIKRIENKSSRQVTFSKRRNGLIEKARQLSILCESSIAVLVVSGSGKLYKSASG

DNMSKIIDRYEIHHADELEALDLAEKTRNYLPLKELLEIVQSKLEESNVDNASVDTLISL

EEQLETALSVTRARKTELMMGEVKSLQKTVGKKTFLVIEGDRGMSWENGSGNKVRETLPL

LK

>AGL70

MGRRKVEIKRIENKSSRQVTFSKRRKGLIEKARQLSILCESSIAVVAVSGSGKLYDSASG

DNMSKIIDRYEIHHADELKALDLAEKIRNYLPHKELLEIVQSKLEESNVDNVSVDSLISM

EEQLETALSVIRAKKTELMMEDMKSLQEREKLLIEENQILASQVGKKTFLVIEGDRGMSR

ENGSGNKVPETLSLLK

>AGL69

MGRRKVEIKRIENKSSRQVTFCKRRNGLMEKARQLSILCESSVALIIISATGRLYSFSSG

DSMAKILSRYELEQADDLKTLDLEEKTLNYLSHKELLETIQCKIEEAKSDNVSIDCLKSL

EEQLKTALSVTRARKTELMMELVKTHQEKEKLLREENQSLTNQLIKMGKMKKSVEAEDAR

AMSPESSSDNKPPETLLLLK

>AGL68

MCRKSEAMGRRRVEIKRIENKSSRQVTFCKRRNGLMEKARQLSILCGSSVALFIVSSTGK

LYNSSSGDSMAKIISRFKIQQADDPETLDLEDKTQDYLSHKELLEIVQRKIEEAKGDNVS

IESLISMEEQLKSALSVIRARKTELLMELVKNLQDKEKLLKEKNKVLASEVGKLKKILET

GDERAVMSPENSSGHSPPETLPLLK

>ZaMADS105

MKRTLSRYNKCPDFSEAPVAEYETEKQDSEEVDGLKDTIAKLQTKQFRQLLGKDLNGLSLKELQLLEQELDEGFLLVKEKKEQLLMEQLEQSRVQEQRAMLENETLRRQVEELRGFFPSTECSVPAYLEYYPLGRKNSLMNQSSASPDVASDSA

>ZaMADS104

MGRGKIEIKRIENSNSRQVTFSKRRAGMLKKAQELATLCDAEVAVIIFSNTGKLFEFSSSGMRRTLSRYNKCLDISEAAVVEYKTEKQDSKEVDGLKDEIAKLQTKQSRLLGKDLNDLRLKELRLLEQELNEGLLFVRAKKEQLLMEQLEQSRVQEQRAMLENETLRRQVEELRGFFPSTECLLPAYIEYCPLGRKNSLMNHSSASPDNASDSAVEKGDSDTTLHLGLPSNICHKRKAPEGETNSNDSRRHLGLL

>ZaMADS100

MENPTTRQVTFSKRRAGLLKKTHELSVLCGLIIFSSTAKLFQYCTHPFRMEHIIERRLKVTGTRIPEHDSREQLFNELAVLRKETRRLQLSMRRYTGEDLVSIPYEDLDELEHELESSVHKVRERKQLDNLRRKIEDQRAALEYQQWKQSQWSNLQDPFFGDHQQQPSSLLQLALNNIPPQIHPYNHHHLQLAQPNLQDPNF*

>ZaMADS38

MGRGRVELKRIENKINRQVTFAKRRNGLLKKAYELSVLCDAEVALIIFSNRGKLYEFCSSSSMLKTLERYQKCSYGAVEVNKPAKELESSYREYLKLKTRFEALQRAQRNLLGEDLGPLSSKELEQLERQLDSSLKHVRSTRTQFMLDQLSDLQNKEQLLLDANRALSMKLDEINAKTQLQPSWEGNEQHMAYNPLTQGLFQPIECNPTLQIGYNPSSCSDQMTATTHTQQVSGFIPGWML*

>ZaMADS101

MVRGKTQMKLIENATSRQVTFSKRRNGLLKKACELSVLCEAEVALIIFSPRGKLYEFSSSSMQETIGRYLRHTKDTRNKQQLTEQQMEHLKHEAADMVKKMELLEVSKQKLLGEDLSSCTLEELQQMELQLDRSVSNIRARKNQVFKEQIEQLNEKGKVLEAENSRLAEKCGTEQLQGSKEQQENLPNDDNGTPTSDVETELFIGPPPERRTRCLLFPPHN*

>ZaMADS43

MDTLCYGGISISAGAAFRALSMLKTLERYQKCNYGAPEPNVSAREALELSSQQEYLKLKARYEALQRSQRNLLGEELGPLNSKELESLERQLDMSLKQIRSIRTQYMLDTLTDLQHKEQLMSEANKTLKQRLMEGYQLNTLQLNPSAEDCGYGLKQVHPQCDDTFFQPLDCEPTLQIGYQAADPASVVIAGPSINNYMHGWLPC*

>ZaMADS35

MGRGRVQMKRIENKISRQVTFSKRRAGLLKKAHEISVLCDAEVALIVFSTKGKLFEYSTDSSMERILERYEKNAYVEQQLLSTDAGLQGSWSLEYHKLKNRIEVLERNIRNFMGEDLEPLGVRELQNLEQQIDTALKRIRTRENQLMHESISDLQKKARALQDQNNMLAKQLKEKEETVTEQQTQMGQNSSSSMPPTPPVLTFPSQTTGGSFQMIRGTEIDEPEHIQTRPNMSSIVVPSWMLGHVNHHE*

>ZaMADS65

MAREKIKIKKIDNVTARQVTFSKRRRGLFKKAEELAVLCDADVALIIFSATGKHFEYSSSSMKEILEKHHMHSKNLERLDRPSLDLQLMKDDNYSRLSKEVSEKSHQLRQMRGEEIHGLSLEELQELERSLEVGLGRVMDKKGEKILKEINEFKRRGLLLMEENERLRQRVAEVTNAERQTAPDSDNLNSEEGQSTESVNFVCNSSNGPPPESESSDISLKLGLPYAG*

>ZaMADS81

MGRGKIEIKRIENLNNRHVTYSKRRNGLIKKTKEIAVLCDAKAVVIIVPLSGKIHEYCSAPISEILEGYQKKSSKKLWDDKHEKLSNEIDRIKKENDIMQIKLRHLKGQDVTSLNIKELMSFEDSLENGLTGISNKQARTY*

>ZaMADS102

MLHSCHINRNLLGEELGPLNSKELESLERQLDMSLKQIRSIRVRGLNKLSLLWNHIVRSWLLKMHVVTTLCSLLGYCTIKTT*

>ZaMADS36

MTKSLERYQSCSYGSLQANQSAKETQANYEEYIKLKEKQEALQRSQKQFSGEDLGDLALKELQQLERQLDSSLGKVRSLKARNQLDKLSELQRKVYMRL*

>ZaMADS103

MVRGKIEMKKIENATSRQVTFSKRRNGLLKKAFELSVLCDAEVAAIIFSQKGRIYEFSSSELHDTIERYYKYAKVVQSEQPGMEQYKQQLTEEIANIVEKIQQIEVSQRKLLGKDLDSCTIEELQELEGQLERSLRSIRDR

>ZaMADS84

MGRGRVQLKRIENKISRQVTFSKRRGGLLKKAHEISVLCDAEVALIVFSTKGKLFEYSTDSRMERILERYERNTYVEQQLGTTDTQQQGCWSLEYPLLKNRIEVIERNIRNFMGEDLEPLGLRELQHLEQQIDTSLKRIRTRKNQLINESISDLHKRERALQDQNNTLAKKLKEKEKTVLTEQQNQMGQNSSPFMPTPP

>ZaMADS80

MGRGKIEIKRIENTTNRQVTFCKRRNGLLKKAYELSVLCDAEVALIVFSSRGRLYEYSNSNSIRSTVDRYKKACSDNSNSGSVTEINAQYYQQESAKLRQQIQMIQNSNRHLMGDSLSSLTVKELKQLENRLERGITRIRSKKHEMLLAEIEFLQKREIELENESVCLRSKIAEVERFQQANMVTGQELNAIQALASRNFFSPIIEGGTTYSHPDKKTLHLG

>ZaMADS62

MGRNSLPFSATPSRIFIPLYSSFQAAAIMEFNPNNQNLESGSSHNKKMGRGKIEIKRIENTTNRQVTFCKRRNGLLKKAYELSVLCDAEVALIVFSNRGRLYEYANNSVRATIERYKKACTGSSNPGSVTEANTQFYQQEATKLRRQIREIQNSNRHILGEALSSLSFKELKSLETRLEKGISRVRCKKNEMLLAEVEFMQKREIQLQNDNMYLRARIAENERVEQEQQSESMMQGGGPVYESVASQPYDRNFFPVNLLEPNHQYSRQDDQTPLQLV*

>ZaMADS60

MGRGRVELKRIENKINRQVTFSKRRNGLLKKAYELSVLCDAEVALIIFSSSGKLYEFCSSSSMTKSLERYQSCSYGSLQANQSAKETQANYEEYIKLKEKQEALQRSQKQFSGEDLGDLALKELQQLERQLDSSLGKVRSLKARNQLDKLSELQRKEEMLLETNKTLSKKLEEIDTVLRSRSWETGQEQSTTYNNQPSHSQGSFGPSHFNNPFHIGYDPGVTDYSRTVTTADQQNANGLIPEWML*

>ZaMADS55

MGRGKIEIKKIENANSRQVTFSKRRAGLLKKAKELAILCDAEVAVIIFSNTGKLFEYSSSGMKRTLSRYNKCPDFSEAPVAEYETEKQDSKEVDGLKDTIAKLQTKQLQLLGKDLNGLGLKELQLLEQELDEGFLLVKEKKEQLLMEQLEQSRVQEQRAMLENETLRRQVEELRGFFPSTECSVPAYLEYYPLGRKNSLMNHSSASPDVASDSAIEKGDSDTTLHLGLPSNIYHKTKAPEGESHSNDSRSQLGLL*

>ZaMADS49

MIVGTNRILPKYSGSMGRGKIEIKRIENTTNRQVTFCKRRNGLLKKAYELSVLCDAEVALIVFSSRGRLYEYSNSNIRSTIDRYKKTCSDNSNSGSVTEINAQYYQQESAKLRQQIQMLQNSNRNLMGESVSSLTVKELKQLENRLERGITRIRSKKHEMLLAEIEFFQKREIELENESLSLRSKIAEVERFQQANMVNGEELNAIHALASRNLFSPLMEGGSATAYSHPHPHTEKKMLHLG*

>ZaMADS77

MKNILFVLAASLKMVRGKTQMRRIENATSRQVTFSKRRNGLLKKAYELSVLCDAEVAIIIFSPRGKLYEFANSSMQETVERYLRHTKDTRTKQQPTEKHMQQHLKHEAASMAKKIELLEVSKRKLLGEGLASSTLEELQQIEHQLE

>ZaMADS85

RIENKISRQVTFSKRRGGLLKKAHEISVLCDAQVALIVFSTKGKLFEYSTDSSMERILERYERKSYVEQLVATDTELQGCWSLEYPKLKSKTEVLDRNIRNIMGEDLEPLGLRELQHLEQQIDTALKRIRTREKQLIHKSISDLQKMERALQDQNNMLEKKLKEKEKTVADEQQNQMEQNSLPSMPTPPLMLTFPSPNIG

>ZaMADS68

ELSVLCEAEVALIIFSPRGKLYEFSSSSMQETIGRYLRHTKDTRNKQQLTEQQMEHLKHEAADMVKKMELLEVSKQKLLGEDLSSCTLEELQQMELQLDRSVSNIRARKVHLIRTITANPSKDFVTCAKHLSMFQNQVFKEQIEQLNEKVRPIKIYNVVRYGITPFLLSPYSTTLFEDTSFLDSKKKERKRDTVLSIFMSRSMLGL

>ZaMADS63

KRIENLNNRHVTYSKRRNGLIKKTKEIAVLCDAKAVVIIVPLSGKIHEYCSTPISEILEGYQKKSSKKLWDDKHEKLSNEIDRIKKENDIMQIKLRHLNGQDVTSLNIKELMSFEDSLENGLTGISNKQSKLIERMRKNGKMLEEENNYLKFILRQQEIAKQQMAMKNNAREIENGYHQQRENHEYNLHMPLTFNMKPI

>ZaMADS44

MGRGKIEIARIESRTNRQVTFSKRRGGLLKKAHELSVLCDAQIGLIIFSCSGKLTEFCSDSTSIDQIIRKYEAAKGTRINPADINNHDDTEEIYSELRRMRNETHNLELSLGRYTGDIDLNSVQFEELALLEDQLECSMNKVRARKMEILRLKTDDLRRKEKVLEDENEQILNLIKDNNQMAWEQQQATLMASKIEEHGHVLDQFPFSGEPQPSSVLELAINPQYLQPTQPNLQDFSLHQFTNYE*

>ZaMADS51

MEMIKKKPCVGRQKIAISKIPKKNHLQVTFSKRRAGVFKKASELSTLCGVDIALIVFSPANKPFSFGHPNVDSIVDRFLTQNPNPNPNQAPAGSGTNRLIEAHRNANIRELNLQLTQVLHQLEVEKKHGEVLSEIRKASQSQCWWEAPVNELGLHELEQLKTAMEELKKNVETQANKILIESKNNPSGPSPFFGVNYQTMNPHHESKLHLDQIHASSNNVHPNYNFYSLAYGQCHDHHQQLL*

>ZaMADS47

MVRGKTQMKLIENATSRQVTFSKRRNGLLKKAYELSVLCDAEVALIIFSSRGKHYEFASSSMQETIERYLKYTIDTRSKQQPTEQHIQNLKHEAANMVKKIELLEVSKQKLLGEGLAECTLEELQQIKHQLERGVSAVRARKNQIFKERIEQLKEKGRVLEAGNARLAEKCGIEFEQLHGLKERQENIPNDDIGTPTSDVETELFIGPPPERRTRRLPMPRHK*

>ZaMADS53

MGRGRVELKRIENKINRQVTFAKRRNGLLKKAYELSVLCDAEVALIIFSNRGKLYEFCSSPSMMKTLERYHRCNFGALEANRPPSETQQSTYQECLRLKTTVDVLQQTQRNLLGEDLGPLSTKELEQLEHQLETSLQQVRSTKTQFMVDQLTDLQKREHVLLQVNAGLRKKLEESNAHQFHHRLAWEAAAAAAAAAGQNMTYNGYPVQSEGFFQPLGGNPTLQIGYNPVGSEEVNDPVHAHVNGFVPGWML*

>ZaMADS69

MGRGKVELKRIENKINRQVTFSKRRNGVLKKAYELSVLCDAEVALIIFSSRGKLYEFGSAGVSKTLERYQRCCFNPQDNNSIEHETQSWYQEVTKLKAKYESLQRTQRHLLGEDLAPLSVKELQNLEKQLEGALSMARQRKVTYLYSCMFNQYGSI*

>ZaMADS71

MTRKKVKLMWIVNDSARKASLKKRRAGLLKKVSELTTLCDVNAFILIYSPGEREPEMWPSRPVVEQLLTRFNNVPEMERSKKMMNQETYLKERVGKVQDQVKKNSRKNKELDVSHLMEQVSHGKTIDDFNINELQGLVWYTEERKKDIRKKIEYYQQVNPLPPESLPLPPPPPPQHPSPVDSTAGVGGSTGGDGRNPPQPESALWDQWFIDMINSSENNAGSSSAGAKSDAGLTSHQAFDACSGAASAANQMGLPHGYPEAYNTDSDKGLPQGNINIGSSSSLGGNDGNMGQPQGNAGEASNAADGAMGLPSELFGGSIAGSDIEIPYDVTKQWPGNFHT*

>ZaMADS73

MTRKKVKLAFISNDSARKATFKKRKKGLLKKVSELSTLCGIDACAIIFSPYDSQPEIWPSPLGVKRVLSQFKKMPEMEQSKKMVNQDSFLRQRIGKANEQLKKQRKDNREKEMTQVMFQSLSGKALLSLNMMDLNDLVGWLIEQNLKEICKRIWDLVVKKLCTHMGKAAAKTLFLA*

>ZaMADS75

MGRGKIEIKRIENPTNRQVTYSKRRNGIFKKAEELTVLCDAKVSLILLSNSGKVHEYISPSTTTKKMFDQYQQSLGVDLWSTHYDKMQETYRKLKEINNKLKKDIRQRMGEDLEDVTSEQLQGLEQNMTTSLATILERKVLSLSISQLDLNES*

>ZaMADS76

MVRGKIQMKKIENATSRQVTFSKRKNGLLKKAYELSVLCDAEVAAIIFSQKGTLYEFSSSGMQHTMERYYNYTKEKEEQSDLPGMEQHMQQLKHEITKMIEKIEHIEVSQRKLLGQNLDSCTIEELGELDSQLGRSLRSIRARKACNSLSQQKLFCHFLRRNPCSAIQ*

>ZaMADS82

MVRGKTQMKRIENTTSRQVTFSKRRNGLLKKAFELSVLCDAEVALIIFSPRGKLYEFSSCSINKTIERYQKRSKDFGINTKIVEEDHTQEETFNMVKELEFLEVTKRKLLGGGLEPCSIDELQQLENQLERSLSRIRARKNQLFREQIEKLKEKVNLLCIYLPF*

>ZaMADS64

MGRGKIAIKKMENPTTRQVTFSKRRAGLLKKTHELSVLSDAQSGLIIFSSTAKLFQYCTHPFRMEHIIERRLKVTGTRIPEHDSREQLLNELAVLRKETRRLQLSMRRYTGEDLVSIPYEDLDELEHELESSVHKVRERKQLDNLRRKIEDQRAALEYQQWKQSQWSNLQDPFFGDHQQQPSSLLQLALNNIPPQIHPYNHHHLQLAQPNLQDPNF*

>ZaMADS52

MTRQKIEIKKIENPAARQVTFSKRRRGLFKKAQELSTLCDAELALIVFSATGKLFDYSSAGSSMKQVIERHSLHSQNLHKFDQSSLELQLEGSTYAILRKEIAERTLELRRMRGEELQELDMEQVKRLEKSLEDGLSRVVQTKGEKILKQIDALRTKESQLMEENLRLKQQSGNNIQAEGNPFESIIKITSSPDKDNSDISLKLGLPFPSK*

>ZaMADS46

MVRGKTQMKRIENPTSRQVTFSKRRNGLLKKAFELSVLCDAEIALIIFSPRGKLYEFSSCSINKTIGQYQKRSKDVGISTKRVEEDHTQYLKEETFNMVKKLEFLEVTKRKLLGDGLEPCSIDELQQLECQLERSISRIRARKNQLFREQIEELKEKEKILMEENIKLWEKCGMLPRQASAEQEILNIQSMDVETELFIGPPERRVTLHKAYKKNP*

>ZaMADS88

MISNLLLLLLCLVMEAKTTSSLHHSLITMPKKKPKGTGRKKIEIKKIENNSSLKVAFSKRRKGMFKKASELCRLCGAEIAVIVFSPKGRPYSFGDRVIDKFMAENDETPAVIDRSEEQMNEDNSSEEMHEELGFWWEQSIDNMDLEEIEKYKSCLEELRYNVAEKIEEMVMRRTCERDFLGVSHDII*

>ZaMADS89

MAREKIKIRKIDNITARQVTFSKRRRGLFKKAEELSVLCDAEVAVIIFSATGKLFENSSSSMKDILARYNVHSSNIGKLNQPSLELQLENSKHTSLSKEVTDKSNQLRQMRGEDLNGLNIEELQRLEKMLELGLSRVLETKDNKFNSEISSLERKGAKLLEENTHLKQKMASMSKGKRLALVDSDVGTREEGMSSDSVNNVCSCSGGPPPEDDSSDTSLKLGLPFSN*

>ZaMADS83

MARGKVQMKRIENPVHRQVTFCKRRSGLLKKAKELSILCDADIGVFIFSNHGKLYELATKGTMQGLIERYLKSTLGAQAEPAVIQNQQLDAMEEINMLKKEIEILQKGLRYMFGGGAETMTLDELLVIEKHLELWIYNIRSAKMDIMIQEINLLRNKEGILTAANNYLQEKVDENTGVANFGPMTTNIEYPLTIPNDIFQF*

>ZaMADS74

MGRGKVLLERIQNKINRQVTFSKRRNGLLKKAYELSLLCDAEVALIIFSSHGKLFEFGSNDGVEKILERYRQCCYSSQNPSNINELEPEGLFPEILRLRALRESLERSQRYFLGEDLGTLGVKELLKLEKQLDKTISLSRLRKSELMRQELENLQQKTHDLGEENKQLKAKLEKSIQELGADPNYIAMASNCFRVHPSQHIG*

>ZaMADS58

MTRQKIEIKKIENPIARQVTFSKRRKGLFKKAQELSTLCDAELALIVFSATGKLFDYSSSSSSMKQLIERHNLHSQNLHQPSLELQLESSTCAILSKEMAERTRELRQMRGEELQELNMEELERLEKSLEVGLSHVVQTKGERVLKEIDALRTKQGQLMEENRRLKQQQTGNAHVQGHSFESVTFISSSGDNSQDKDSSDTSLKLGLVH*

>ZaMADS45

MGRGTVELKRIESKANRQVTFSKRKNGILKKALELSILCDAEIALIIFSPSGKPYRYASDNNNPERIIARYRREVGLLSNSANHESFGLMQFWKSEIDQLERSVEAMEARLRHLAGEDISSLGVKELKILEGQLKIGAERICTRTSRLLAEKINELKTKQRELQENNSRLQKIVRLQILSLYVGQVFI*

>ZaMADS61

MTGIGHKKTQMKMSQGSDARQVAPSRRRSGLFKRASELTTLYAVETALVIFSPGDKAVPYAHPGAEPVIINLAPTGMPDSGLTQCAQADHEATMRALNKEYHDLLEQLEAEKKRGEKLQERKMMNQQRYGRRLWDIPVDELNLEELLKLKAIMEDLDEKLQKHMAKHSSQAYTPTEGCSVDPNGHGTGPGN*

>ZaMADS57

MNEELTSIFCNFQLLLNKTKKKGSQLVKIENIYFQAAAAGAEAIASMEFPNNENPESSSHNKKMGRGKIEIKRIENTTNRQVTFCKRRNGLLKKAYELSVLCDAEVSLIVFSSRGRLYEYANNSVRATIDRYKKACADSSNPGSVTEANTQFYQQESTKLRRQIREIQNFNRHILGEALSSLSFKELKNLETRLEKGISKVRSKKNEMLLAEIEFMQKREIQLQNDNMYLRARIAENERAEQEQQSESMMHGRGPVYESAASQPYDRNFLPVNLLEPDHQYSRQGDQLPLQLV*

>ZaMADS90

MTRKKVKLAFISNDSARKATFKKRKKGLLKKVSELSTLCGIDACAIIFSPYDSQPEIWPSPLGVQRVLSQFKKMPEMEQSKKMVNQDSFLRQRIGKANEQLKKQRKDNREKEMTQVMFQSLTGKTLLSLNMMDLNDLGWLIEQDLKEICKRIETLKNNIATHSSNVVVRNNGEISRHVEKSSGVDQMNMEAMQKQQWFLDLMNPQEHMGFGGEEIMYPFGESSSHNTLWPNPFYP*

>ZaMADS48

MARGKIQIKRIENSTNRQVTFSKRRNGLFKKARELTVLCDAKVSIIMCSSSGKVYEYISPLTTTKHLLDEYQKTLKIDIWSSKYEKMQEDLKHVKEVNENLRKEIGQRLGESLNDLSLVELYGLQQDIDNSLKIIREQKDKVLSGQINTWKRKVKSVEQQHKNLQSGFIINAKEEDPNYDLVDNGGHYDTVIGLRNEGPGIFIRLQPN*

>ZaMADS96

MVRGKIEMKKIENATSRQVTFSKRRNGLLKKAFELSVLCDAEVAAIIFSKKGRIYEFSNSEMHDTIERYYKYAKEVQSDQPGMEQYKQQLMEGIANMVEKIEQIEVSQRKLLGKDLDSCTIEELQELEGQLERSLRSIRDRKAQLLQEQINQLKEKERLLREENVSLCTMVNISSLLDR*

>ZaMADS92

MGRGKIEIKRIENLNNRHVTYSKRRNGLIKKTKEIAVLCDAKAIVIIVPTSGKIHEYCSAPISEILEGYQKKSSKKLWDDKHEKLSNEIDRIKKENDIMQIKLRHLKGQDVTSLNIKELMSFEDSLENGLTGISNKQSKLIERMRKNGKMLEEENNYLKFILRQQEIVKQQQQMAMENNAREIENGYHQKRENHEYNLHMPLTFNMQPI*

>ZaMADS72

MTRKKVKLTWIVNDSARKASLKKRRVGLLKKVSELTTLCGVNAFVLIYSPEEREPQMWPSRPVVEQLLARFNNMPEMEKCKKMMNLETYLKERVGKVQDQLKKHSRKNKEMDVYQIMEQVHHGKPTDDFNINELHGLIWFSEEKKKEIRKRIEYYRQINPLPLESLPPPSPPPQLPAPMDGMAGVGGSIGGDGRNPPESGLWDQWFIDMVNNSENVAGSSSAKAKSDAGLTSNQAFAASSGAANQMGLPHGNPRAYNIGSAMGMPQGNFNIGSSSSSLGGNNGGFDGNMGQPQENAGADSNAAGRELGLQYELFGGSIAGSDVGMPYDVSKQWPGNFYP*

>ZaMADS50

MGRGRVELKRIENKINRQVTFAKRRNGLLKKAYELSVLCDAEVALIIFSNRGKLYEFCSSASMLKTLERYQKCSYGAVEVNKPAKELESSYREYLKLKTKFEALQRTQRNLLGEDLGPLNSKELEQLERQLESSLKHVRSTKTQFILDQLSDLQNKEQMLLDANRALTIKLDEINAKTQLRHSWEGSEHQMAYNPQHAETQGLFQPIECNPTLQIGYNPSCSDQMTATTHGQQVSGFIPGWML*

>ZaMADS99

MDKTEATSSIKKKPCGSGRRKVEIKKIESSSSRMVAFSKRKKGIFNKGCELYRLCDADIAVVIFSSTGRPFTFGKPSADHVIDRFLRDEYEDNNAEEESLEMLQLGEDDDDDEEEEGFWWEESIEGLSLEELDKYKASLEALRYNVAMKLEETMMRRDHERDFLSFI*

>ZaMADS67

MGRGKIAIKKIENTTTRQVTFSKRRAGLLKKTHELSVLCDAQIGLIIFSSTGKLCQYCTQPFRMEQIIERRLKVTGTRIPENDSREQLFNELAVLKKETRRLQLSMRRYTGEDLSSVPYDDLDELEQQLEHSVNKVRERKNELLQQQHDNLRRKERMLEEENSNMYRWIQEHRAAMEYQQAAMEAKPVEDHHHQQILDHFPFCGDHQQPSSVLQLATNNIPPQILPYHHLQLALPNLQDPNI*

>ZaMADS98

MGRGKIEIKKIENANSRQVTFSKRRAGLLKKAKELAILCDAEVAVIIFSNTGKLFEYSSSGMKRTLSRYNKCPDFSEAPVAEYETEKQDSKEVDGLKDTIAKLQTKQLQLLGKDLNGLSLKELQLLEQELDEGFLLVKEKKEQLLMEQLEQSRVQEQRAMLENETLRRQARYIFLNETRLFHYIYDL

>ZaMADS79

AYELSVLCDAEVALIVFSSRGRLYEYSNSNSIRSTVDRYKKACSDNSNSGSVTEINAQYYQQESAKLRQQIQMLQNSNRNLMGESVSSLTVKELKQLENRLERGITRIRSKKHEMLLAEIEFFQKREIELENESLSLRSKIAEVERFQQANMVNGEELNAIHALASRNLFSPLMEGGSGTAYSHPHPHPHTEKKMLHLG

>ZaMADS78

GKLYEFSSCSIHKTIERYQKRSKNIGISTKIVEEDHTQHVKEETSSMAKKLEFLEATKRKLLGEGLEPCSMDELQQLENQLERSLSRIRAIKDQQFREQIKNLKEKEKIVMEENMKLREKCGMQPRQASPEQEVFNIQSMDVDTELFIGPPERRVA

>ZaMADS93

MGRGRVQMKRIENKISRQVTFSKRRAGLLKKAHEISVLCDAEVALIVFSTKGKLFEYSTDSSMERILERYEKNAYVEQQLGTTDTQQQGCWSLEYPLLKNRIEVVERNIRNFMGEDLEPL

>ZaMADS91

YGRTLHHHHGLLPLPFLADRGRISGLGFGFGFGGEEEAGMVRGKTQMKRIENPTSRQVTFSKRRGGLLKKAFELSVLCDAEVALVIFSPRGRLYEFASGSMQKTIERYKTSTKDNIRSQTVQQDIEKIKADAEGLSKKLEALDAYKRKLLGYNLEECPIEELQSLEVKIEKSLLCIRARKAQLFEEQLAKLRQKEVTLRKEKEDLLGQRKNGPQLAAAAAAPVTVVAQNHPQPEPVQEKDEMEVETELFIGLPG

>ZaMADS86

MARERREIRRIESAAARQVTFCKRRRGLFKKAEELAVLCDADVALVVFSSTGKLSQFASSSMNEIIDKFSTHSKNLGKSDQQPALDLNLEHSKINSLTEQLAEASLHLRHMRGEELGGLSVGELQQMEKNLETGLQRVLCTKDRQFMQQISDLQQKGSQMVEENMRLRNQMPQVPTADMMAVADTENVVTEAVSSESVMTQGNGDGSDISL

>ZaMADS54

MGRGKIEIKRIENLNSRQVTFSKRRNGLLKKAKELSVLCDADVGVIVFSSTGKLYEFSSSSMDHILSRYNKGLDLESQTNPHEVHKAEQAQVPEVNALKDEFARLHLAYMQMNGKELDGLSFKELQQLEHQLSEGILYVKEKKEQVLLEQIKRSRLLEQNAILENETLWKQMEELRGSRSPLLEFDPLERRFSFKKSKTDHCHCTSIEDEDEDDHSDTSLHLG*

>ZaMADS66

MKLDSKEVDGLKDTIAKLQTKQLQLLGKDLNGLSLKELQLLEQELDEGFLLVKEKKEQLLMEQLEQSRVQEQRAMLENETLRRQVEELRGFFPSTECSVPAYLEYYPLGRKNSLMNQSSASPDVASDSAIEKGDSDTTLHLGLPSNIYHKTKAPEGESHSNDSRSQLGLL*

>ZaMADS56

MKEILEKHHMHSKNLERLDRPSLELQLVENNSYSRLSKEFSDKSHQLRQMRGEEIHGLSLEELQKLERSLEVGLKRVMDKKGEKIVAEINEYQRRGKVLMEENEHLRQRVAEISNAQRQIAQDSDNFYSEEGQSSESVTNISNSSNVPPPESESSDTSLKLGLPYVG*

>ZaMADS70

MKPIETFFSELRSSFSSCSRMPKMLERYQKCNYGAPEPNVSAREALELSSQQEYLKLKARYESLQRSQRNLLGEELGPLNSKELESLEIQLDMSLKQIRSTRTQYMLDTLTDLQHKEHFLNEGNKNLRQRLMEGYQVNTLQLHPSAEDCGYGFKPAHPQCDTFFQPLDCEPTLQIGYQGADQVSVVTAGPSVSNYMQGWLPC*

>ZaMADS94

MHTPVLFFLFTPFIISIIMDGTLSFFSPLSLFHESKSVSKTLERYQRCCFNPQDNNSIEHETQSWYQEVTKLKAKYESLQRTQRHLLGEDLAPLSVKELQNLEKQLEGALSMARQRKTQVMIEQMEDLRKKERQLGDINKQLRIKLETEGQSYKAIEDMWNSAASGAGNSNFHVHPSHDNSMNCDPEPVLQIGYQHYLPAEGSSLPKNMIGETNFIQGWLL*

>ZaMADS95

MLLQYHKNHRDNFFFFLASSNFGLFFFFGFCYFAAGLKMVRGKTQMRRIENATSRQVTFSKRRNGLLKKAYELSVLCDAEVALIIFSPRGKLYEFANSSMQETVERYLRHTKDTRTKQQPPEKHMQQHLKHEAASMAKKIELLEVSKRKLLGEGLASCTLEELQQIEHQLERSVSNVRARKNQVFKEQIEQLKEKGKVLEAENARLAGKCGMEQWQVSEQQRENFPSNENGTSTSDGTSTSDVETELFIGPPPERRTRRLPMSLHN*

>ZaMADS87

MKMFDQYQESLRVDLWTNHYAKIQETYMELKEINNKLRKYTRQMMEEDLDEVTIEELRALEQDMTTSVAIIRERKVLSLSISQLDLNES

>ZaMADS59

MQNKNRNMLGESLSVLSVKELKKLEDSLEKGISRIRSKKNELLFAEIEYMQKRVSTFYLYLFNSCLYLII*

>ZaMADS42

MAFPNELAREESSKRKMGRGKIEIKRIENTTNRQVTFCKRRNGLLKKAYELSVLCDAEVALIVFSTRGRLYEYSNNSVKSTIDRYKKASADTSNTGSASEANAQFYLQEANKLRAQISSMQNKNRNMLGESLSVLSVKELKKLEDSLEKGISRIRSKKNELLFAEIEYMQKREVDLHNNNQLLRAKIAENEREPQNANLMPGGSSYNVMQQSQVQPFDSRSYFQVDALQPTNHYPHQDQMALRFV

>ZaMADS29

MMGRVKLKIKKLENTNGRQATYAKRKHGIMKKANELSVLCDIDIMLLMYSPTGKPSLCNGKRSNIEEIIAKFAKLTPQERAKRKMEGLEALKKTFKKLDHDVNVQEFVGTSSQTIEDLSNQTRLLQSQLSEIHGRLSYWANPDKINSLEHLGQMENSLRESLNQLRMHKENLGKQQLMSLECTPQFQNGMHIPFRLGSEQQLQPPPWIPNNDNRHMVLPEDPNLLSHR*

>ZaMADS31

MGRGKIEIARIESRTNRQVTFSKRRGGLLKKAHELSVLCDAQIGLIIFSCSGKLTEFCSDSTSIDQIIRKYEAAKGTRINPADINNHDDTEEIYSELRRMRNETHNLELSLGRYTGDI

>ZaMADS25

MGRVKLEIKRIENTTNRQVTFSKRRNGLIKKAYELSILCDIDIALIMFSPSGRLSHFSGRKRIEDVFSRYVNLPDHEREHAIIFPDQGRHPDIQNKEYLLRTLQQLRSENDIALQLANPAAINNDIEELQQEIGRLQQQLQMAEDQIRIYEPDLLRITSMKDIESCEKNLVDTLTRVAQRKEYLLGNHMSSYDPSSMQQAMPTSFENEVVGWLPDGGQNQAQMFDASAPLNQLRDLSTTMYDPMLQGTSSNAGPHSIGECHITNHNGENFATWPQAYVSAGLHSAPISPSLYPQIQHTNEMLPREQMEMPISAQHVQVDNEAANYDQNRIPQVNGQQ*

>ZaMADS28

MVRGKTQMKLIENATSRQVTFSKRRNGLLKKACELSVLCEAEVALIIFSPRGKLYEFSSSRKLVYYLGFGAHRESPYIIQGSLSSPMK*

>ZaMADS34

MGRGKIEMKKIENATSRQVTFSRRRNGLLKKACELSVLCDAEVGAIIFSQKGRLSEFSSSEMQNTIERYYKYSKGKSDRPEMEQYMQVRPSKFESKYFYDVQGSEYTYSKT*

>ZaMADS39

MKKIENATSRQVTFSRRRNGFLKKACELSVLCDAGVGAIIFSQKRRLSEFSSSELVIQSIHHRYQILRRRVAAVQSASRQVLGG*

>ZaMADS23

MGRVRVQIKKIENKTYRHITFAKRKNGLLKKAYEISTLCDVEVALIIFSPAGKLILFYGNKRLDEILMHYIDLPQHQRGRLRDQELVRRLIAQLSLEAELCHHLASNNRYDRDVTMSIDSQLQEIEKEICIRSAELENVEKQLQYFLKSPSCIKSASEAKFLE

>ZaMADS32

MGRGRVELKRIENKINRQVTFAKRRNGLLKKAYELSVICDAEVALIIFSNGGKLYEFCSTSSLRSHLEILGSNSRRDNRRFYLFRLSSEGMIWRWSFQRFKKYMFFFVIKTLELTPITLFLFF*

>ZaMADS26

MGRGRVQLKRIENKINRQVTFSKRRSGLLKKAHEISVLCDAEVGLIVFSTKGKLFEYSTDSWYLYLIYLYLHLF*

>ZaMADS27

MGRGRVQMKRIENKISRQVTFSKRRAGLLKKAHEISVLCDAEVALIVFSTKGKLFEYSTDSSMERILERYEKNAYVEQQLLSTDAGLQVLTESNIPHFYLQNLNYIC*

>ZaMADS24

MGCGKIEIKRIENLNNRQVTYSKRRNGLTKKIKEIAILCDANAVVIIVPTSGKIHEYCSAPYGFFFLFNFSKSFL*

>ZaMADS37

MGRGTVELKRIESKANRQVTFSKRKNGILKKALELSILCDAEIALIIFSPSGKPYRYASDNNNPERIIARYRREVGLLSNSANHESFGLMQVLWSSTFFFFFFEMGF*

>ZaMADS20

MGRRKIEIKAIKDDRNRSVTFLKRKGGLFKKAHELSVLCSVDVAVFIFGNNKKLYEYSSTDMRELIHRYQYHGGPSEHKGPSDFNGGNDDDEDEENDGTPPHGPEVVENQMMPPHAYGQHQPPFPQIRHHTPSASPPIGNGGPFQAHPGHPIQRQHTPQPSIGSRPASRTDMRRMGPGMVQPPPPPGPPHPGMNYMPNPPIYNSPHPPGLIPQHGPHPQYAYHQQPSHMQQPGPYMDDRRSPMPSPMPPAYTSQPPSQPIQAPVRPTPSPQPPQQQLPPNMSQMSPPPPQPERRLHDPPPPPPVEPKTEPQERPQPPLLNTDSAIKKLPQRKSHSIFTPIEENRSILSQHLASFTSESNKSESAAAAAAANAANAANRSQSVDVAALNRAADGPKSSPHLPQRASTQTDEKSRTVSLSSIPETTLTPPSRSNSAKAGGPGGARPRGPRLTVQIPDGGSEGGGSARTAESNSPRVATETTTQAPQRHNSQSSLVLPPPSPSASAILSAGATGPPNPFARPPPQQNVNGDTPVSALPSRFLTNELLPSPSSFYPDWNFRGGDSNTLPSPLNFATPVVGSGPSFLRDDLNTTPNANSNASKDRDPLPGSNGSLGQNLSVAPSNSTATKRKTPEPGATTQSDPADESEPKRLKVEE*

>ZaMADS33

MGRRKLTIRRLENMKARQTKYSKRKIGILKKAEELAVLCDIDLVLLMFSPTGKPSLCVGQNKDLSTVLEKLAKMSVEDREERRGYTMKLLKKIYANSEVDPRNFSLDRYDALKLHEDQLRELKDKLAEKTRILREWMNPHNVKDIAQINIMEEHLIGSLHKIRNKRRQLIEEQQQRYEPSQAIKDPQI*

>ZaMADS30

MMGRVKLKIKKLENTNGRQATYAKRKHGIMKKANELSILCDIDIVLLMFSPTGKPSLCNGKRSSIEEVIEKFAQLTPQERAKRKLESLEALKKTFKKLDHDVNVQGFLGTSSQTIEELTNQTRLLQSQLSEIHGRLSYWANPDKINNVEHLGQMENSLRESLNQLRMHKFQNGIHMPFRLDSEQQLQPLPWIPNNDNRHMVLPEDPNLLSHSS*

>ZaMADS41

MGRVKLKIKKLENTNCRQATYAKRKHGIMKKANELSILCDIDIVLLMFSPTGKPSLCNGNEEVIEKFAQLTPQERAKRKLESLEALKKTFKKLDHDVNVQGFLGTSSQTIEELTNQTRLLQSQLSEIHGRLSYWANPDKINNVEHLGQMENSLRESLNQLRMHKENIGKQQLMSLDCTNQFQNGMHIPFRLGSEQQLQHLSWIPNNDDRHMVLPEDPNLLNDNRHMVLPEDPNLISHRDVECSGSSSFGSYPGYFGTGKSSEISNSGLESSLLSELSGTASLRLQLGGQCSYLPYNVNLLCDKYQPVSEMNIQENPVDYHVTGSLEVPRPGFDTTPGSWASTSGPSEVTLFDEHLYSQQLN*

>ZaMADS40

MGRGRVQLKRIENTINRQVTFSKRRAGLLKKAHEISVLCDAEVAVIIFSHKGKLFEYSTDSCMEKILERYERYSYAERQLVAPDPESQGNWPLEYHKLKSKIDLLQRNQRQYLGEDLGSLSLKDIHHLEQQLDSALKRIRSRRDQVVQESISELQKKEKAIQEQNNLLAKQITETEKAAALQAQWGNQIQNQVPNTLSFLLPQPQPQPPPCLSIGNAFQEDNVEMRRNELDLTRMLR*

>ZaMADS21

MGRGKITIRRIDNSTSRQVTFSKRRNGLLKKAKELAILCDAEVGAIIFSSTGKLYDFASTRSFNLFFNLIYLFKIDRSQYHDDYLLCDPYYAMS*

>ZaMADS97

MLLSEAVICISLAYLINILYNIQETTSRACATYPNRQMMGEELSGLSVKDLQNLENQLEMSLRGVRMKKV

>ZaMADS22

MGRGKIMIRRIDNSTSRQVTFSKRRSGLLKKAKELAILCDADVGVIIFSSTGKLYDFASTRSFILISFFCLRSQISIS*
